# Phylogeny-guided discovery of a promiscuous P450 macrocyclase for the production of diverse atropopeptides

**DOI:** 10.1039/d5sc03525b

**Published:** 2025-08-06

**Authors:** Bin Tan, Peter Breunig, Lamia Arbib, Yuya Kakumu, Friederike Biermann, Kornelia Hardes, Jasmin Hefendehl, Eric J. N. Helfrich

**Affiliations:** a Institute for Molecular Bio Science, Goethe University Frankfurt Max-von-Laue Strasse 9 60438 Frankfurt am Main Germany eric.helfrich@bio.uni-frankfurt.de; b Institute of Cell Biology and Neuroscience, Goethe University Frankfurt and Buchmann Institute for Molecular Life Sciences 60438 Frankfurt am Main Germany; c Department of Bioresources, Fraunhofer Institute for Molecular Biology and Applied Ecology Ohlebergsweg 12 35392 Giessen Germany; d Senckenberg Society for Nature Research Senckenberganlage 25 60325 Frankfurt Germany

## Abstract

Cyclic peptides exhibit diverse bioactivities and are distinguished by their enhanced cell permeability, improved proteolytic stability, and increased binding affinity due to their conformational rigidity. Despite significant advancements in peptide synthesis, the production of complex cyclic peptides remains a challenge. Nature has evolved diverse strategies for peptide cyclization, with an ever-growing repertoire of characterized cyclases involved in the biosynthesis of ribosomally synthesized and post-translationally modified peptides (RiPPs). These enzymes convert linear precursor peptides into complex (poly-)cyclic structures. The discovery of the atropopeptides has significantly expanded the chemical diversity of RiPPs with unique (poly-)cyclic structures. In this study, we employed a phylogeny-guided approach to identify a substrate-promiscuous cytochrome P450 macrocyclase that catalyzes the formation of cyclic peptides through atropospecific C–N or C–C bond formation between aromatic amino acid side chains. Combinatorial biosynthetic studies revealed that ScaB encoded in the scabrirubin biosynthetic gene cluster efficiently cyclizes a wide range of atropopeptide precursor peptides. Furthermore, extensive site-directed mutagenesis studies of the tetrapeptide core sequence further expanded the diversity of atropopeptides. Notably, three tested atropopeptides show antiviral activity and one of the non-natural atropopeptides displays anti-inflammatory activity. Our findings establish a broadly substrate-tolerant atropopeptide-modifying P450 as a versatile biocatalyst for the synthesis of bioactive, biaryl-bridged macrocyclic peptides.

## Abstract 2

Our findings establish a substrate promiscuous atropopeptide-modifying P450 macrocyclase as a versatile biocatalyst for the biosynthesis of bioactive, C–C or C–N biaryl-bridged macrocyclic peptides with antiviral and anti-inflammatory properties.

## Graphical abstract

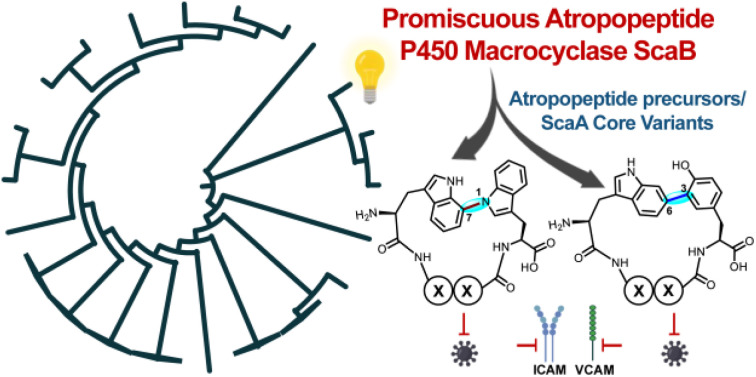

## Introduction

**Fig. 1:**
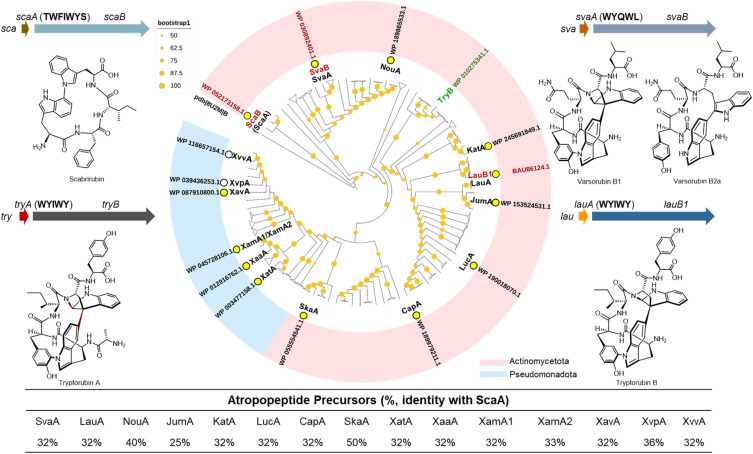
Phylogenetic tree of atropopeptide-modifying P450s. The maximum likelihood tree was rooted using P450_Blt_ 8U2M_B as outgroup.^30^ Bootstrap values (orange circles) are based on 1000 bootstrap replicates. Bootstrap values higher than 50 are displayed. The *sca*, *sva* and *lau* BGCs and their corresponding products, as well as tryptorubin A and its BGC *try* are displayed. Additionally, the P450s ScaB, SvaB and LauB1 used for combinatorial biosynthesis are highlighted in red and the P450 TryB is highlighted in green. The 15 tested atropopeptide precursors are labelled at the position of their corresponding P450 and their sequence identity with ScaA is shown. The triangles represent collapsed clades. The expanded tree with exact bootstrap values is depicted in Fig. S1.

Cyclic peptides have long been regarded as important resources for pharmaceuticals.^1^ The structural rigidity of cyclic peptides imparts favorable biological properties including enhanced cell permeability,^2^ improved proteolytic stability,^3–5^ and increased binding affinity.^6^ Cyclic peptides display a broad range of bioactivities, including antifungal, antiviral, antitumor and anti-inflammatory properties.^1,6,7^ More than 40 cyclic peptides are currently used as drugs with an average rate of one cyclic peptide being approved for clinical use annually.^6,8^ Inspired by the highly promising therapeutic potential of cyclic peptides, substantial efforts have been dedicated to their exploration. Cyclic peptides can be chemically synthesized or obtained through biotechnological processes. Chemical peptide synthesis excels at obtaining linear peptides but macro/polycyclic peptides remain challenging to synthesize due to the difficulties in intramolecular macro/poly cyclization of linear peptides in a chemo-, regio-, and stereospecific manner.^9,10^ For instance, the atroposelective total synthesis of the hexapeptide tryptorubin A (Fig. 1) involved coupling a dipeptide and a tripeptide fragment. Each fragment harbored one of the characteristic biaryl bridges that were installed early in the synthetic route. The two fragments were joined *via* biaryl bond formation, and the synthesis was completed by incorporating the final amino acid to link the peptide fragments. The synthesis of tryptorubin A serves as just one example of the complexity and non-linear nature of synthesizing complex cyclic peptides.^11^ Alternatively, complex cyclic peptides can be obtained through biosynthesis or combinatorial biosynthetic strategies.^12,13^ A key requirement for leveraging biological strategies to generate diverse cyclic peptides for drug development is the identification of highly promiscuous macrocyclases.

Naturally occurring complex cyclic peptides are predominantly derived from non-ribosomal peptide synthetase (NRPS) and ribosomally synthesized and posttranslationally modified peptide (RiPP) biosynthetic pathways. NRPSs are large multi-modular mega-enzymes that assemble complex peptides in an assembly line-like fashion.^14^ Cyclization of NRPS-derived peptides is typically catalyzed by thioesterase, condensation, or reductase domains resulting in the formation of macrolactones, macrolactams or cyclic imines.^15–18^ RiPP biosynthetic gene clusters (BGCs) on the other hand are typically much smaller in size and frequently harbor three types of genes: one or multiple small precursor genes, genes encoding tailoring enzymes and peptidases. The precursor peptide, composed of a leader and core peptide sequence, is first ribosomally synthesized, followed by a series of posttranslational modifications at the core peptide by tailoring enzymes before the mature RiPP is liberated from its leader sequence by proteolytic cleavage.^19,20^ Amongst the characterized tailoring enzymes are a large number of enzymes responsible for (macro-) cyclization. Common macrocyclization reactions in RiPP biosynthesis involve intramolecular Michael-type additions forming (methyl)lanthionine [(Me)Lan] bridges in lanthipeptides,^21^ [4 + 2] aza-cycloadditions generating pyridine cores in thiopeptides^22,23^ and the radical SAM enzyme^24,25^ or cytochrome P450 ^26^–mediated formation of C–C, C–S, C–N and C–O crosslinks in diverse RiPP families, including but not limited to sactipeptides, darobactins, and atropopeptides.

Atropopeptides are distinguished by their unique (poly)cyclic peptide scaffolds featuring axially chiral (atropisomeric) biaryl linkages. The first characterized atropopeptide, tryptorubin A,^11,27^ is a hexapeptide and features C–C and C–N biaryl crosslinks between aromatic sidechains and an additional C–N bond between an aromatic sidechain and the peptide backbone. Although tryptorubin A features a complex three-dimensional shape, its BGC only contains two genes: a gene encoding the precursor peptide and a gene encoding a cytochrome P450 monooxygenase, which was shown to install all three crosslinks in the hexapeptide.^28^ We recently identified more than 650 atropopeptide BGCs using a machine learning-based genome mining algorithm.^29^ Four of these BGCs (*jum*, *sca*, *sva*, *lau*) were characterized, leading to the discovery of structurally distinct atropopetides which either feature polycyclic structures or a monocyclic structure with C–N, C–C or C–O biaryl crosslinks and varying peptide length.^29^ The simple biosynthetic pathways that result in complex (poly-)cyclic peptides inspired us to further explore the potential of atropopeptide-modifying cytochrome P450 macrocyclases to act as versatile biocatalysts for the formation of complex cyclic peptides.

In this study, we used a hypothesis-driven and phylogeny-guided approach to identify a promiscuous atropopeptide-modifying macrocyclase. We found that ScaB, encoded in the scabirubin BGC, exhibits broad substrate promiscuity towards other precursor peptides and the engineered native core peptide, resulting in diverse cyclic peptides with C–N or C–C biaryl crosslinks. Moreover, three cyclic peptides showed antiviral activity and one of them displayed anti-inflammatory activity. Our study demonstrates the catalytic capability of ScaB in creating diverse cyclic peptides with biaryl crosslinks, highlighting its potential as a biocatalyst for the generation of bioactive macrocyclic peptides with potential pharmaceutical applications.

## Results

### Phylogeny-guided discovery of a promiscuous atropopeptide macrocyclase

It has been frequently observed that ancestral proteins often exhibited broader substrate promiscuity than their extant counterparts,^31–36^ although exceptions have also been reported.^37,38^ Based on this empirical observation, we hypothesized that the early-diverging atropopeptide-modifying P450 may retain a broader substrate promiscuity than more recently evolved members of this family. To test this hypothesis, we constructed a rooted phylogenetic tree of 288 non-redundant atropopeptide-modifying P450 enzymes, using 8U2M_B (P450_Blt_)^30^ as the outgroup (Fig. 1 and S1). P450_Blt_ catalyzes biaryl crosslink formation in biarylitide biosynthesis and acts on short pentapeptide substrates to yield tripeptides with a biaryl linkage between Tyr and His. In contrast, atropopeptide-modifying P450s act on much larger precursor peptides and produce significantly larger and structurally more diverse macrocyclic products. Due to these functional and phylogenetic differences, P450_Blt_ was considered an appropriate outgroup. We found that the P450 ScaB, that we recently reported to catalyze the atroposelective cyclization of the monocyclic peptide scabrirubin,^29^ was located closest to the root position (Fig. 1). Therefore, we reasoned that ScaB might be a particularly promiscuous atropopeptide-modifying P450. To test our hypothesis, we selected the *sca* BGC encoding ScaB, along with two recently characterized atropopeptide BGCs, *sva* and *lau*, whose corresponding P450s (SvaB and LauB1) are positioned relatively far from the root, for combinatorial biosynthesis. Given that these three BGCs produce structurally distinct atropopeptides—scabrirubin, varsorubin B1/B2a, and tryptorubin B (Fig. 1)—they provide a valuable framework to investigate and compare the substrate specificities of their respective P450s *via* combinatorial biosynthesis.

**Fig. 2:**
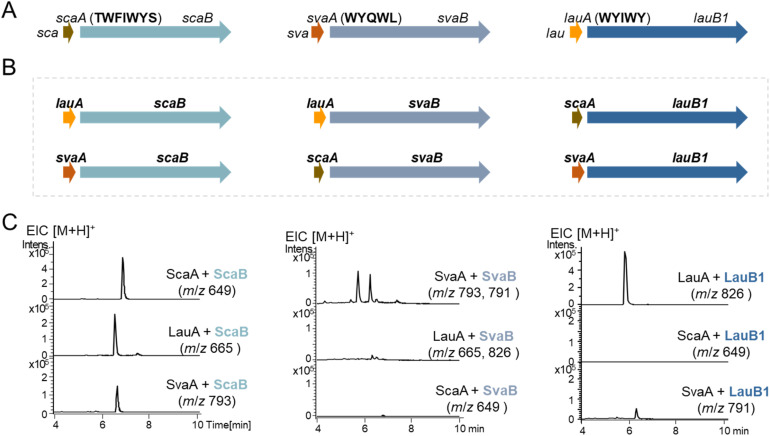
Combinatorial biosynthesis of the P450s ScaB, SvaB and LauB1 with each other's precursor peptides. (A) *sca*, *sva* and *lau* BGCs. (B) Different combinations of cytochrome P450s with different precursor peptides. (C) Extracted ion chromatogram (EIC) of compounds generated from extracts of recombinant strains.

Combinational co-expression of cytochrome P450s and precursor peptide-encoding genes in *Streptomyces albus* J1074 (Fig. 2B) demonstrated that ScaB efficiently cyclizes LauA and SvaA from the *lau* and *sva* BGCs, respectively, which both share only 32% amino acid identity with ScaA (Fig. 1 and 2C), as indicated by a 2 Da mass loss compared to their corresponding linear core peptide WYIW and WYQWL (Table S1). Although LauB1 and SvaB show low efficiency in modifying each other's precursors, they cannot cyclize the ScaA core peptide (Fig. 2C). These results demonstrate that ScaB exhibits broader substrate tolerance than LauB1 and SvaB, consistent with our hypothesis that the early-diverging atropopeptide-modifying P450 possesses higher substrate promiscuity.

**Fig. 3:**
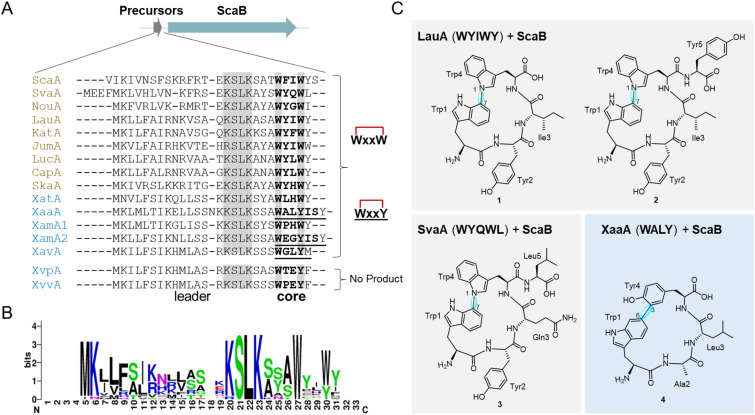
Combinatorial co-expression of genes encoding atropopeptide precursor peptides and ScaB. (A) 13 atropopeptide precursors were selected along with SvaA and LauA and co-expressed with *scaB*. The conserved KLSK motif and the crosslink-forming aromatic amino acids are highlighted with a grey background. Core peptides are highlighted in bold. The precursor peptides that yield the unusual Trp–Tyr crosslinks are underlined in their core peptide regions. 13 out of 15 combinations of precursor peptides with ScaB led to the generation of the corresponding cyclic peptides; (B) sequence logos of 15 selected atropopeptide precursor peptides; (C) the structures of 1, 2, 3 and 4 from the combinatorial co-expression of *lauA*, *svaA* or *xaaA* with *scaB*.

To further investigate the substrate promiscuity of ScaB, 13 additional atropopeptide precursors (Fig. 1), whose corresponding P450s are distributed across the phylogenetic tree of atropopeptide-modifying P450s, were selected and co-expressed with *scaB*. Among these precursors, seven (XatA, XaaA, XamA1, XamA2, XavA, XvpA and XvvA) are from Pseudomonadota, a phylum from which no atropopeptides have been reported to date. Their corresponding P450s are located furthest away from the root of the tree (Fig. 1 and 3A). These selected precursor peptides share 25% to 50% identities with ScaA (Fig. 1). 11 out of the 13 tested precursors, including five derived from Pseudomonadota, were successfully modified by ScaB (Fig. 3A), demonstrating its high substrate tolerance, even toward substrates whose associated P450s are phylogenetically distant from ScaB. The ScaB mediated macrocyclization primarily resulted in the formation of tetrapeptides harboring either the WxxW or WxxY motif (Fig. 3A), pentapeptides (*e.g.*, WYQWY and WPHWY) and rarely in the formation of a hexapeptide (*e.g.*, WEGYIS) (Table S1), as indicated by liquid chromatography-high resolution electrospray ionization mass spectrometry (LC-HRESIMS) analysis (Fig. S2 and Table S1). We previously proposed that unknown proteases may cleave the N- and C-terminal amino acid(s) following macrocyclization, thereby generating the observed tetrapeptides.^29^ These newly generated peptides showed a 2 Da mass loss compared to their corresponding core peptide sequence (Table S1), indicating the formation of monocyclic peptides. MS/MS analysis suggested that the crosslinks are formed between the side chains of the first and the fourth amino acid (Fig. 3A and S3–S15, Table S1). These results highlight the high substrate promiscuity of ScaB toward diverse precursor peptides (Fig. 3A).

Since the tested substrates possess two conserved motifs: ‘KSLK’ and ‘WxxW/WxxY’ (Fig. 3A), we were curious whether these two conserved motifs are sufficient for the recognition of a precursor by the P450 ScaB. To answer this question, two N-terminally truncated precursors, ScaA_16–28_ where the last 13 amino acids, including the conserved ‘KSLK’ motif, were retained, and ScaA_20–28_ where only the last nine amino acids were retained (Fig. S16), were co-expressed with ScaB. The results showed that scabrirubin can be generated with low yield from the combination of ScaA_16–28_ and ScaB, but no product was detected from the combination of ScaA_20–28_ and ScaB (Fig. S16), suggesting that in addition to the ‘KSLK’ motif, other regions of the leader peptide are also critical for the efficient cyclization by ScaB.

### Identification of crosslink patterns in the non-natural atropopeptides generated from combinatorial biosynthesis

The HRESIMS analysis of the products from different combinations of atropopeptide precursors with ScaB indicated two main types of cyclic peptides Trp-x-x-Trp and Trp-x-x-Tyr. The cyclic peptides Trp-x-x-Trp were mainly observed from the combination of Actinomycetota-derived precursors and ScaB while cyclic peptides Trp-x-x-Tyr were mainly found from the combination of Pseudomonadota-derived precursors and ScaB (Fig. 3A). To determine the crosslink pattern in these products, we selected three recombinant strains, which harbored the combinations of Actinomycetota-derived precursors LauA (WYIWY) or SvaA (WYQWL), or Psedumonadota-derived precursor XaaA (WALYISY) with ScaB, respectively, and subsequently performed large-scale fermentation of these recombinant strains, followed by extraction, chromatographic purification and structure elucidation to identify the corresponding products.

The cyclic tetrapeptide scabrirubin CB-1 (for combinatorial biosynthesis-1) (1) (*m*/*z* 665.3083 [M + H]^+^, Fig. S17) and cyclic pentapeptide scabrirubin CB-2 (2) (*m*/*z* 828.3718 [M + H]^+^, Fig. S24) were isolated from *S. albus*/pUWL-LauA + ScaB (Fig. 3C). Both 1 and 2 exhibited a 2 Da mass loss compared to the core peptide WYIW (cal. for 667.3238 [M + H]^+^, Table S1) and WYIWY (cal. for 830.3871 [M + H]^+^, Table S1), suggesting the presence of monocyclic peptides. 1D and 2D NMR spectroscopic data revealed a C–N biaryl crosslink between Trp-1 and Trp-4 in 1 and 2 (Fig. 3C, Tables S2–S3 and Fig. S18–S30). The cyclic pentapeptide scabrirubin CB-3 (3) (*m*/*z* 793.3663 [M + H]^+^, Fig. S31) was isolated from *S. albus*/pUWL-SvaA + ScaB (Fig. 3C). Compared to the mass of the core sequence (WYQWL, cal. for 795.3824 [M + H]^+^), 3 exhibited a 2 Da mass loss, suggesting a monocyclic structure. Similar to the crosslink pattern of 1 and 2, a C–N biaryl crosslink in 3 was formed (Fig. 3C, Table S4, and Fig. S32–S37). The cyclic tetrapeptide scabrirubin CB-4 (4) (*m*/*z* 550.2659 [M + H]^+^, Fig. S38) was isolated from *S. albus*/pUWL-XaaA + ScaB (Fig. 3C). 4 exhibited a 2 Da mass loss compared to the core peptide sequence (WALY, cal. for 552.2816 [M + H]^+^, Table S1), suggesting a monocyclic peptide. In 4, a C–C biaryl crosslink was formed between Trp-1 and Tyr-4 (Fig. 3C, Table S5 and Fig. S39–S44). Notably, the carbon atom involved in crosslink formation at the Trp-1 residue is C-6 in 4, which is distinct from C-7 in 1, 2 and 3. To our knowledge, a biaryl linkage between C-6 of Trp and C-3 of Tyr (4) has not been previously observed in RiPPs, representing a novel biaryl crosslink. Rubrin also features a Trp–Tyr biaryl linkage^39^ (Fig. S69). However, in rubrin, the carbon atom involved in biaryl bridge formation is C-7 at the Trp indole ring rather than C-6 as in compound 4.

These results demonstrate that ScaB catalyzes the formation of C–N biaryl crosslinks between two tryptophan residues as shown in 1, 2 and 3 or C–C biaryl crosslink between tryptophan and tyrosine as shown in 4. We thus proposed that products modified by ScaB with the core peptide motif WxxW possess a C–N biaryl crosslink, while those with the core peptide motif WxxY contain a C–C biaryl crosslink.

### Site-directed mutagenesis of precursor peptides

**Fig. 4:**
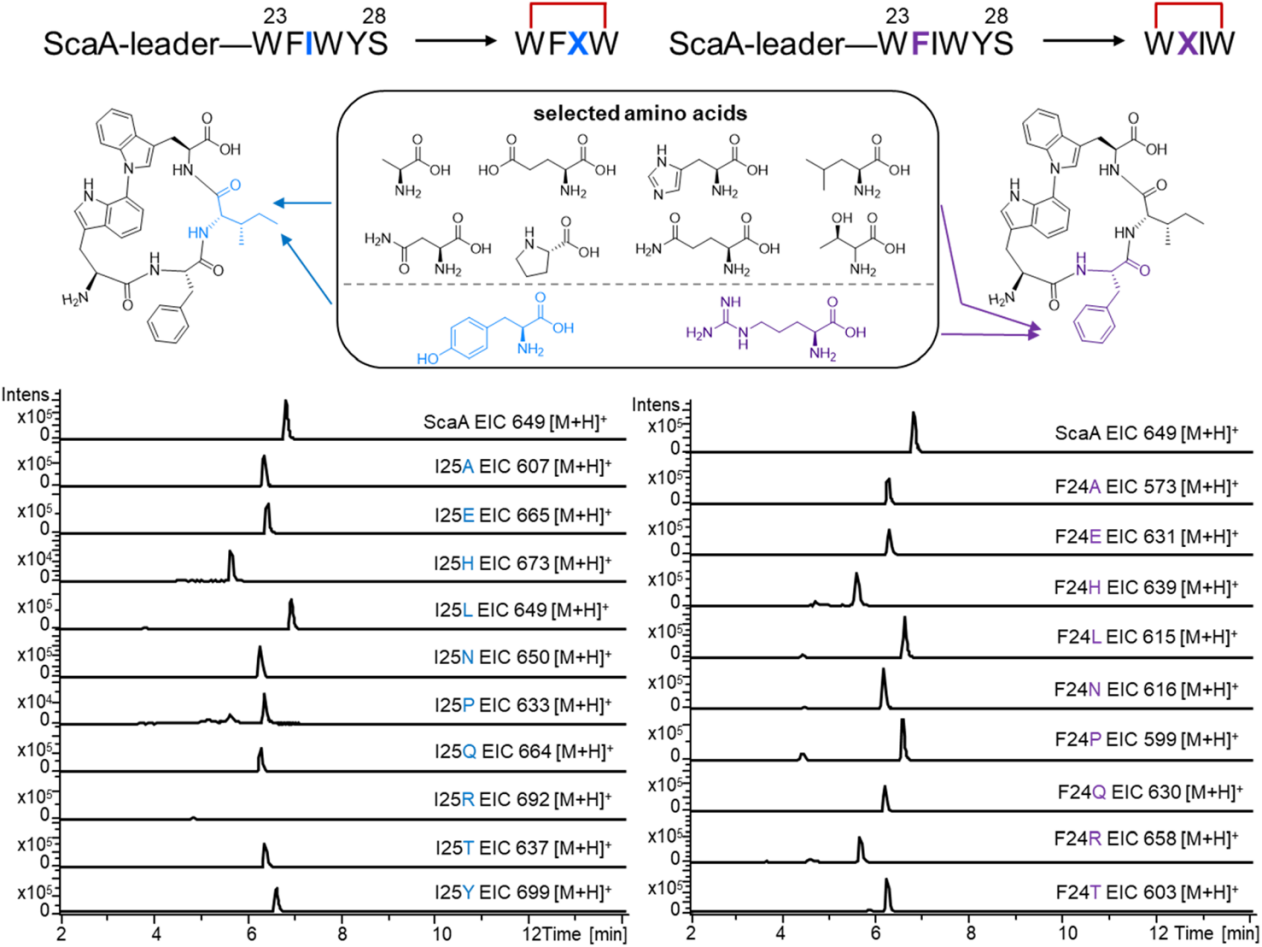
Site-directed mutagenesis of ScaA core peptide. EIC of non-natural atropopeptides generated from engineered ScaA core peptides.

To further explore the substrate promiscuity of ScaB in creating cyclic tetra and pentapeptides, we performed site-directed mutagenesis on the amino acids that are not involved in biaryl-bridge formation in the ScaA core peptide. Amino acids with different properties, including acidic (E), basic (H, R), polar (Q, N, Y, T) and non-polar (L, A, P), were selected to substitute these two amino acids between the two conserved tryptophans in the ScaA core peptide. Specifically, ScaA-24F was mutated to ScaA-F24X (X = H, N, P, R, E, Q, T, L, A) and ScaA-25I was mutated to ScaA-I25X (X = A, L, P, Q, T, E, H, N, Y, R). The results showed that almost all engineered precursor peptide variants (except ScaA-I25R) are recognized by ScaB and the corresponding cyclic tetrapeptides are generated with high yield (Fig. 4, S45–S62), highlighting the potential of ScaB to atroposelectively generate tetrapeptides with biaryl crosslinks.

Our observation that ScaB can modify the precursor peptides XaaA, XamA2 and XavA, which contain the WxxY motif instead of the canonical WxxW motif, suggests that ScaB accommodates variation in the crosslinking residues. To further investigate the catalytic promiscuity of ScaB, we replaced the amino acids involved in crosslink formation in both the native precursor ScaA and the non-native precursors XaaA and XamA2. Mutated precursor peptides included ScaA (W23Y, W26Y, W26H, W26S, W26T), XaaA (W23Y, W23H, Y26W, Y26H), and XamA2 (W22Y, W22H, Y25H, Y25W). The results revealed that ScaB can efficiently modify XamA2-Y25W and XaaA-Y26W (Fig. S63–S65). Moreover, ScaB also modified ScaA-W26Y and even ScaA-W23Y where the highly conserved first Trp was replaced by Tyr albeit at low titers, displaying a level of catalytic promiscuity (Fig. S63). The remaining combinations of mutated core peptides with ScaB did not result in the production of cyclic peptides (Fig. S63). In summary, the cyclization of atropopeptide precursors in which the residues involved in biaryl bridge formation have been exchanged indicates that ScaB exhibits catalytic promiscuity. The observed ability of ScaB to efficiently modify XamA2 (WEGYISY), XaaA(WALYISY), and XavA (WGLYM) but not the modified native precursor ScaA-W26Y (WFIYYS) warrants further investigation.

### Bioactivity of ‘non-natural’ scabrirubin analogs

**Fig. 5:**
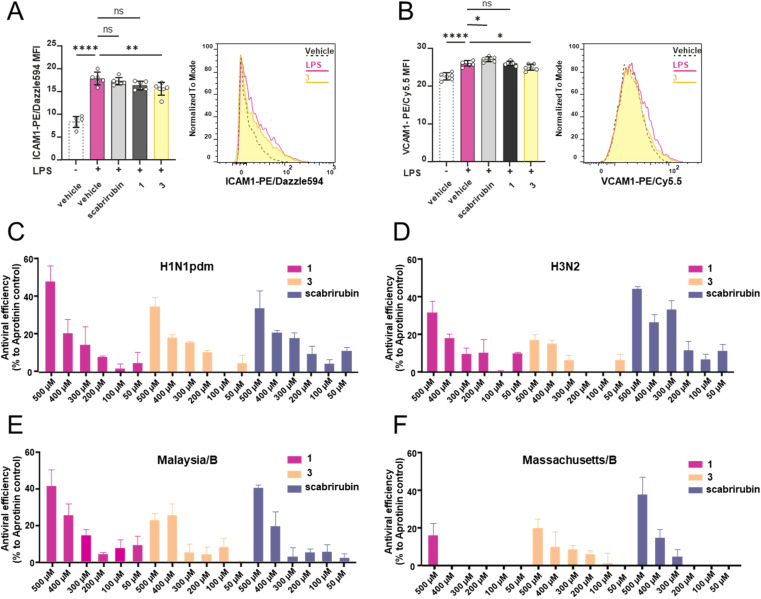
The anti-inflammatory and antiviral activities of scabrirubin, scabrirubin CB-1 (1) and scabrirubin CB-3 (3). Mean fluorescence intensity (MFI) for ICAM1 (A) and VCAM1 (B) with each individual technical replicate (*n* = 6 per condition). Error bars represent the standard deviation. * = *p* ≤ 0.05; ** = *p* ≤ 0.01; **** = *p* ≤ 0.0001. Histograms display the fluorescence intensity distribution of representative samples of the indicated conditions. Screening of atropopeptides against H1N1pdm (C), H3N2 (D), Malaysia/B (E) and Massachusetts/B (F) in MDCK II cells.

To investigate the bioactivity of the newly generated cyclic peptides, we first evaluated the antimicrobial activity of compounds 1, 3 and 4 against all ESKAPE pathogens. None of the compounds showed antimicrobial activity. Subsequently, we evaluated their anti-inflammatory and antiviral activities. Compounds 1, 3 and scabrirubin were tested against LPS-stimulated brain endothelial cells (b.End.3) (Fig. S66). Stimulation of b.End.3 cells led to a marked increase in the expression of the cell adhesion molecules ICAM1 and VCAM1, which are commonly used markers for vascular inflammation.^40–42^ Upon exposure to 3, both ICAM1 and VCAM1 expression was significantly reduced (Fig. 5A and B). Treatment with 1 or scabrirubin resulted in a slight reduction of ICAM1 expression and unchanged (1) or slightly elevated VCAM1 levels (scabrirubin). These results show that the non-natural atopopeptide 3 was more effective compared to 1 and scabrirubin at reducing inflammation.

In an antiviral screen, the impact of compounds 1, 3, and scabrirubin on the replication of four influenza virus strains was assessed. MDCK II cells were infected with: A/Hamburg/05/2009 (H1N1pdm), A/Hessen/1/2003 (H3N2), B/Malaysia/2506/2004 (Malaysia/B), or B/Massachusetts/71 (Massachusetts/B). The tested compounds did not show cytotoxicity toward MDCK II cells at the concentration used (Fig. S67). Aprotinin was used as the positive control, and its antiviral activity was normalized to 100%. The antiviral efficacy of the tested compounds was compared to that of aprotinin. The results demonstrated a dose-dependent antiviral effect for all tested natural and non-natural atropopeptides (Fig. 5C–F). Notably, 1 and scabrirubin exhibited comparable antiviral efficacies, while compound 3 demonstrated slightly reduced activity.

### Discussion

A commonly proposed model of enzyme evolution suggests a trajectory from generalist enzymes with high substrate promiscuity to specialists with increased substrate specificity,^43,44^ though alternative evolutionary trajectories have also been described.^45–48^ This model is supported by multiple studies involving ancestral sequence reconstruction.^31–36,49,50^ Based on this framework, we hypothesized that early-diverging proteins may retain broader substrate tolerance. Consistent with this hypothesis, we observed that ScaB, located near the root of the phylogenetic tree of atropopeptide-modifying P450s, exhibited higher substrate tolerance toward non-native precursors than other members of this family. Notably, ScaB was able to efficiently modify a broad range of precursor peptides from across the phylogenetic tree of their associated P450s, consistent with the possibility that early-diverging enzymes may retain higher substrate promiscuity. However, further studies are necessary to determine whether this property is conserved across other early-diverging members of this enzyme family. Ancestral sequence reconstruction could offer a valuable strategy to investigate this question further. Structurally, ScaB appears to have a larger substrate-binding pocket than the related P450s SvaB and LauB1, based on AlphaFold^51^ models of the P450s with the corresponding precursor peptides and binding pocket size estimations using POCASA^52^ (Fig. S68). This predicted structural feature may contribute to the observed promiscuity of ScaB. Nonetheless, further experiments are required to confirm its relevance and contribution to ScaB's substrate promiscuity.

Biaryl crosslinks installed by cytochrome P450s are important structural features found in many notable natural products, including the NRPS-derived glycopeptide antibiotic vancomycin,^53^ the CDPs-derived 2,5-diketopiperazine mycocyclosin^54^ and an increasing number of RiPPs (Fig. S69).^39,55–63^ In RiPP biosynthesis, biaryl crosslink patterns installed by P450s include carbon–carbon, carbon–nitrogen, and ether bonds formed between tryptophan, tyrosine and histidine residues, with crosslinks involving various position within these residues (Fig. S69). These modifications significantly enhance the structural diversity of RiPPs.

The substrate tolerance of several biaryl-crosslink forming P450s in RiPPs biosynthesis have been explored.^39,57,59,64^ For example, the substrate tolerance of the P450 macrocyclases SroB, PruB and SlpB in the biosynthesis of the cyptides roseovertin, rubrin and lapparbin was investigated through leader peptide swapping. SroB was shown to accept the non-native precursor PruA, PruB tolerated a hybrid precursor containing a non-cognate leader fused to its native core peptide while SlpB only recognized its native precursor, indicating varying degrees of substrate tolerance among these P450s.^39^ The catalytic promiscuity of the P450s MciB, KstB and ScnB in the biosynthesis of the biarylitide micitide 982, the atropopeptide kitasatide and bitryptide strecintide 839 was explored by mutating the ring-forming residues to other aromatic residues. The P450s KstB and ScnB were shown to modify substrates when the ring-forming residues Trp and Tyr are substituted to Tyr or Trp, respectively, indicating a degree of catalytic promiscuity.^57^ The substrate tolerance of the P450s SanB, SyrB and CreB in the biosynthesis of the cittilin-type RiPPs shandoamide, syrinamides and citreamide was evaluated by combinatorial biosynthesis. SyrB modifies SanA while SanB and CreB do not modify non-native precursors, suggesting limited substrate flexibility. While CreB tolerates mutations in crosslink forming residues, SyrB does not modify other core peptide sequences with substitutions at crosslinking positions, indicating a level of catalytic flexibility of CreB.^59^ P450_Blt_ that catalyzes the formation of the biaryl crosslink in the biosynthesis of the biarylitide YLH possesses a high level of substrate tolerance. Through site-directed mutagenesis at the core peptide, diverse Tyr-x-His and Tyr-x-Trp tripeptides with C–N biary crosslinks were generated.^64,65^ More recently, active-site engineering of the biarylitide-modifying P450_Blt_ further expanded its catalytic scope, resulting in the formation of C–C crosslinks in His-x-Tyr and C–O crosslinks in Tyr-x-Tyr tripeptide motifs.^66^ In our study, we systematically investigated the substrate promiscuity of ScaB toward various atropopeptide precursors across the phylogenetic tree using combinatorial biosynthesis and site-directed mutagenesis. ScaB cyclized 13 out of 15 tested precursors, forming either C–N biaryl crosslinks between Trp-x-x-Trp residues or C–C biaryl crosslinks between Trp-x-x-Tyr residues, highlighting its broad substrate tolerance and a level of catalytic promiscuity. Notably, the crosslink formed between Trp C-6 and Tyr C-3 in peptides with a Trp-x-x-Tyr motif represents a previously unreported linkage in RiPP structures. Moreover, ScaB also tolerated substitutions in amino acids involved in biaryl crosslink formation. It accepted Trp-to-Tyr or Tyr-to-Trp substitutions at position 4 of the core peptide, and substitution of the first Trp conserved in all atropopeptides core peptides with Tyr also resulted in the formation of cyclic peptides, albeit at very low titers. While RiPP-modifying P450s such as ScnB and CreB exhibit broader catalytic flexibility, ScaB is distinguished by its remarkable substrate promiscuity and moderate catalytic versatility, underscoring its potential as a valuable biocatalyst for the formation of macrocyclic peptides.

## Conclusions

In summary, using a hypothesis-driven, phylogeny-guided approach, we identified ScaB as a highly promiscuous P450 macrocyclase capable of modifying a broad range of natural and non-natural atropopeptide precursors. ScaB efficiently installs either a C–N biaryl crosslink between two tryptophans or a C–C biaryl crosslink between tryptophan and tyrosine, enabling the generation of diverse cyclic peptides. Notably, three cyclic peptides with biaryl crosslink showed moderate antiviral activities and one of them exhibited anti-inflammatory properties. These findings highlight ScaB's potential as a versatile biocatalyst for the discovery and development of bioactive macrocyclic peptides with possible therapeutic applications.

## Author contributions

BT and EJNH planned the project and all experiments. BT designed and constructed plasmids, conducted the combinatorial biosynthesis and site-directed mutagenesis studies, isolated compounds and performed antibacterial assays. PB, LA and JH designed and performed anti-inflammatory assays. KH designed and conducted antiviral assays. BT and YK analysed NMR data. BT and FB constructed and analysed the phylogenetic tree. BT and EJNH wrote manuscript with contributions from all co-authors.

## Conflicts of interest

There are no conflicts to declare.

## Supplementary Material

SC-OLF-D5SC03525B-s001

SC-OLF-D5SC03525B-s002

## Data Availability

The data supporting this article have been included in the SI. The materials and methods section, all supplementary figures and tables, and the phylogenetic tree in Newick format are available in the supplementary information. See DOI: https://doi.org/10.1039/d5sc03525b.
