# Supplementary material for: Phylogeny-guided discovery of a promiscuous P450 macrocyclase for the production of diverse atropopeptides
Source: Chem Sci. 2025 Aug 6;16(35):16240–9. doi: 10.1039/d5sc03525b (PMC12352619; doi:10.1039/d5sc03525b)
Supplement: SC-016-D5SC03525B-s002 [file SC-016-D5SC03525B-s002.pdf]

Supplementary Information (SI) for Chemical Science.
This journal is © The Royal Society of Chemistry 2025

Supplementary information

Phylogeny-Guided Discovery of a Promiscuous P450 Macrocyclase for the production of diverse atropopeptides

Bin Tan,[a] Peter Breunig,[b] Lamia Arbib,[b] Yuya Kakumu,[a] Friederike Biermann,[a] Kornelia Hardes,[c] Jasmin Hefendehl,[b] Eric J. N. Helfrich*[a, d]

[a] Dr. B. Tan, Dr. Y. Kakumu, F. Biermann, Prof. Dr. E. J. N. Helfrich
Institute for Molecular Bio Science, Goethe University Frankfurt
Max-von-Laue Strasse 9, 60438 Frankfurt am Main, Germany

[b] Dr. P. Breunig, L. Arbib, Prof. Dr. J. Hefendehl
Institute of Cell Biology and Neuroscience, Goethe University Frankfurt and Buchmann Institute for Molecular Life Sciences
Max-von-Laue Strasse 9, 60438 Frankfurt am Main, Germany

[c] Prof. Dr. K. Hardes
Department of Bioresources, Fraunhofer Institute for Molecular Biology and Applied Ecology
Ohlebergsweg 12, 35392 Giessen, Germany

[d] Prof. Dr. E. J. N. Helfrich
Senckenberg Gesellschaft für Naturforschung
Senckenberganlage 25, 60325 Frankfurt, Germany

*corresponding author: Eric J.N. Helfrich (E-mail: eric.helfrich@bio.uni-frankfurt.de)

Contents

Figure S1. Phylogenetic tree of atropopeptide-modifying P450s. S4
Figure S2. Combinatorial biosynthesis of atropopeptide precursors with ScaB. S5
Figure S3. HRMS analysis of extracts of the recombinant *S. albus* harboring *lucA* and *scaB* S6
Figure S4. HRMS analysis of extracts of the recombinant *S. albus* harboring *capA* and *scaB* S7
Figure S5. HRMS analysis of extracts of the recombinant *S. albus* harboring *nouA* and *scaB* S8
Figure S6. HRMS analysis of extracts of the recombinant *S. albus* harboring *katA* and *scaB* S9
Figure S7. HRMS analysis of extracts of the recombinant *S. albus* harboring *jumA* and *scaB* S10
Figure S8. HRMS analysis of extracts of the recombinant *S. albus* harboring *xatA* and *scaB* S11
Figure S9. HRMS analysis of extracts of the recombinant *S. albus* harboring *xavA* and *scaB* S12
Figure S10. HRMS analysis of extracts of the recombinant *S. albus* harboring *xamA2* and *scaB* S13
Figure S11. HRMS analysis of extracts of the recombinant *S. albus* harboring *xamA1* and *scaB* S14
Figure S12. HRMS analysis of extracts of the recombinant *S. albus* harboring *xaaA* and *scaB* S15
Figure S13. HRMS analysis of extracts of the recombinant *S. albus* harboring *svaA* and *scaB* S16
Figure S14. HRMS analysis of extracts of the recombinant *S. albus* harboring *skaA* and *scaB* S17
Figure S15. HRMS analysis of extracts of the recombinant *S. albus* harboring *lauA* and *scaB* S18
Figure S16. Co-expression of truncated *scaA* variants with *scaB*.. .... S19
Figure S17. HPLC-ESI-QTOF-HRMS analysis of scabrirubin CB-1 (**1**). S20
Figure S18. ^{1}H NMR spectrum (600 MHz) of scabrirubin CB-1 (**1**) in DMSO-d_6. .... S21
Figure S19. ^{13}C NMR spectrum (150 MHz) of scabrirubin CB-1 (**1**) in DMSO-d_6. .... S22
Figure S20. COSY spectrum (600 MHz) of scabrirubin CB-1 (**1**) in DMSO-d_6. .... S23
Figure S21. HSQC spectrum (600 MHz) of scabrirubin CB-1 (**1**) in DMSO-d_6. .... S24
Figure S22. HMBC spectrum (600 MHz) of scabrirubin CB-1 (**1**) in DMSO-d_6. .... S25
Figure S23. NOESY spectrum (600 MHz) of scabrirubin CB-1 (**1**) in DMSO-d_6. .... S26
Figure S24. HPLC-ESI-QTOF-HRMS analysis of scabrirubin CB-2 (**2**). S27
Figure S25. ^{1}H NMR spectrum (600 MHz) of scabrirubin CB-2 (**2**) in DMSO-d_6 .... S28
Figure S26. ^{13}C NMR spectrum (125 MHz) of scabrirubin CB-2 (**2**) in DMSO-d_6. .... S29
Figure S27. COSY spectrum (500 MHz) of scabrirubin CB-2 (**2**) in DMSO-d_6. .... S30
Figure S28. HSQC spectrum (500 MHz) of scabrirubin CB-2 (**2**) in DMSO-d_6. .... S31
Figure S29. HMBC spectrum (500 MHz) of scabrirubin CB-2 (**2**) in DMSO-d_6. .... S32
Figure S30. NOESY spectrum (500 MHz) of scabrirubin CB-2 (**2**) in DMSO-d_6. .... S33
Figure S31. HPLC-ESI-QTOF-HRMS analysis of scabrirubin CB-3 (**3**). S34
Figure S32. ^{1}H NMR spectrum (500 MHz) of scabrirubin CB-3 (**3**) in DMSO-d_6. .... S35
Figure S33. ^{13}C NMR spectrum (125 MHz) of scabrirubin CB-3 (**3**) in DMSO-d_6. .... S36
Figure S34. COSY spectrum (500 MHz) of scabrirubin CB-3 (**3**) in DMSO-d_6. .... S37
Figure S35. HSQC spectrum (500 MHz) of scabrirubin CB-3 (**3**) in DMSO-d_6. .... S38
Figure S36. HMBC spectrum (500 MHz) of scabrirubin CB-3 (**3**) in DMSO-d_6. .... S39
Figure S37. NOESY spectrum (500 MHz) of scabrirubin CB-3 (**3**) in DMSO-d_6. .... S40
Figure S38. HPLC-ESI-QTOF-HRMS analysis of scabrirubin CB-4 (**4**). S41
Figure S39. ^{1}H NMR spectrum (500 MHz) of scabrirubin CB-4 (**4**) in DMSO-d_6. .... S42
Figure S40. ^{13}C NMR spectrum (125 MHz) of scabrirubin CB-4 (**4**) in DMSO-d_6. .... S43
Figure S41. COSY spectrum (500 MHz) of scabrirubin CB-4 (**4**) in DMSO-d_6. .... S44
Figure S42. HSQC spectrum (500 MHz) of scabrirubin CB-4 (**4**) in DMSO-d_6. .... S45
Figure S43. HMBC spectrum (500 MHz) of scabrirubin CB-4 (**4**) in DMSO-d_6. .... S46
Figure S44. NOESY spectrum (500 MHz) of scabrirubin CB-4 (**4**) in DMSO-d_6. .... S47
Figure S45. HRMS analysis of extracts of the recombinant *S. albus* harboring *scaA*-F24A and *scaB* S48
Figure S46. HRMS analysis of extracts of the recombinant *S. albus* harboring *scaA*-F24E and *scaB*. S49
Figure S47. HRMS analysis of extracts of the recombinant *S. albus* harboring *scaA*-F24H and *scaB* S50
Figure S48. HRMS analysis of extracts of the recombinant *S. albus* harboring *scaA*-F24L and *scaB* S51
Figure S49. HRMS analysis of extracts of the recombinant *S. albus* harboring *scaA*-F24N and *scaB* S52
Figure S50. HRMS analysis of extracts of the recombinant *S. albus* harboring *scaA*-F24P and *scaB* S53
Figure S51. HRMS analysis of extracts of the recombinant *S. albus* harboring *scaA*-F24Q and *scaB*. S54
Figure S52. HRMS analysis of extracts of the recombinant *S. albus* harboring *scaA*-F24R and *scaB*. S55
Figure S53. HRMS analysis of extracts of the recombinant *S. albus* harboring *scaA*-F24T and *scaB* S56
Figure S54. HRMS analysis of extracts of the recombinant *S. albus* harboring *scaA*-I25A and *scaB* S57
Figure S55. HRMS analysis of extracts of the recombinant *S. albus* harboring *scaA*-I25E and *scaB* S58
Figure S56. HRMS analysis of extracts of the recombinant *S. albus* harboring *scaA*-I25H and *scaB*. S59
Figure S57. HRMS analysis of extracts of the recombinant *S. albus* harboring *scaA*-I25L and *scaB* S60

Figure S58. HRMS analysis of extracts of the recombinant *S. albus* harboring *scaA*-I25N and *scaB* S61
Figure S59. HRMS analysis of extracts of the recombinant *S. albus* harboring *scaA*-I25P and *scaB*............... S62
Figure S60. HRMS analysis of extracts of the recombinant *S. albus* harboring *scaA*-I25Q and *scaB*. S63
Figure S61. HRMS analysis of extracts of the recombinant *S. albus* harboring *scaA*-I25T and *scaB*............... S64
Figure S62. HRMS analysis of extracts of the recombinant *S. albus* harboring *scaA*-I25Y and *scaB*............... S65
Figure S63. Effect of crosslink-forming residue substitutions in XamA2, XaaA and ScaA precursors. S66
Figure S64. HRMS analysis of extracts of the recombinant *S. albus* harboring *xamA2*-Y25W and *scaB*.. .... S67
Figure S65. HRMS analysis of extracts of the recombinant *S. albus* harboring *xaaA*-Y26W and *scaB*. S68
Figure S66. Gating strategy for flow cytometry analysis of bEnd.3 cell inflammation assay. S69
Figure S67. Effect of atropopeptides on the viability of MDCK II cells. S70
Figure S68. The predicted structure models of ScaB, LauB1 and SvaB and their precursor peptides S71
Figure S69.. Representative macrocyclices catalyzed by P450s from RiPP biosynthesis. S72
Table S1. Calculated mass for linear core peptide and monocyclic peptide and observed mass for product..... S73
Table S2. NMR data of scabrirubin CB-1 (**1**)... S74
Table S3. NMR data of scabrirubin CB-2 (**2**)... S75
Table S4. NMR data of scabrirubin CB-3 (**3**)... S76
Table S5. NMR data of scabrirubin CB-4 (**4**)... S77
Table S6 Strains and plasmids used in this study S78
Table S7. Primers used in this study S82
Materials and Methods S86
General materials S86
Strains and culture conditions S86
DNA isolation, manipulation and sequencing S86
General bioinformatics analysis S86
Construction of recombinant plasmids S87
Heterologous expression of recombinant BGCs S87
Small-scale fermentation and LC-MS analysis S88
Large-scale fermentation and purification of compounds S88
Physical and spectroscopic data of isolated compounds S89
NMR analysis S89
Structure elucidation S89
Antimicrobial assay S91
Anti-inflammatory assays S91
Antiviral assays S92
DNA sequences S92
References: S96

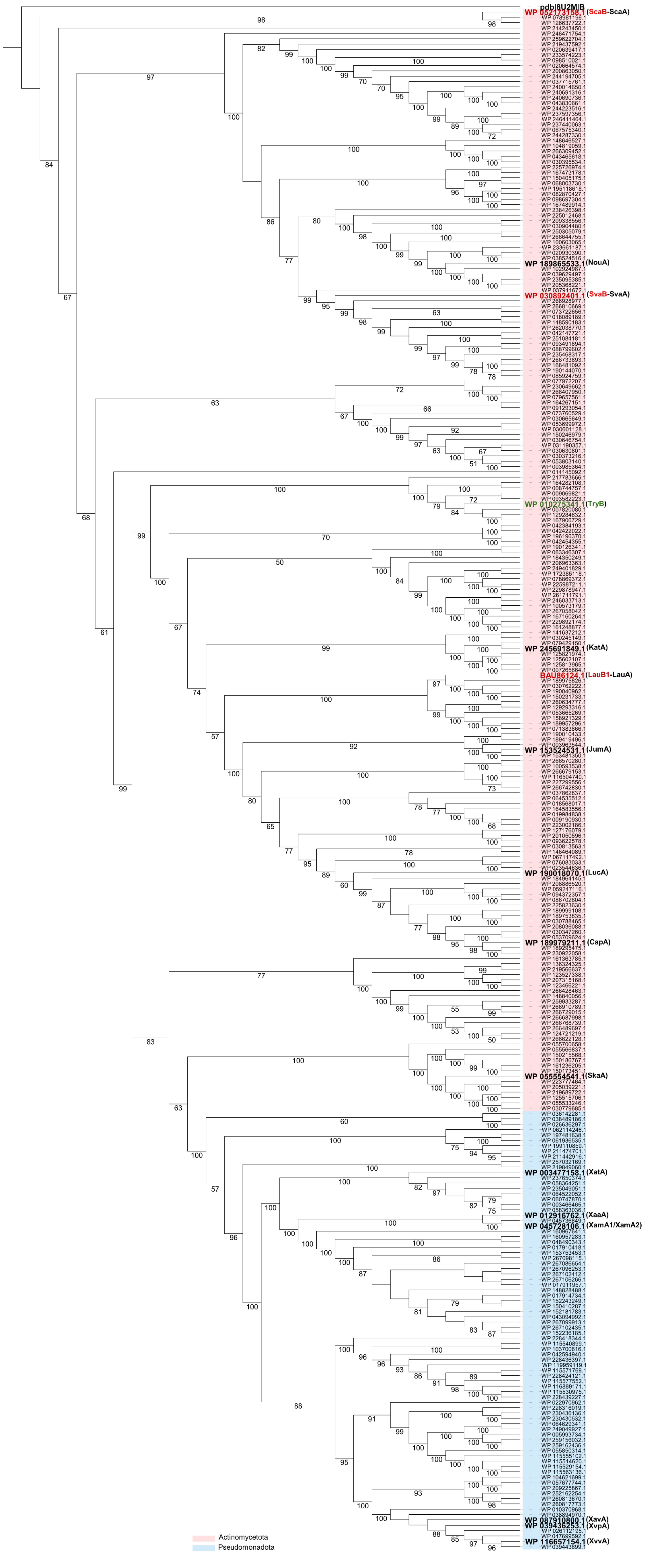

Figure S1. Phylogenetic tree of atropopeptide-modifying P450s. The maximum likelihood tree was rooted using P450Blt 8U2M_B as outgroup.[1] Bootstrap values are based on 1000 bootstrap replicates. The P450s ScaB, SvaB and LauB1 used for combinatorial biosynthesis are highlighted in red and the P450 TryB involved in tryptorubin biosynthesis is highlighted in green. The 15 selected atropopeptide precursors are labelled in the position of their corresponding P450.

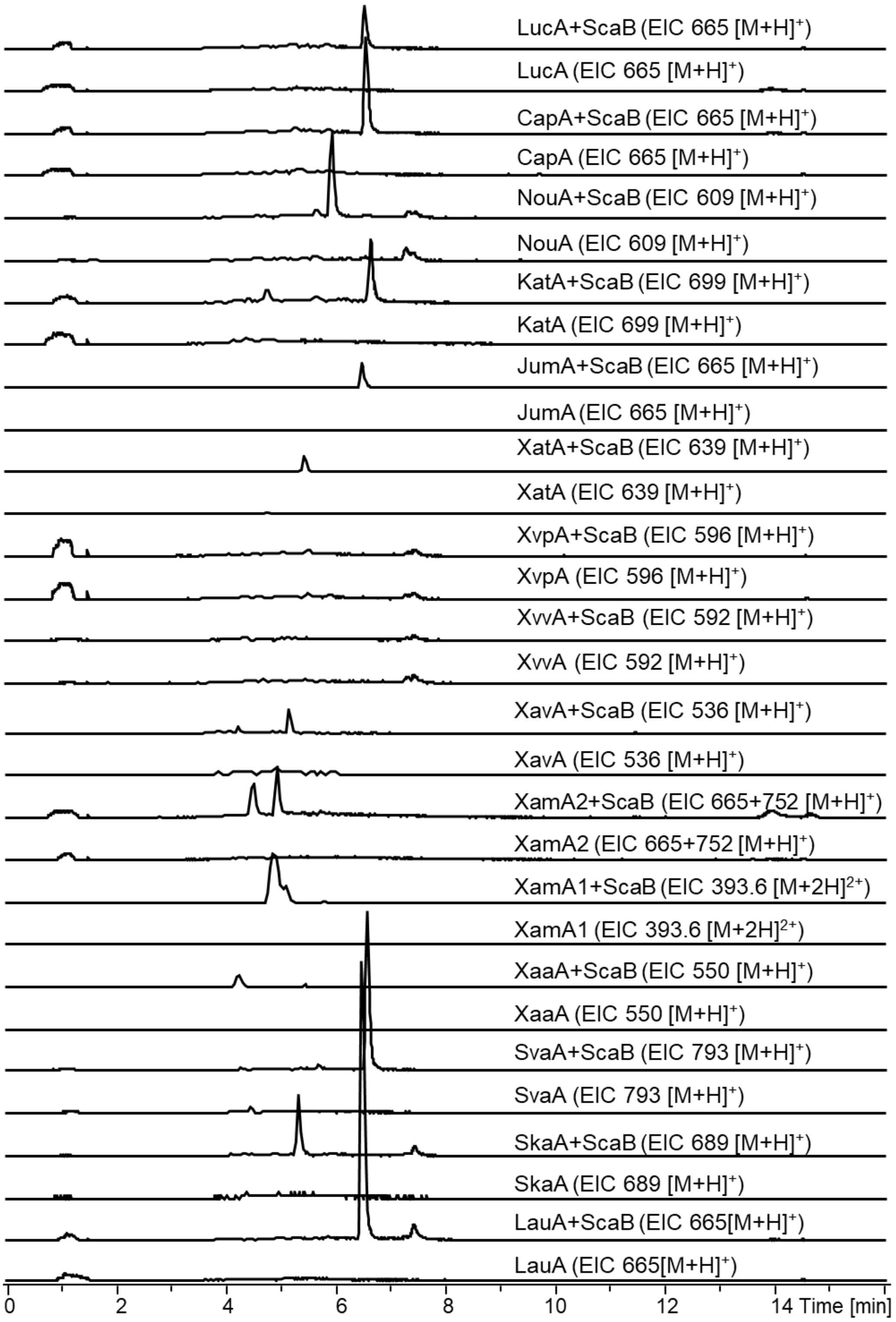

Figure S2. Combinatorial biosynthesis of atropopeptide precursors with ScaB. Extracted ion chromatograms of cyclic peptides generated from the coexpression of different atropopeptide precursors with ScaB and their corresponding controls.

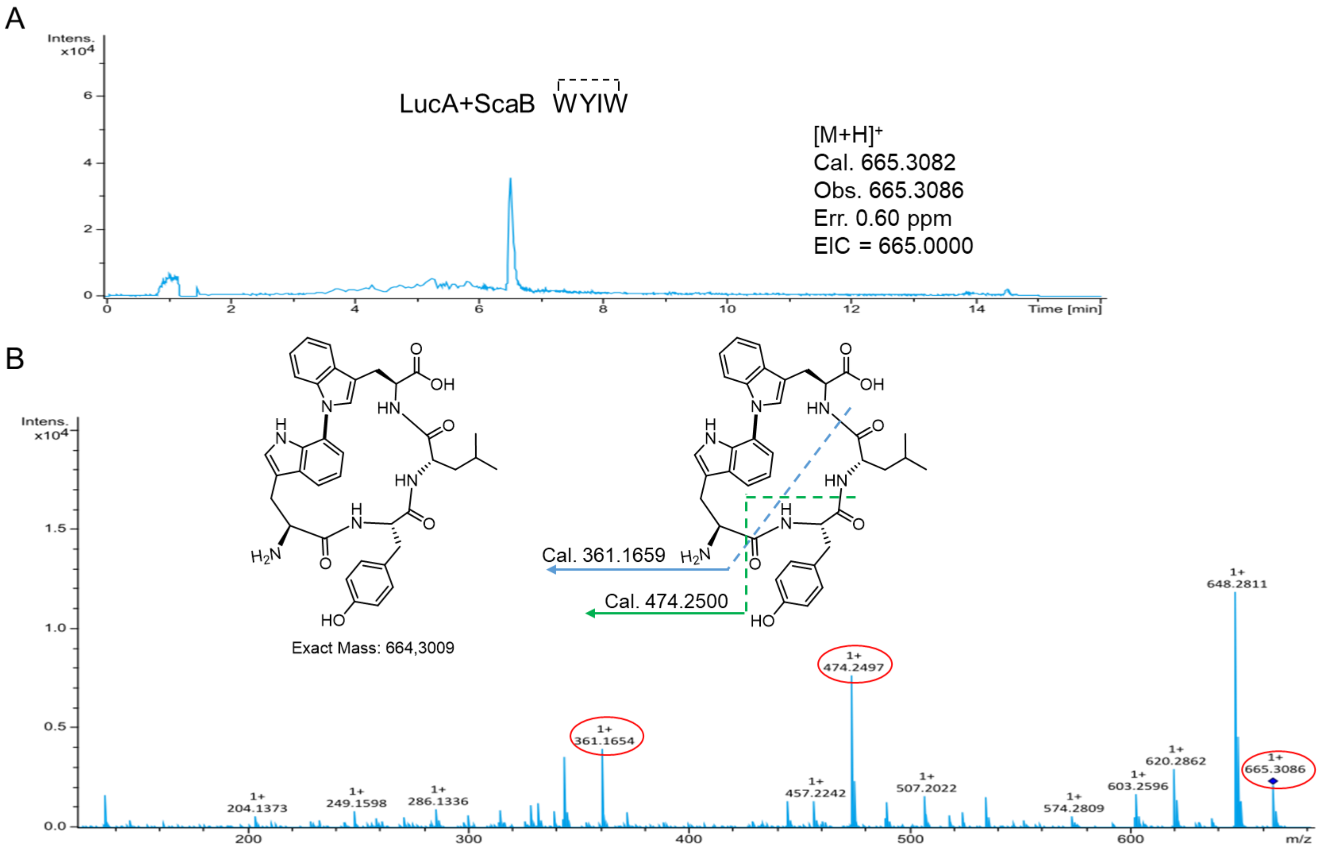

Figure S3. HRMS analysis of extracts of the recombinant *S. albus* strain harboring *lucA* and *scaB.* A) Extracted ion chromatogram of the product from the coexpression of *lucA* with *scaB*; B) MS/MS spectrum of the corresponding atropopeptide detected at *m*/*z* 665.3086 $[M+H]^+$ with key fragments that indicate the presence of a bond between the two Trp residues highlighted.

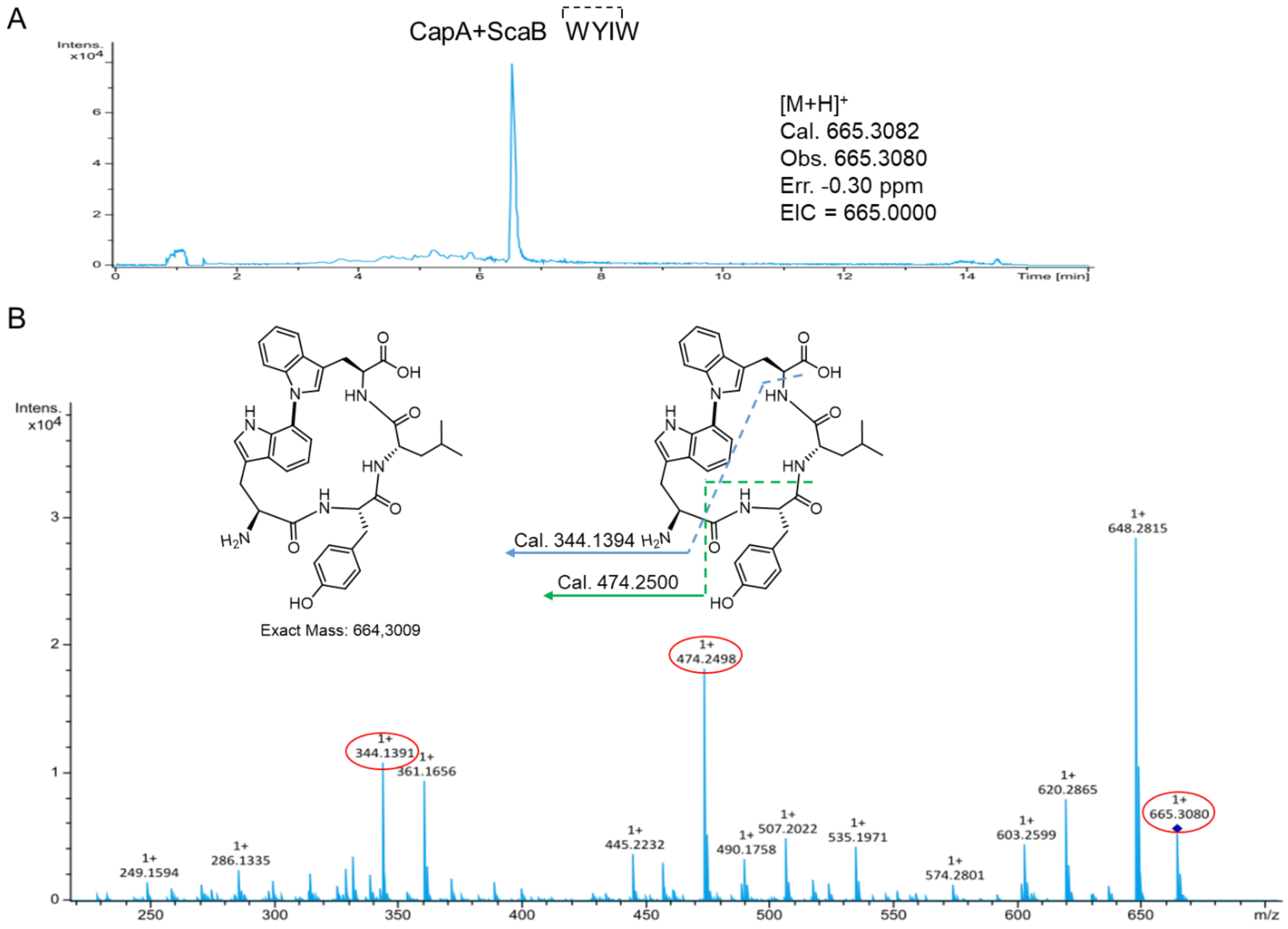

Figure S4. HRMS analysis of extracts of the recombinant *S. albus* strain harboring *capA* and *scaB*. A) Extracted ion chromatogram of the product from the coexpression of *capA* with *scaB*; B) MS/MS spectrum of the corresponding atropopeptide detected at *m*/*z* 665.3080 $[M+H]^+$ with key fragments that indicate the presence of a bond between the two Trp residues highlighted.

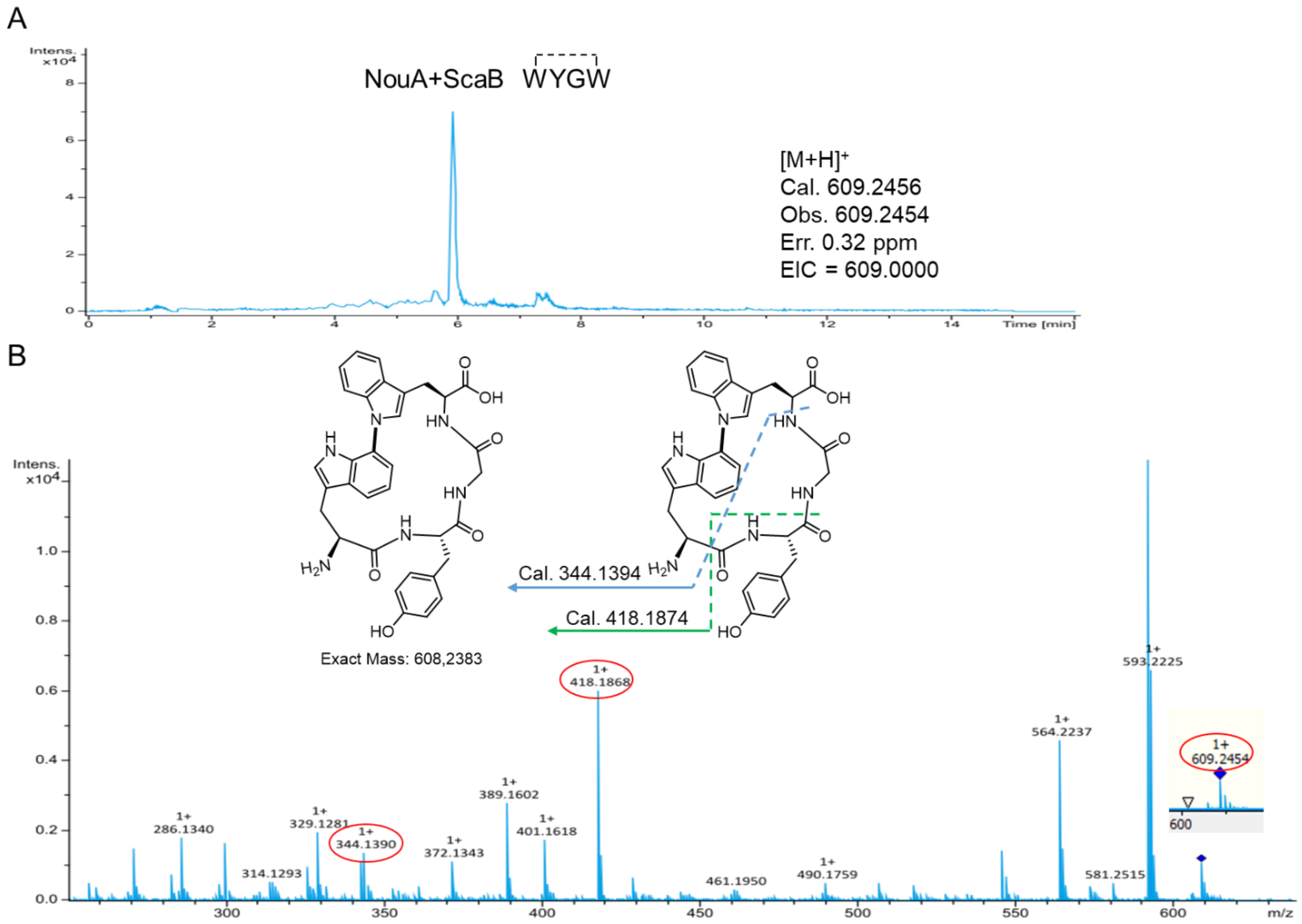

Figure S5. HRMS analysis of extracts of the recombinant *S. albus* strain harboring *nouA* and *scaB*. A) Extracted ion chromatogram of the product from the coexpression of *nouA* with *scaB*; B) MS/MS spectrum of the corresponding atropopeptide detected at *m*/*z* 609.2454 $[M+H]^+$ with key fragments that indicate the presence of a bond between the two Trp residues highlighted. The parent ion *m*/*z* 609.2454 $[M+H]^+$ can be observed in the magnified inserted region.

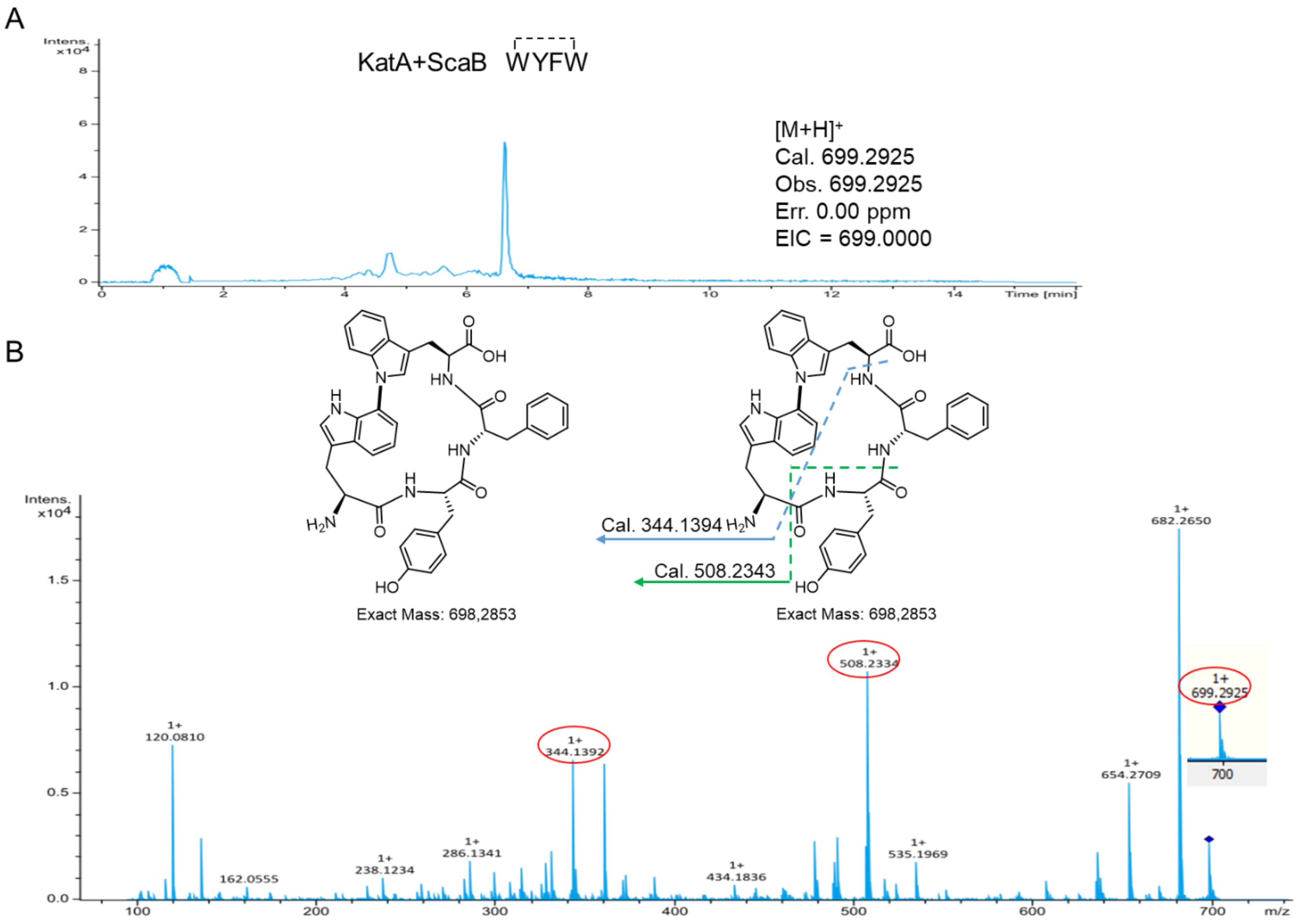

Figure S6. HRMS analysis of extracts of the recombinant *S. albus* strain harboring *katA* and *scaB*. A) Extracted ion chromatogram of the product from the coexpression of *katA* with *scaB*; B) MS/MS spectrum of the corresponding atropopeptide detected at *m*/*z* 699.2925 $[M+H]^+$. The parent ion *m*/*z* 699.2925 $[M+H]^+$ can be observed in the magnified inserted region.

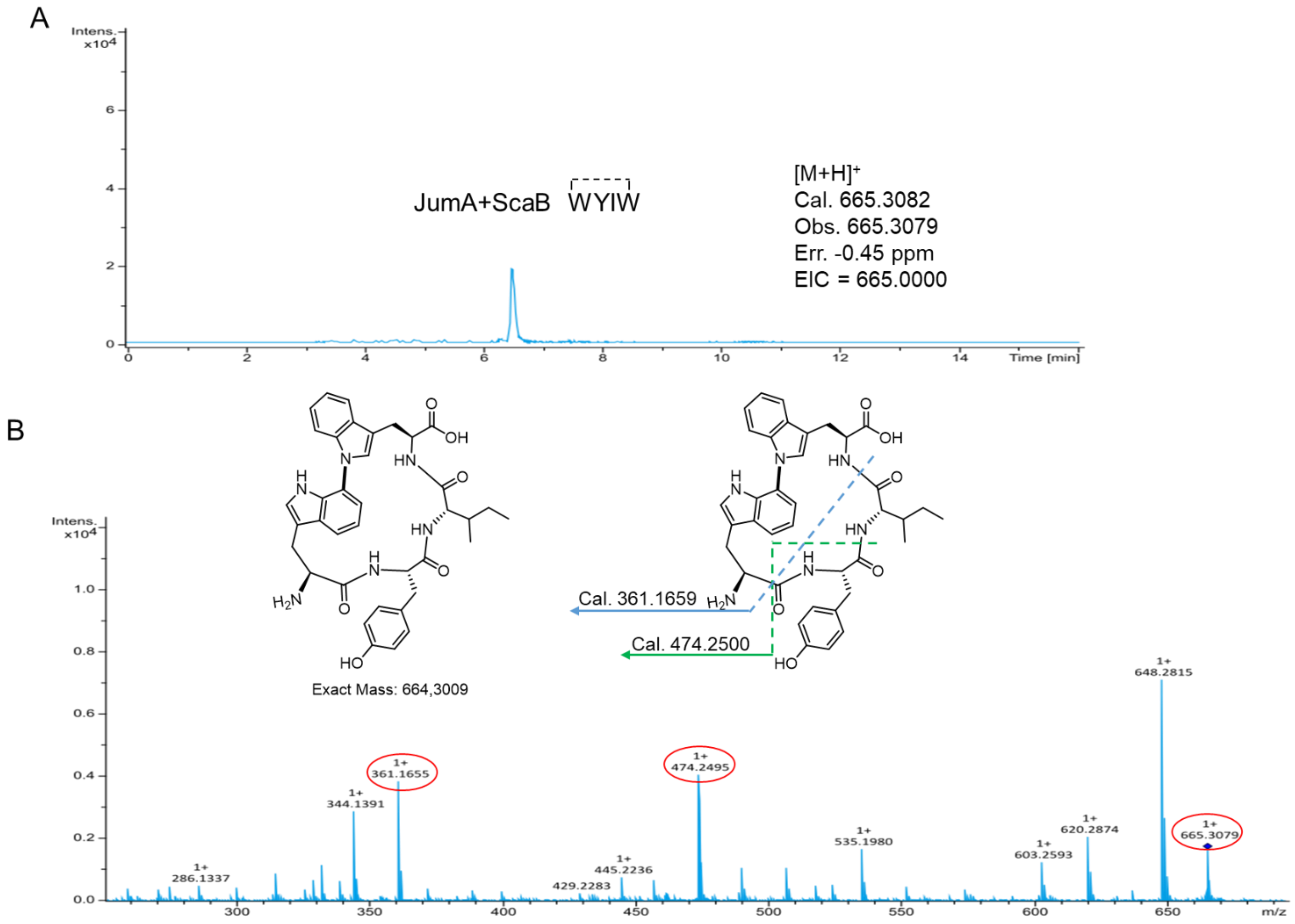

Figure S7. HRMS analysis of extracts of the recombinant *S. albus* strain harboring *jumA* and *scaB*. A) Extracted ion chromatogram of the product from the coexpression of *jumA* with *scaB*; B) MS/MS spectrum of the corresponding atropopeptide detected at *m*/*z* 665.3079 $[M+H]^+$ with key fragments that indicate the presence of a bond between the two Trp residues highlighted.

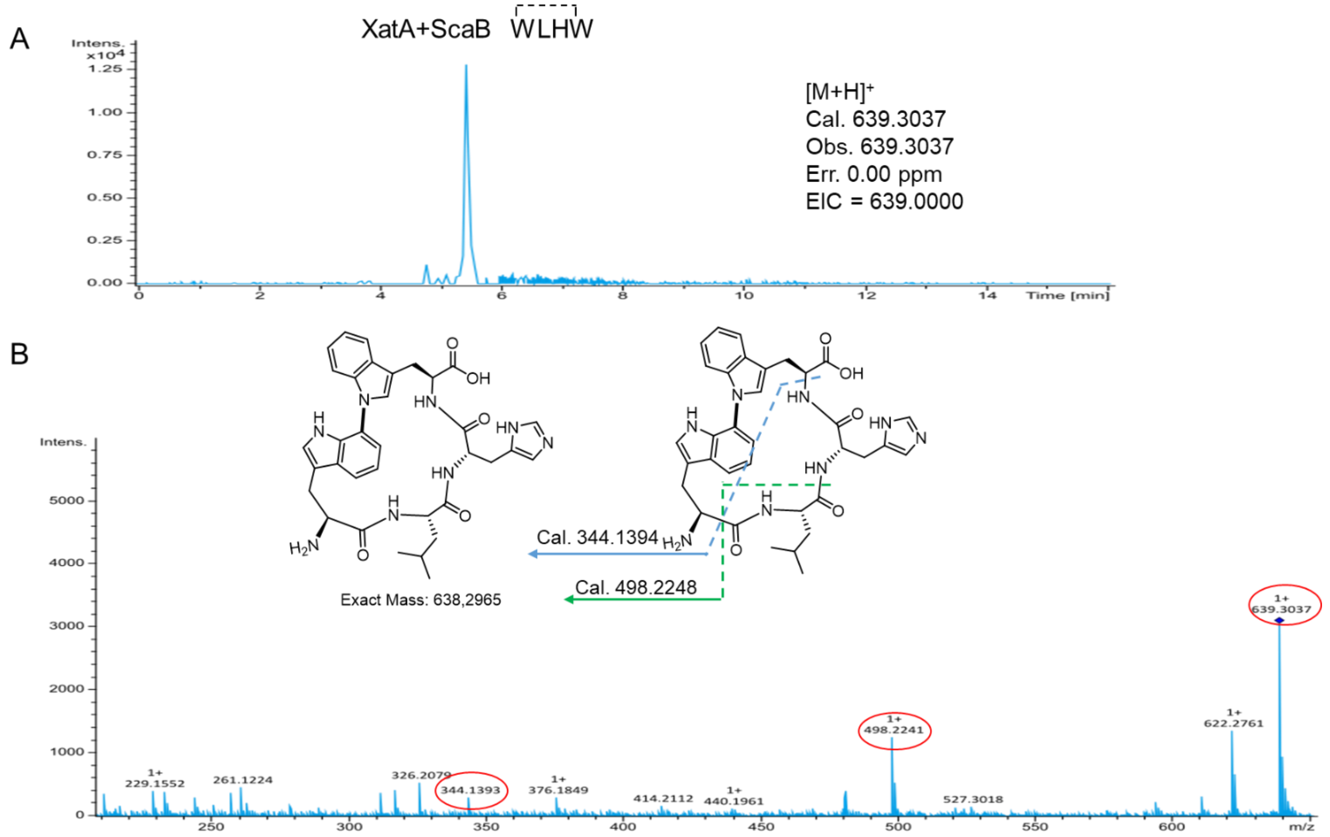

Figure S8. HRMS analysis of extracts of the recombinant *S. albus* strain harboring *xatA* and *scaB*. A) Extracted ion chromatogram of the product from the coexpression of *xatA* with *scaB*; B) MS/MS spectrum of the corresponding atropopeptide detected at *m*/*z* 639.3037 $[M+H]^+$ with key fragments that indicate the presence of a bond between the two Trp residues highlighted.

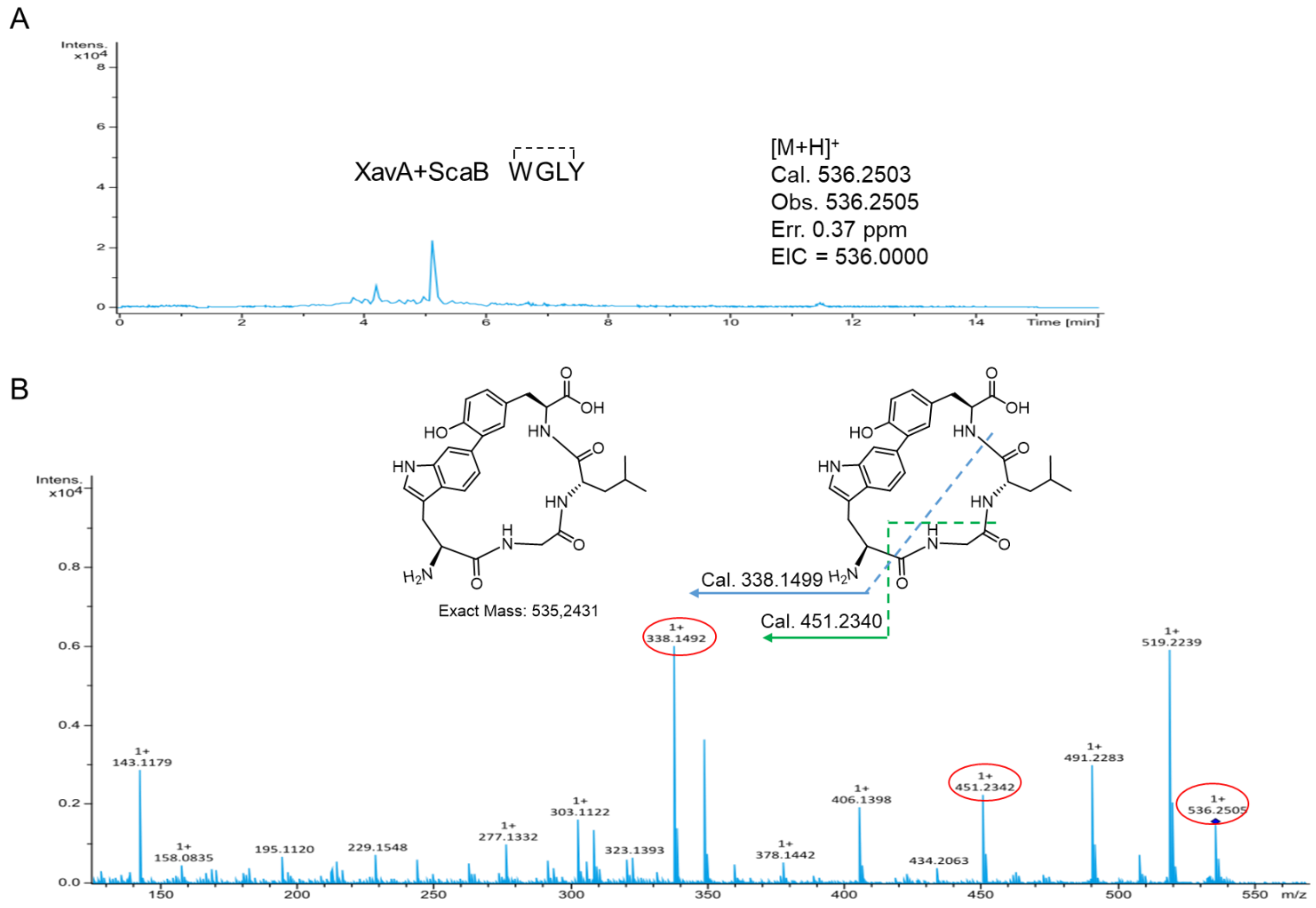

Figure S9. HRMS analysis of extracts of the recombinant *S. albus* strain harboring *xavA* and *scaB*. A) Extracted ion chromatogram of the product from the coexpression of *xavA* with *scaB*; B) MS/MS spectrum of the corresponding atropopeptide detected at *m*/*z* 536.2505 $[M+H]^+$ with key fragments that indicate the presence of a bond between Trp and Tyr highlighted.

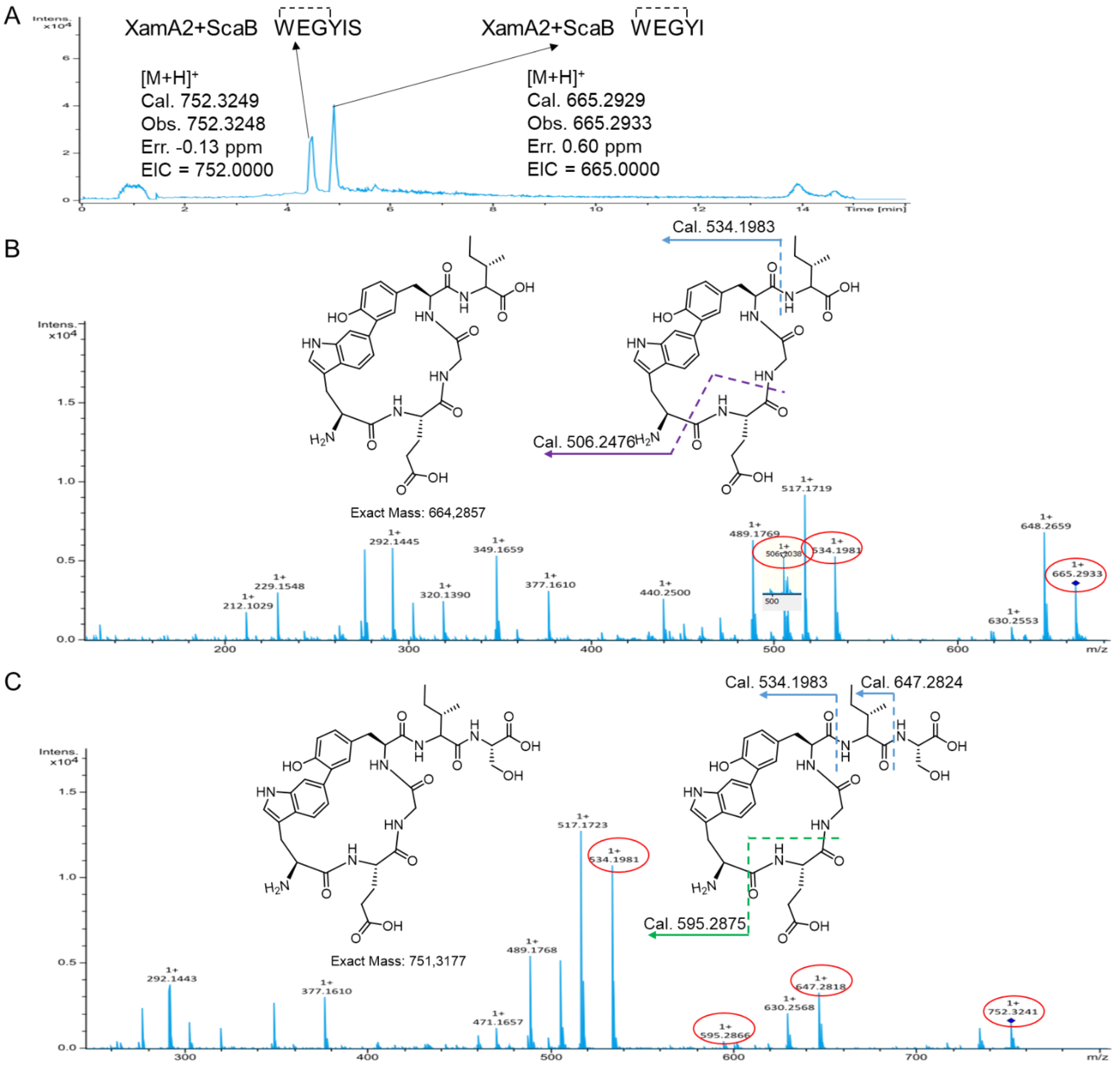

Figure S10. HRMS analysis of extracts of the recombinant *S. albus* strain harboring *xamA2* and *scaB*. A) Extracted ion chromatogram of the product from the coexpression of *xamA2* with *scaB*; B) MS/MS spectrum of the corresponding atropopeptide detected at *m*/*z* 665.2933 [M+H]$^+$. The fragment with *m*/*z* 506 [M+H]$^+$ can be observed in the magnified inserted region; C) MS/MS spectrum of the corresponding atropopeptide detected at *m*/*z* 752.3248 [M+H]$^+$ with key fragments that indicate the presence of a bond between Trp and Tyr highlighted.

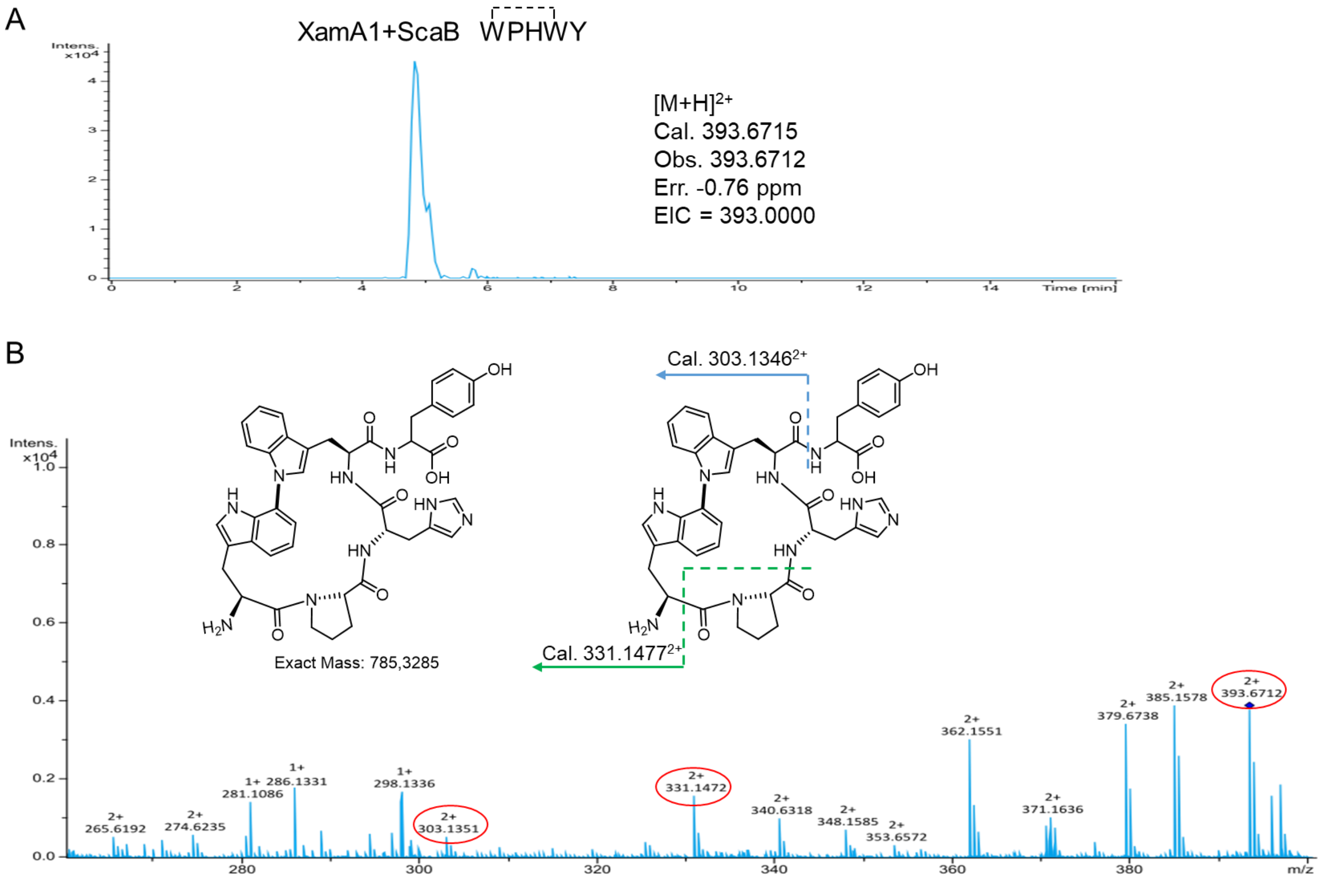

Figure S11. HRMS analysis of extracts of the recombinant *S. albus* harboring *xamA1* and *scaB*. A) Extracted ion chromatogram of the product from the coexpression of *xamA1* with *scaB*; B) MS/MS spectrum of the corresponding atropopeptide detected at *m*/*z* 393.6712 $[M+H]^{2+}$ with key fragments that indicate the presence of a bond between the two Trp residues highlighted.

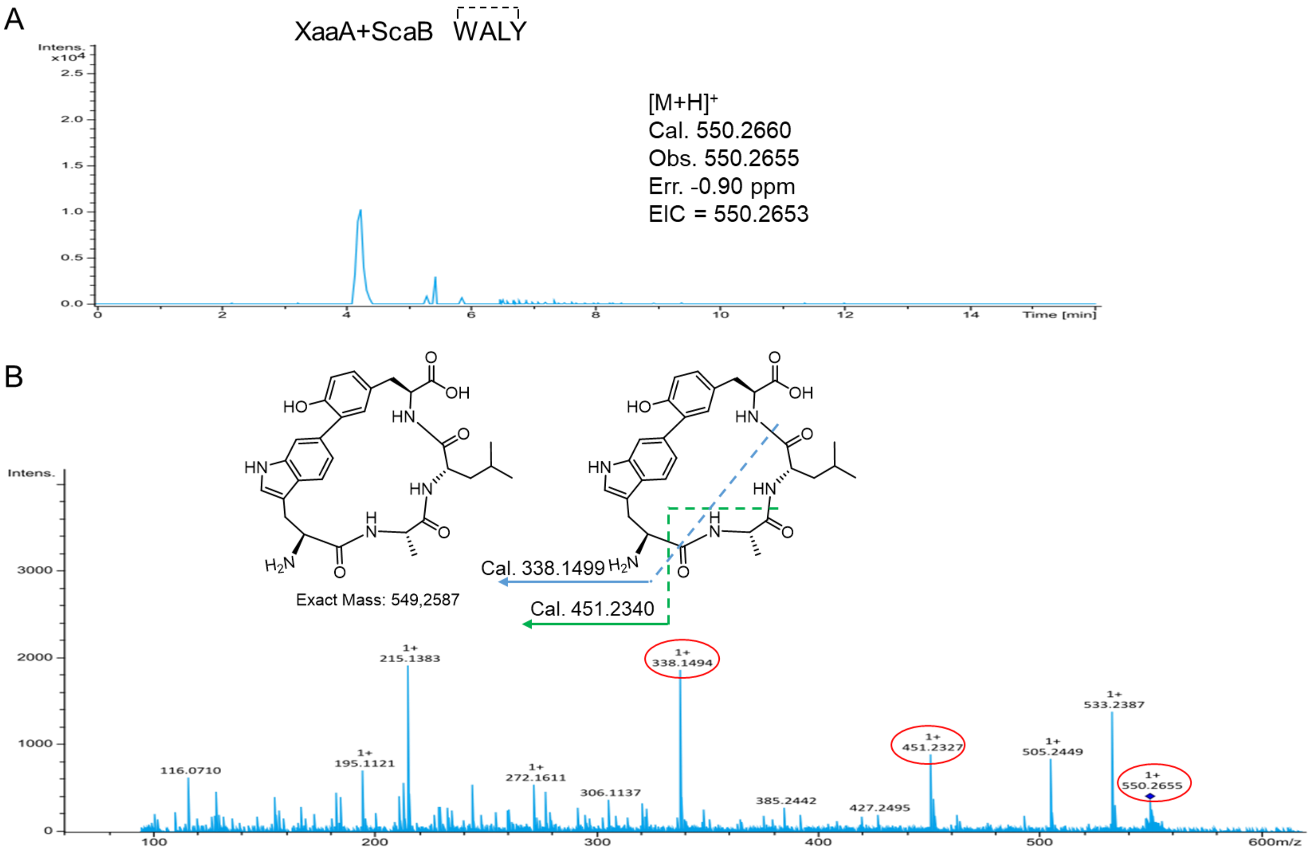

Figure S12. HRMS analysis of extracts of the recombinant *S. albus* strain harboring *xaaA* and *scaB*. A) Extracted ion chromatogram of the product from the coexpression of *xaaA* with *scaB*; B) MS/MS spectrum of the corresponding atropopeptide detected at *m*/*z* 550.2655 $[M+H]^+$ with key fragments that indicate the presence of a bond between Trp and Tyr highlighted.

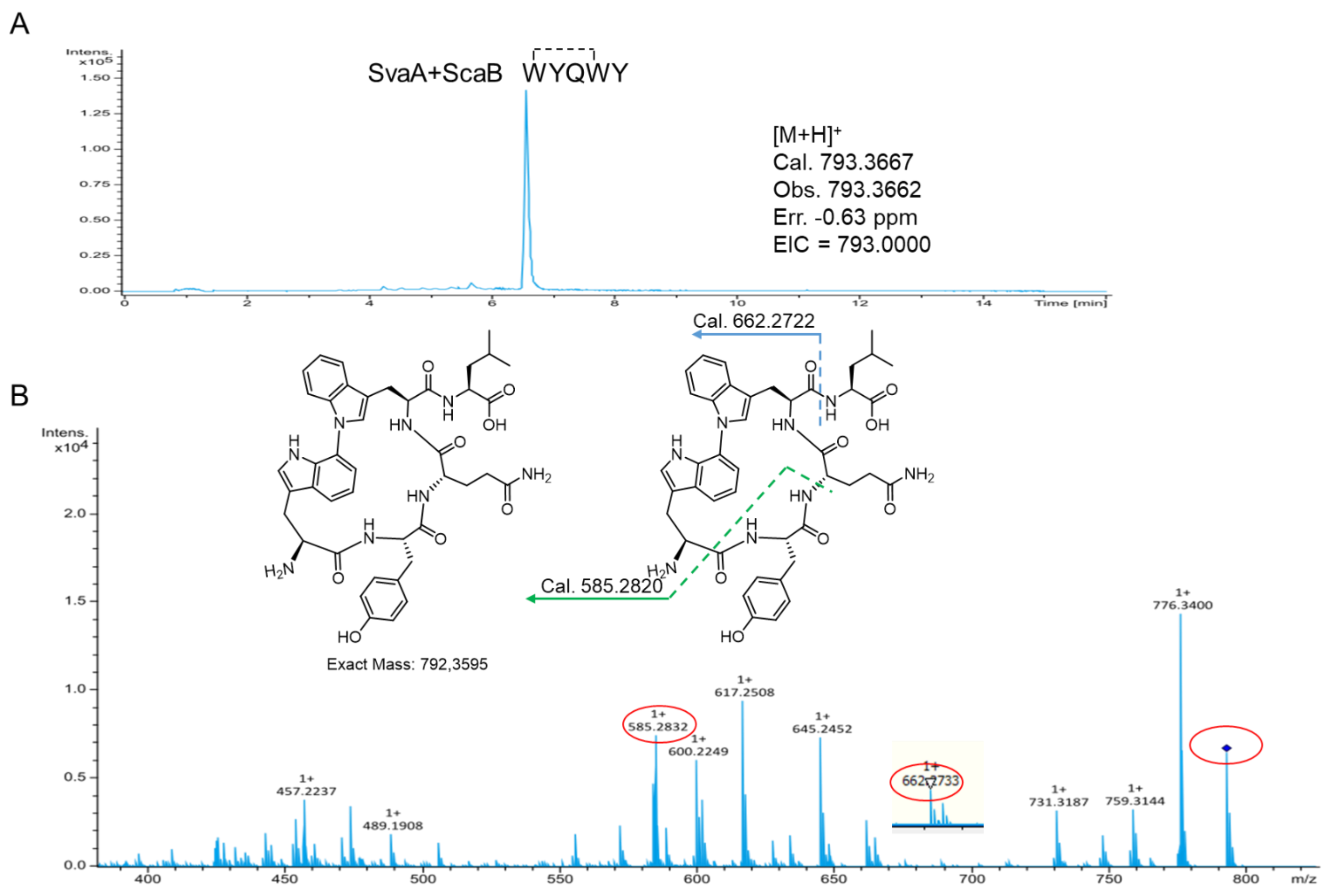

Figure S13. HRMS analysis of extracts of the recombinant *S. albus* strain harboring *svaA* and *scaB*. A) Extracted ion chromatogram of the product from the coexpression of *svaA* with *scaB*; B) MS/MS spectrum of the corresponding atropopeptide detected at *m*/*z* 793.3662 $[M+H]^+$ with key fragments that indicate the presence of a bond between the two Trp residues highlighted. The fragment with *m*/*z* 662 $[M+H]^+$ can be observed in the magnified inserted region.

A

B

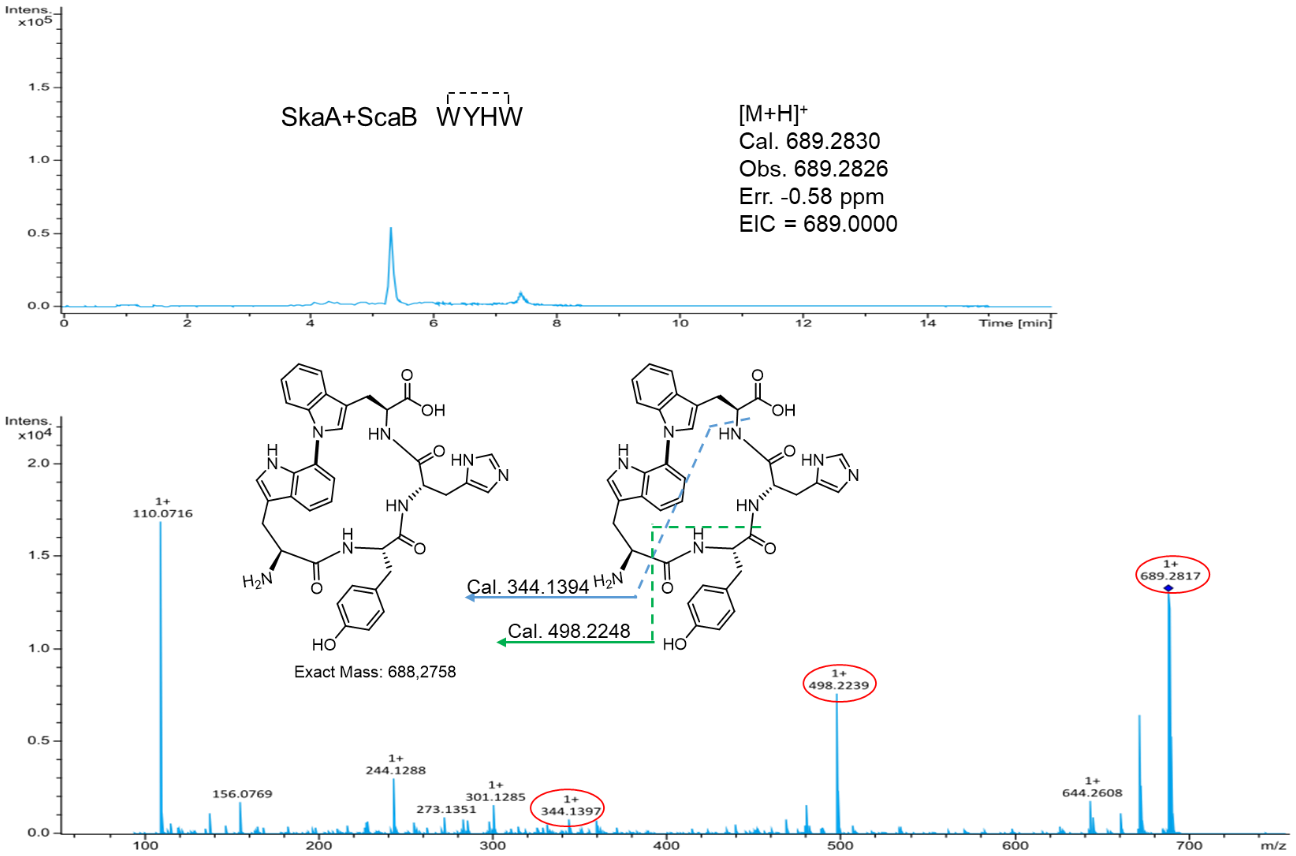

Figure S14. HRMS analysis of extracts of the recombinant *S. albus* strain harboring *skaA* and *scaB*. A) Extracted ion chromatogram of the product from the coexpression of *skaA* with *scaB*; B) MS/MS spectrum of the corresponding atropopeptide detected at *m*/*z* 689.2826 $[M+H]^+$ with key fragments that indicate the presence of a bond between the two Trp residues highlighted.

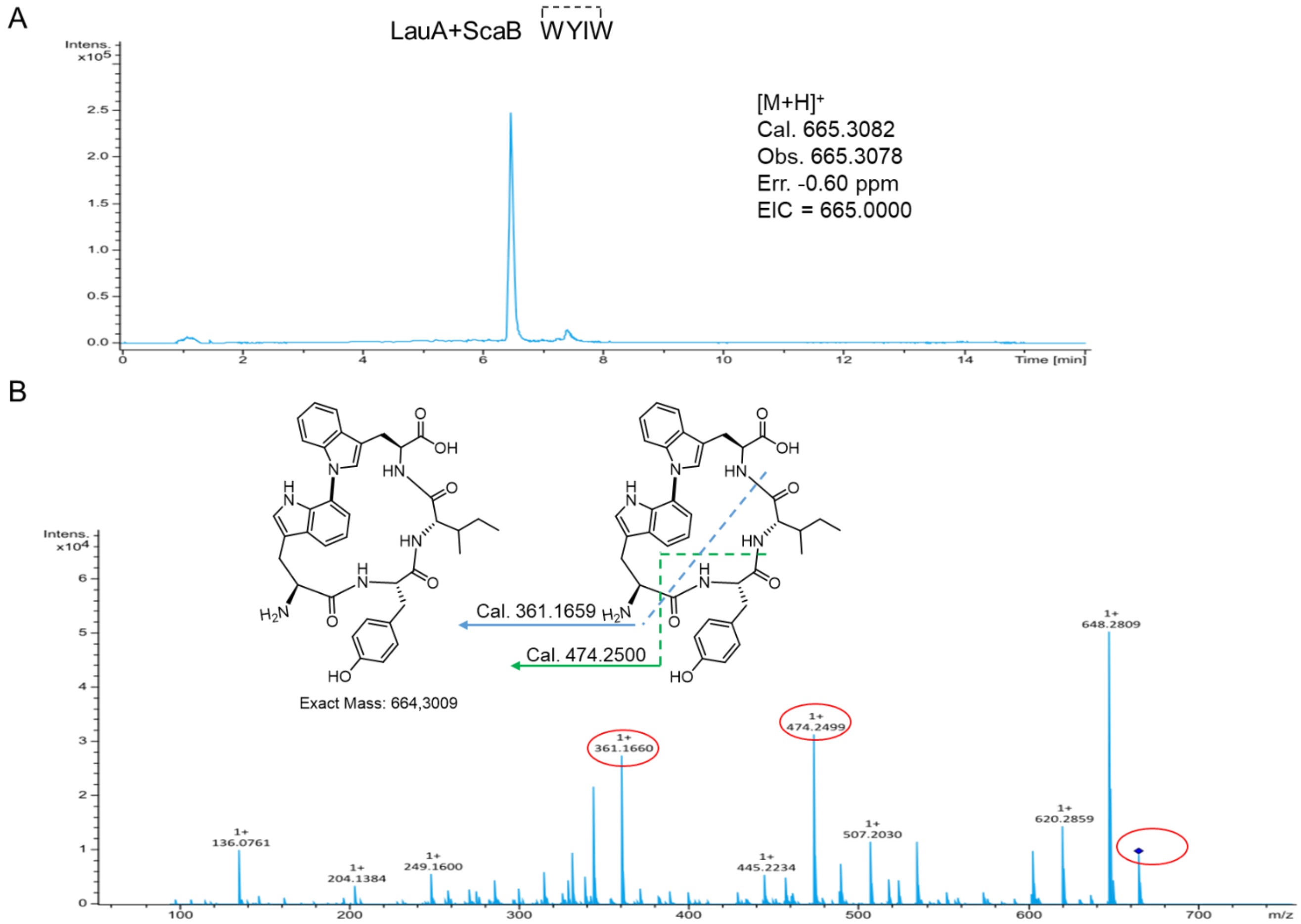

Figure S15. HRMS analysis of extracts of the recombinant *S. albus* strain harboring *lauA* and *scaB*. A) Extracted ion chromatogram of the product from the coexpression of *lauA* with *scaB*; B) MS/MS spectrum of the corresponding atropopeptide detected at *m*/*z* 665.3078 $[M+H]^+$ with key fragments that indicate the presence of a bond between the two Trp residues highlighted.

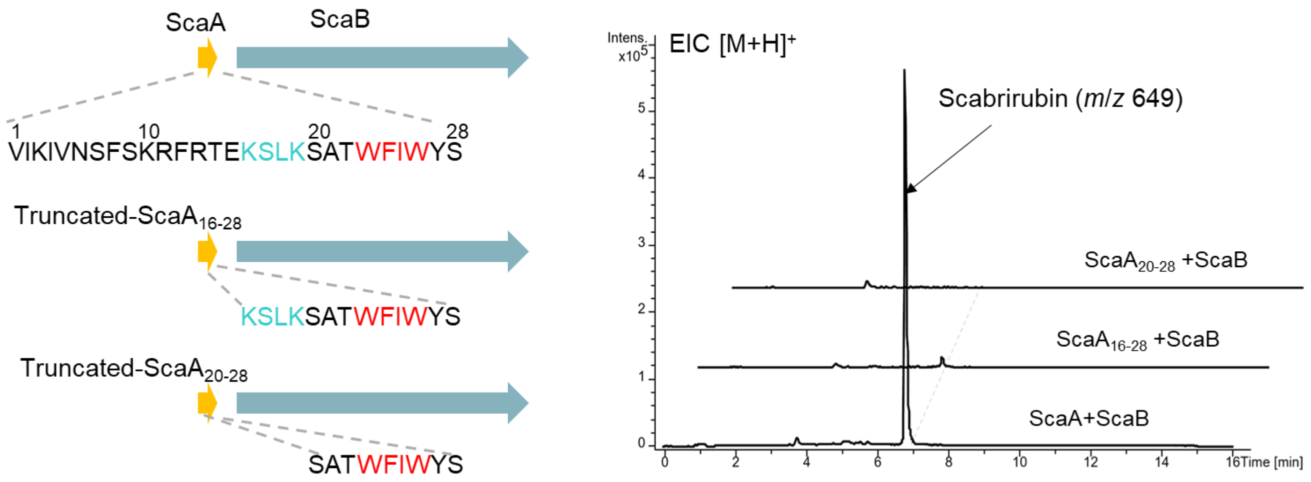

Figure S16. Co-expression of truncated *scaA* variants with *scaB*. The extracted ion chromatogram of the product generated from the co-expression of *scaA* or its variants with *scaB*.

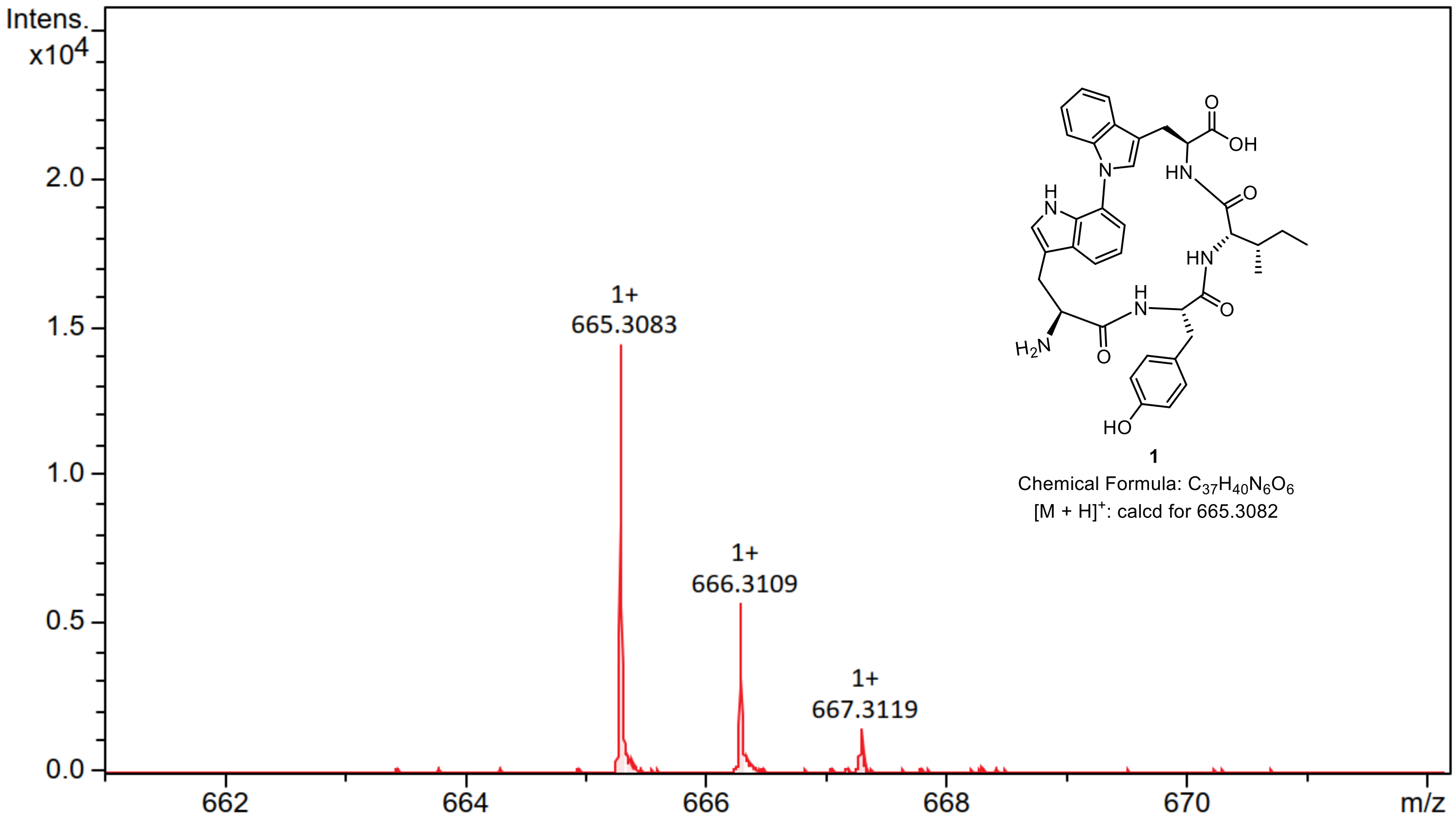

Figure S17. HPLC-ESI-QTOF-HRMS analysis of scabrirubin CB-1 (**1**).

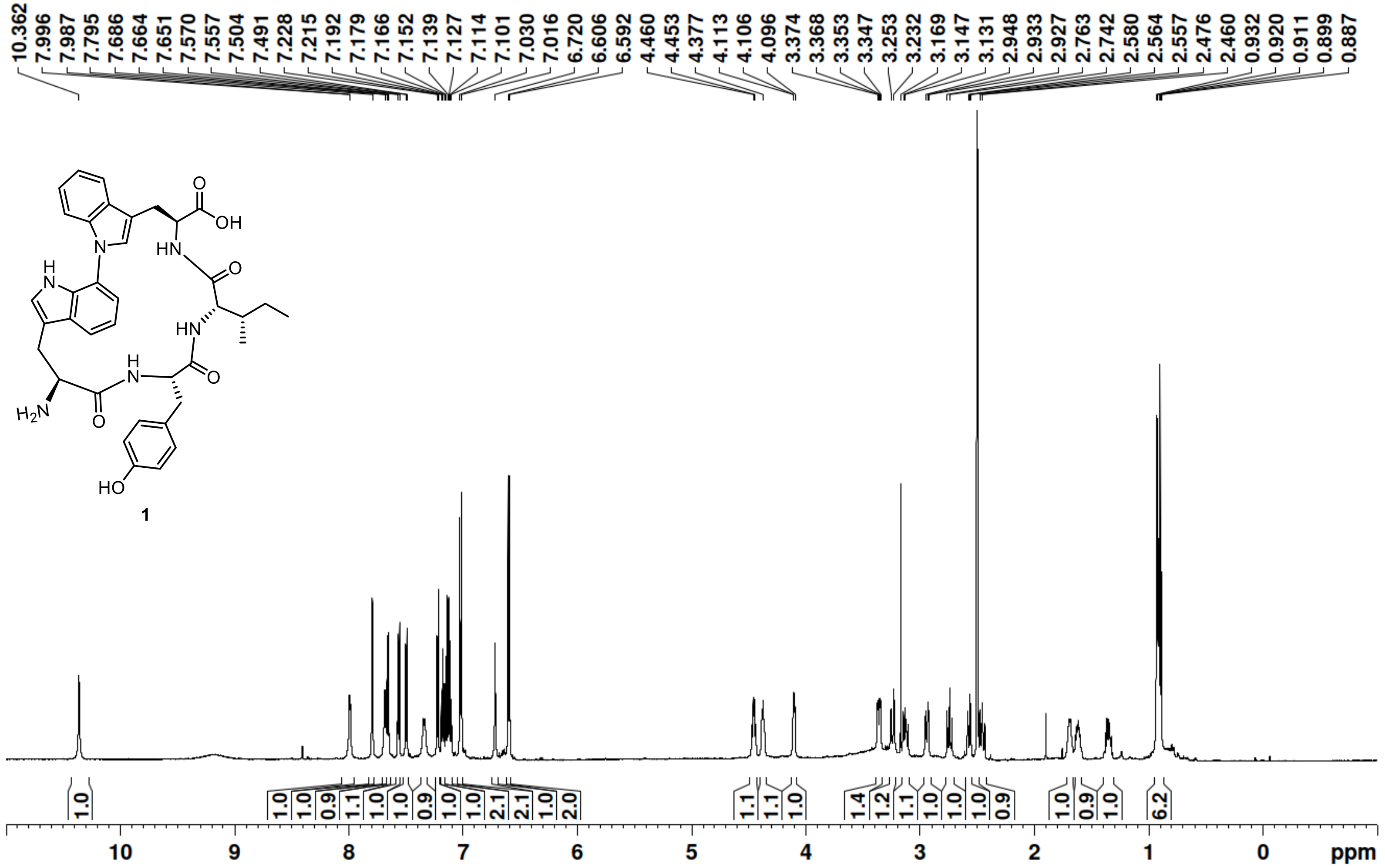

Figure S18. ^{1}H NMR spectrum (600 MHz) of scabrirubin CB-1 (**1**) in DMSO-d_6.

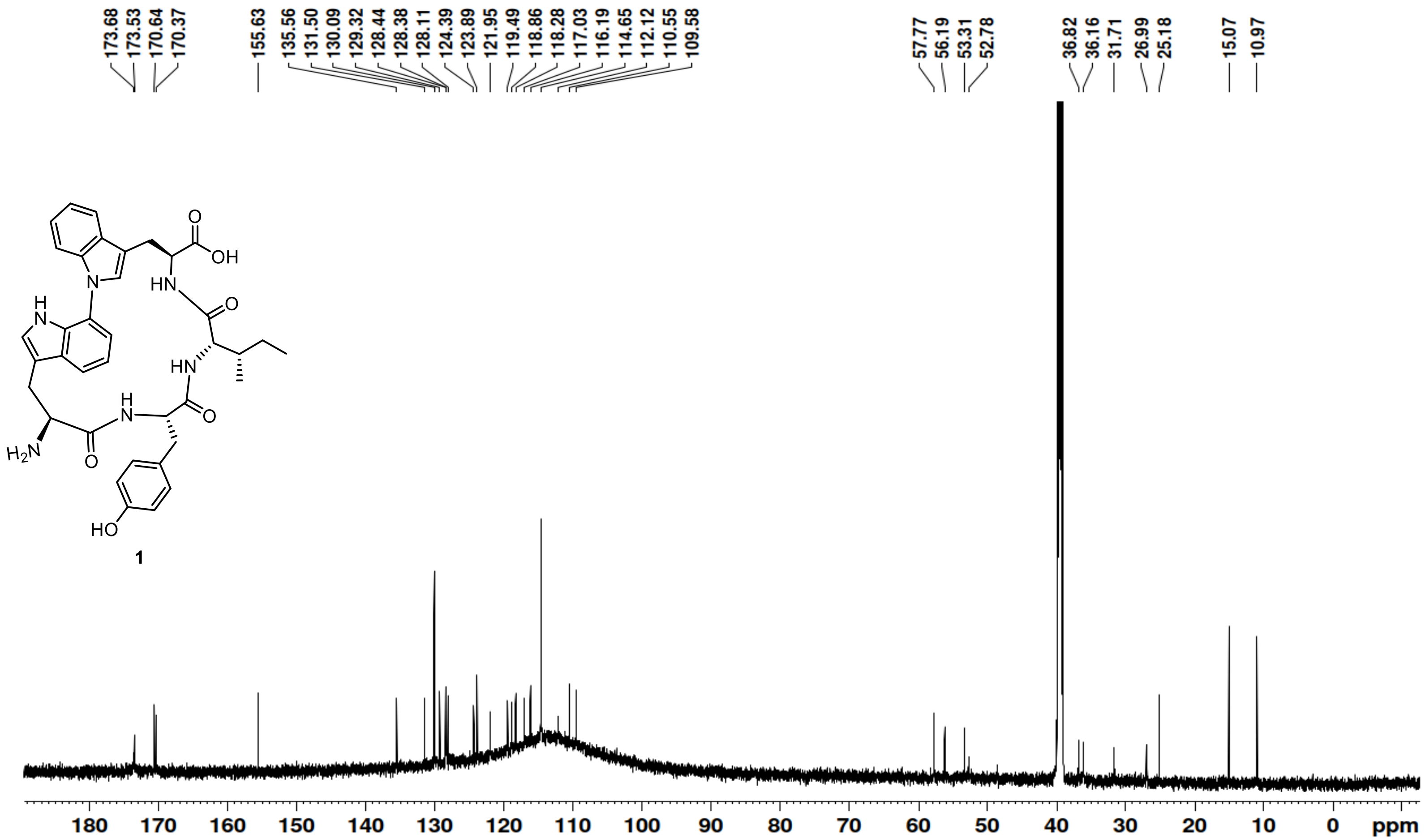

Figure S19. ¹³C NMR spectrum (150 MHz) of scabrirubin CB-1 (**1**) in DMSO-d_6.

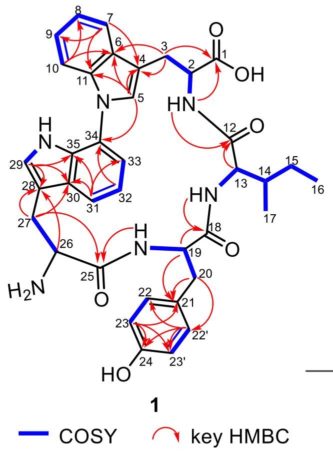

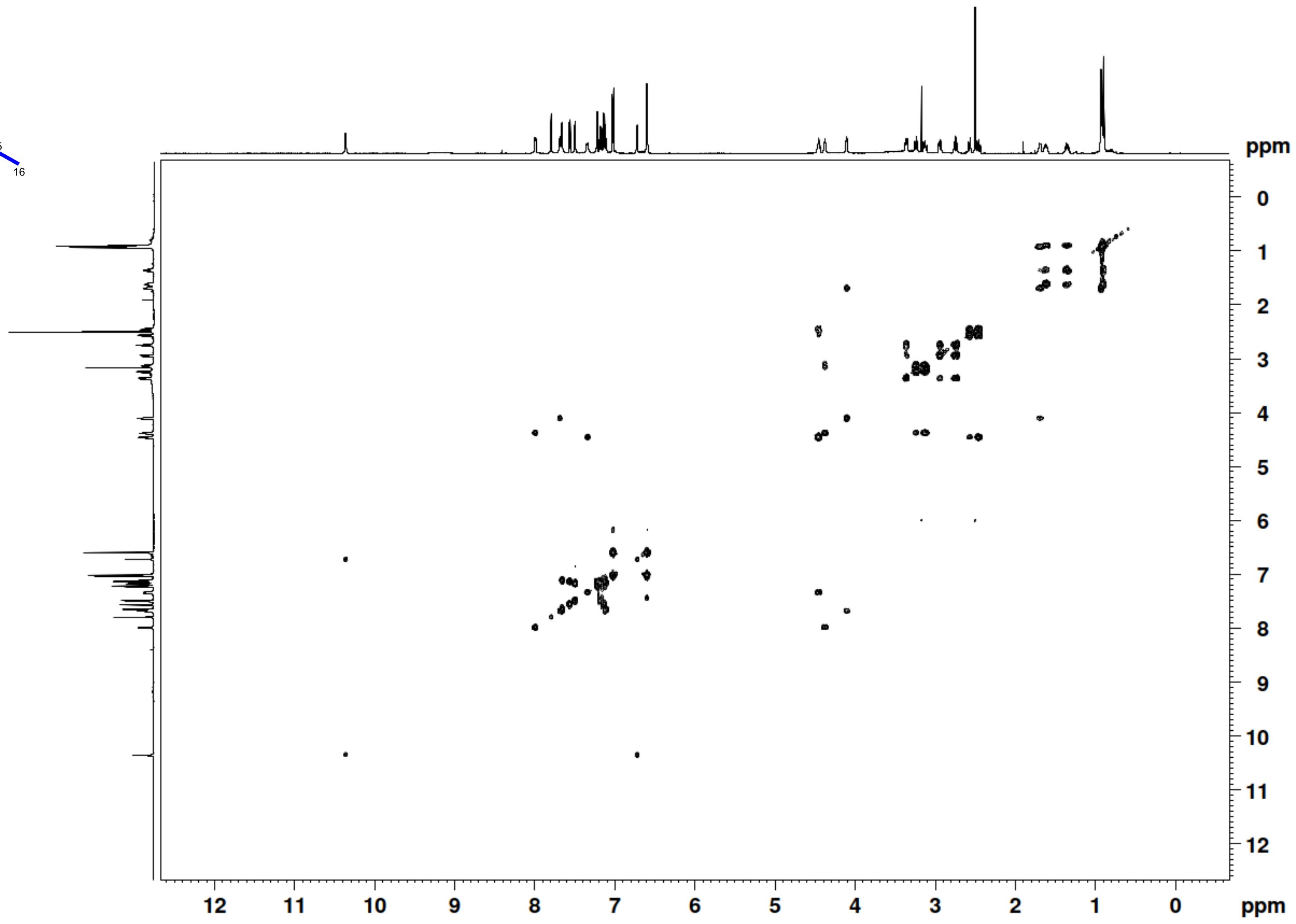

Figure S20. COSY spectrum (600 MHz) of scabrirubin CB-1 (**1**) in DMSO-d_6.

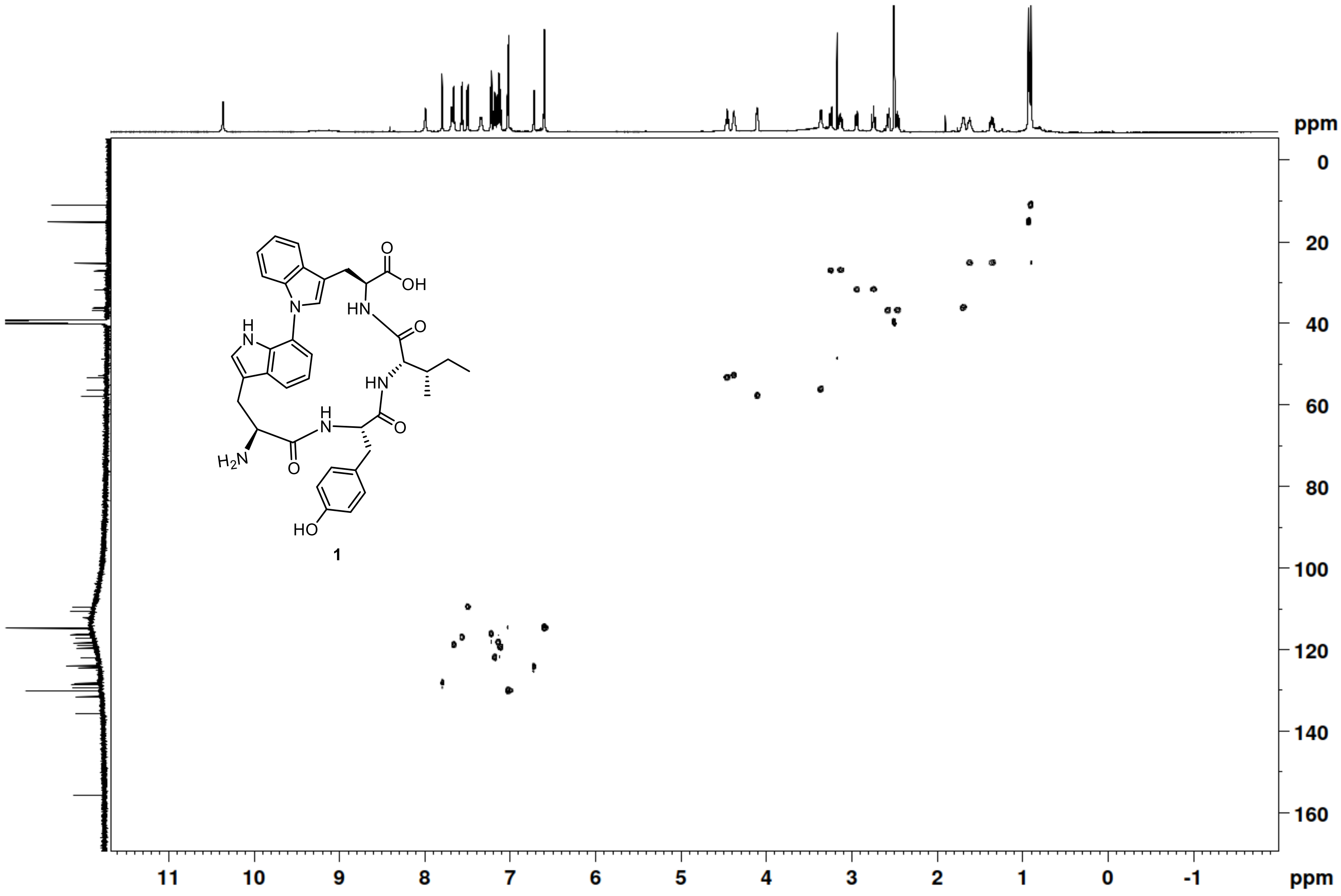

Figure S21. HSQC spectrum (600 MHz) of scabrirubin CB-1 (**1**) in DMSO-d_6.

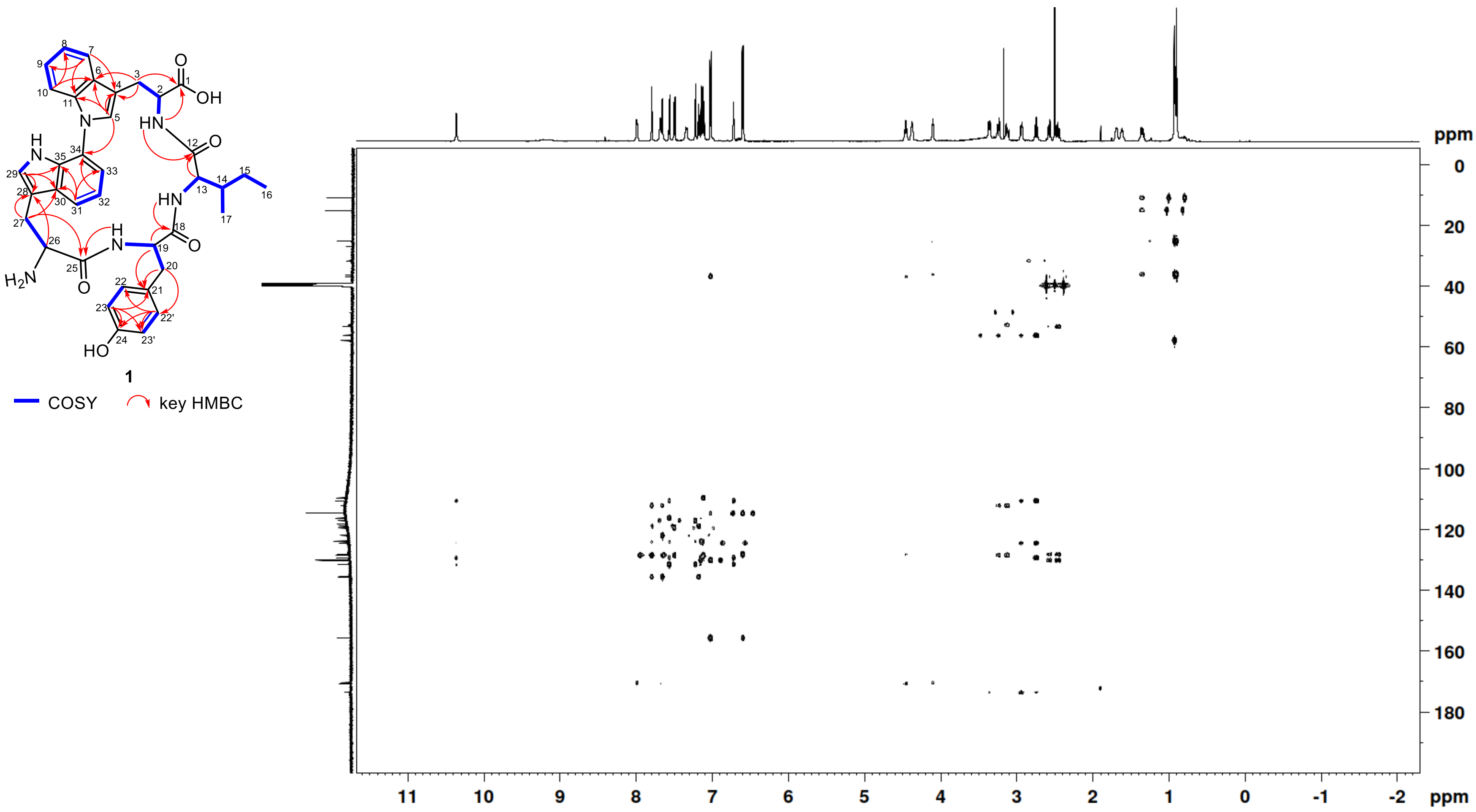

Figure S22. HMBC spectrum (600 MHz) of scabrirubin CB-1 (**1**) in DMSO-d_6.

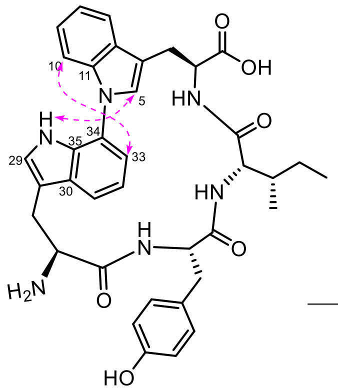

1

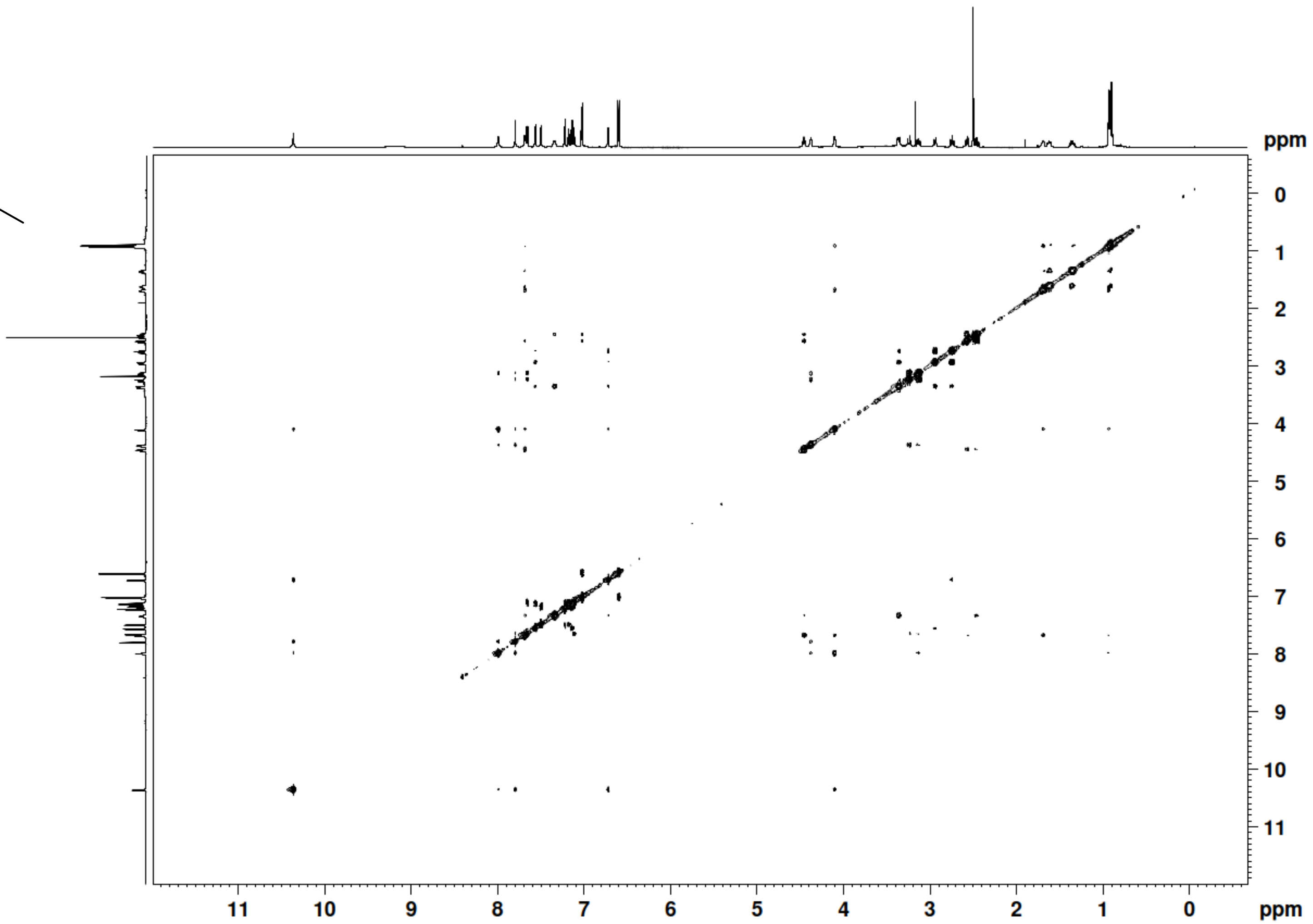

Figure S23. NOESY spectrum (600 MHz) of scabrirubin CB-1 (**1**) in DMSO-d_6.

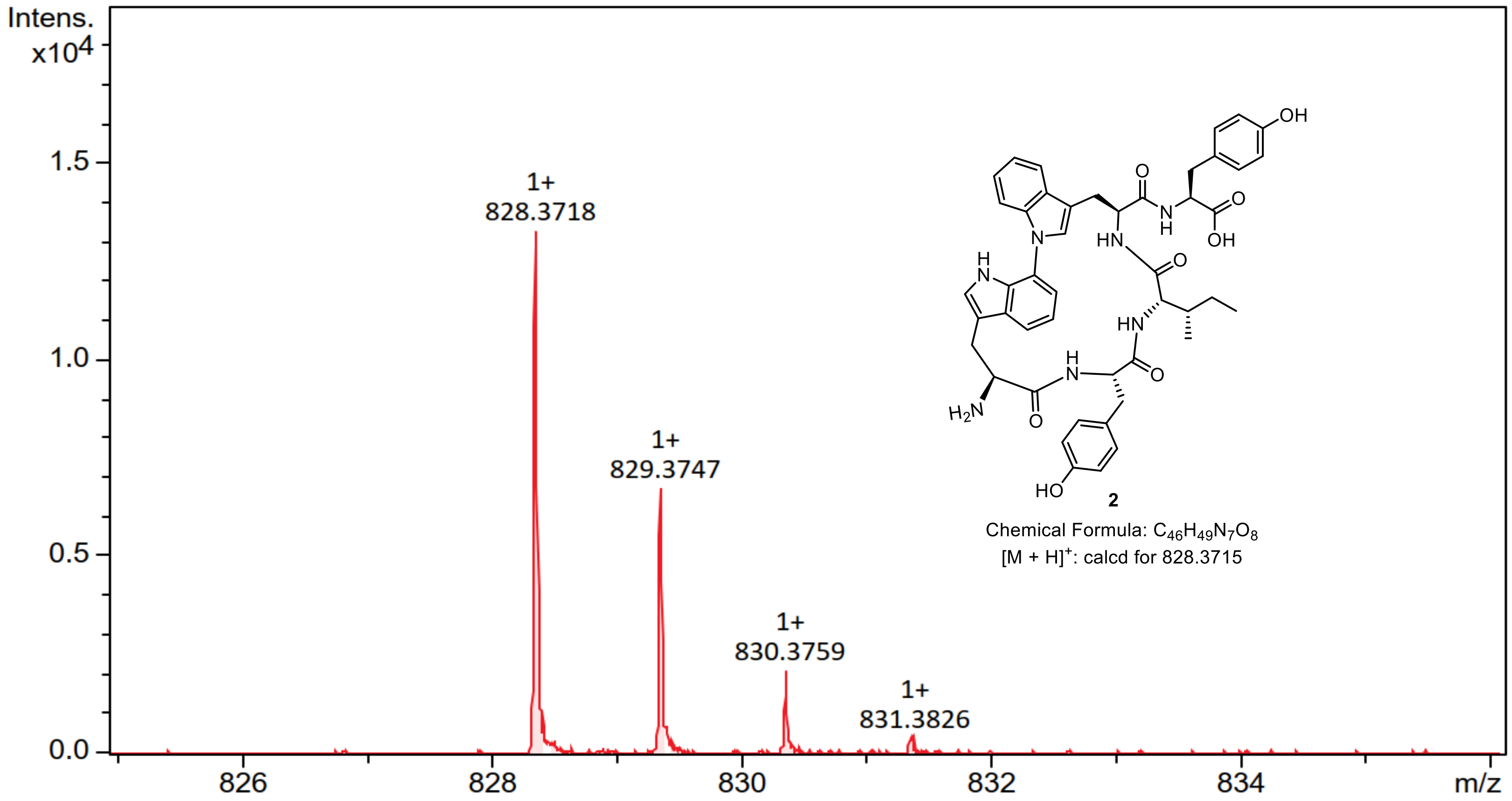

Figure S24. HPLC-ESI-QTOF-HRMS analysis of scabrirubin CB-2 (**2**).

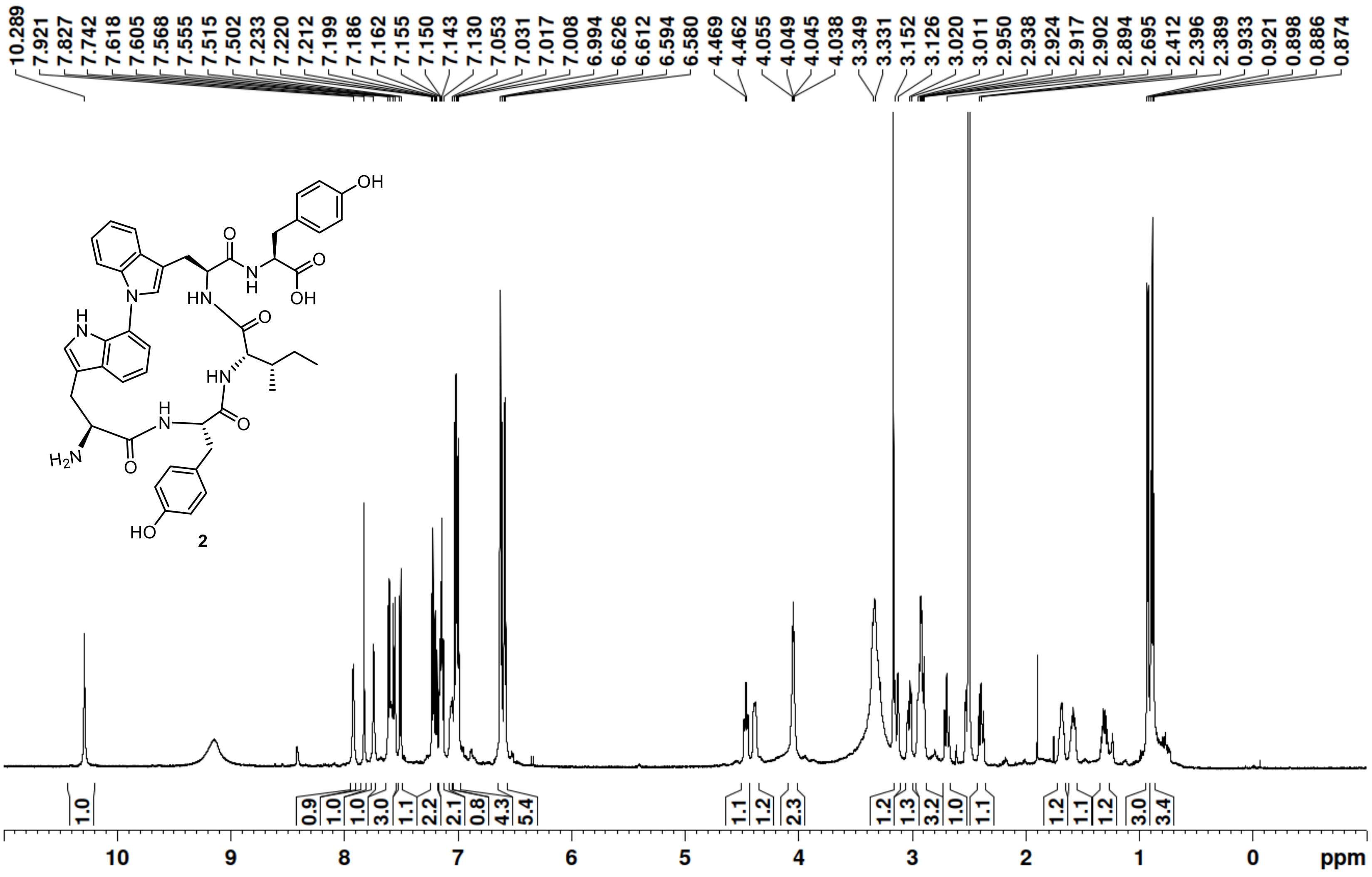

Figure S25. ^{1}H NMR spectrum (600 MHz) of scabrirubin CB-2 (**2**) in DMSO-d_6

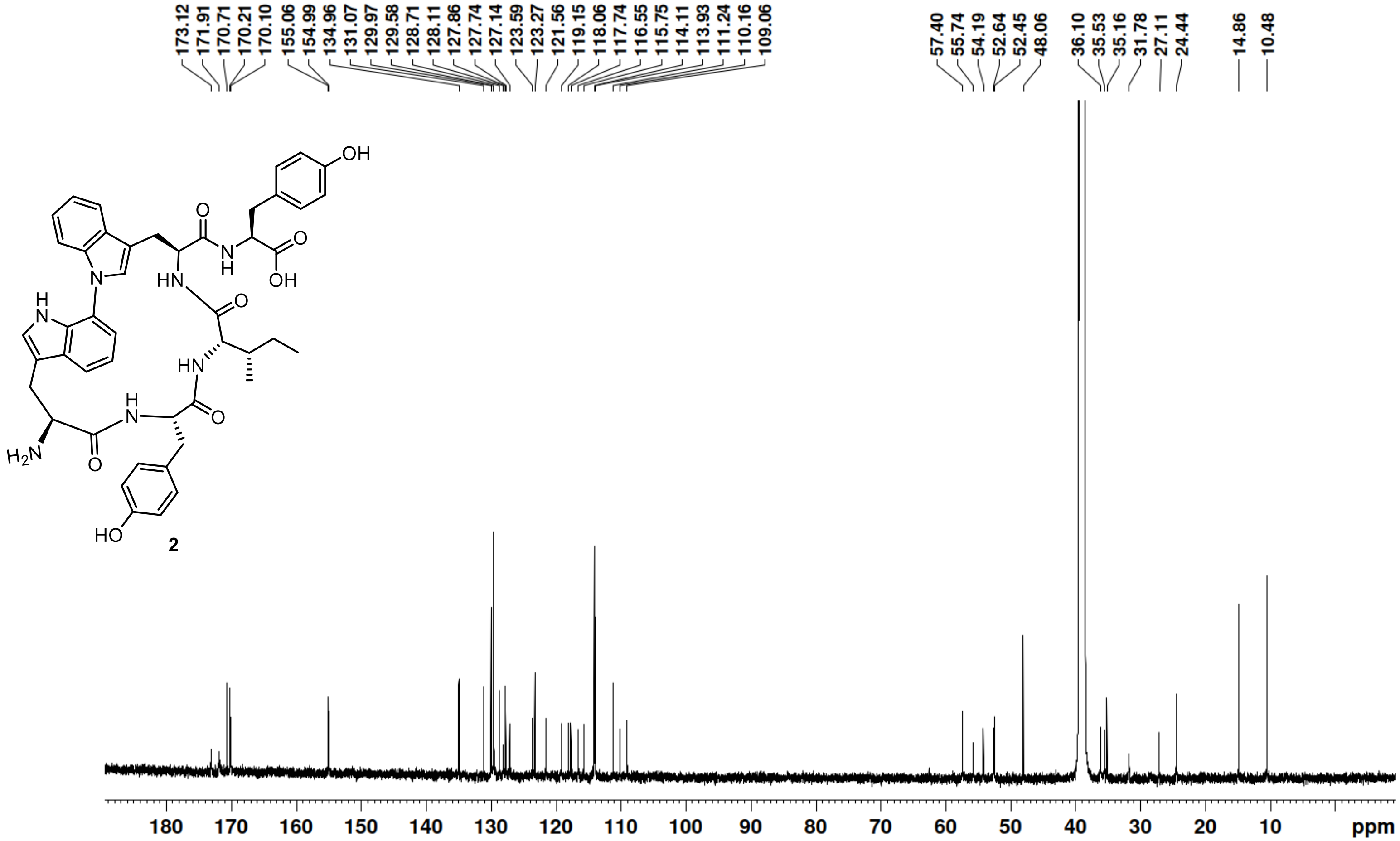

Figure S26. ^{13}C NMR spectrum (125 MHz) of scabrirubin CB-2 (**2**) in DMSO-d_6.

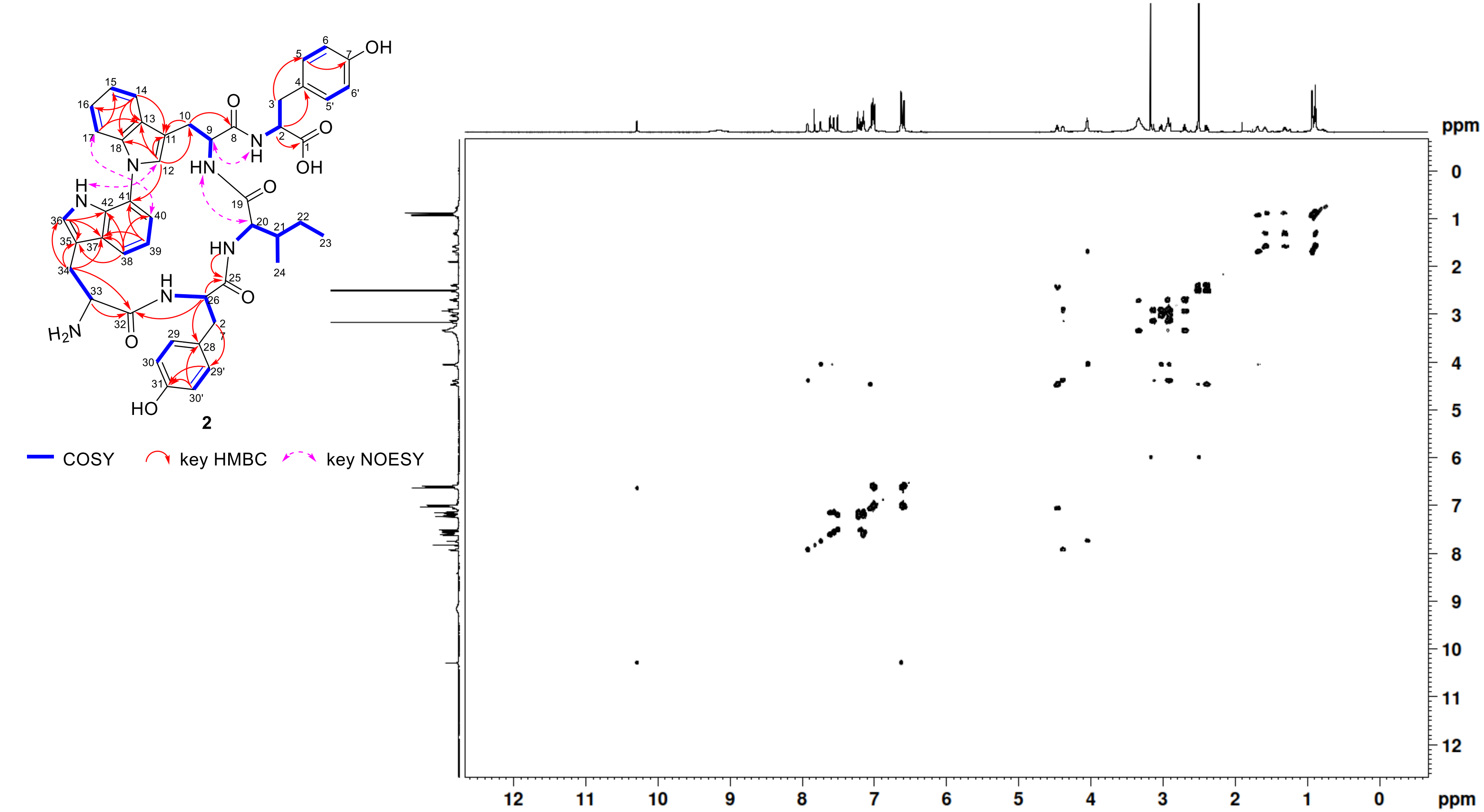

Figure S27. COSY spectrum (500 MHz) of scabrirubin CB-2 (**2**) in DMSO-d_6.

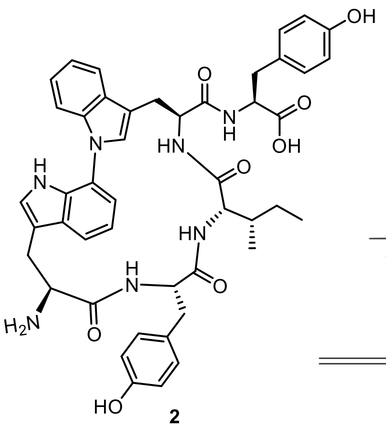

2

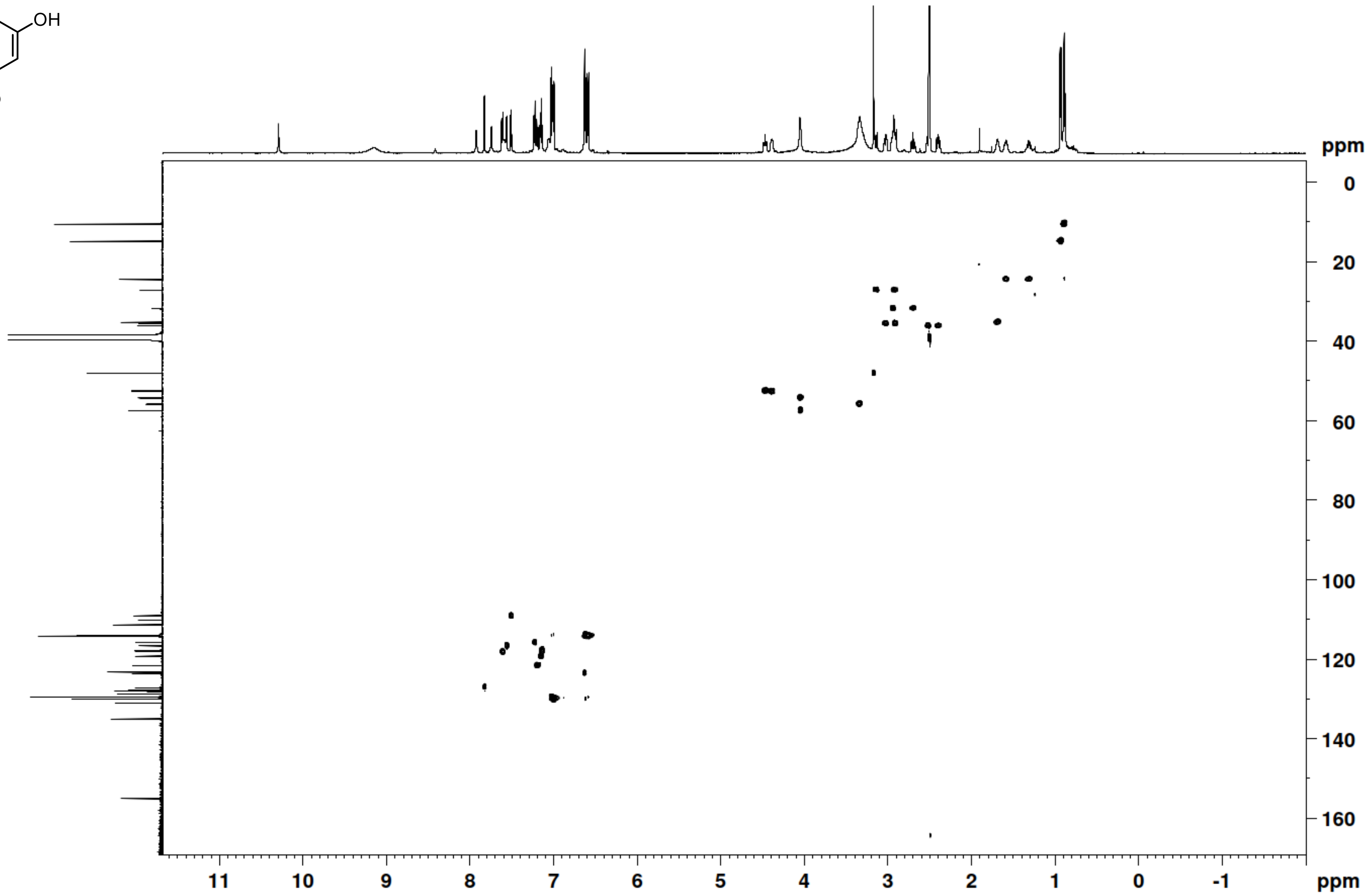

Figure S28. HSQC spectrum (500 MHz) of scabrirubin CB-2 (**2**) in DMSO-d_6.

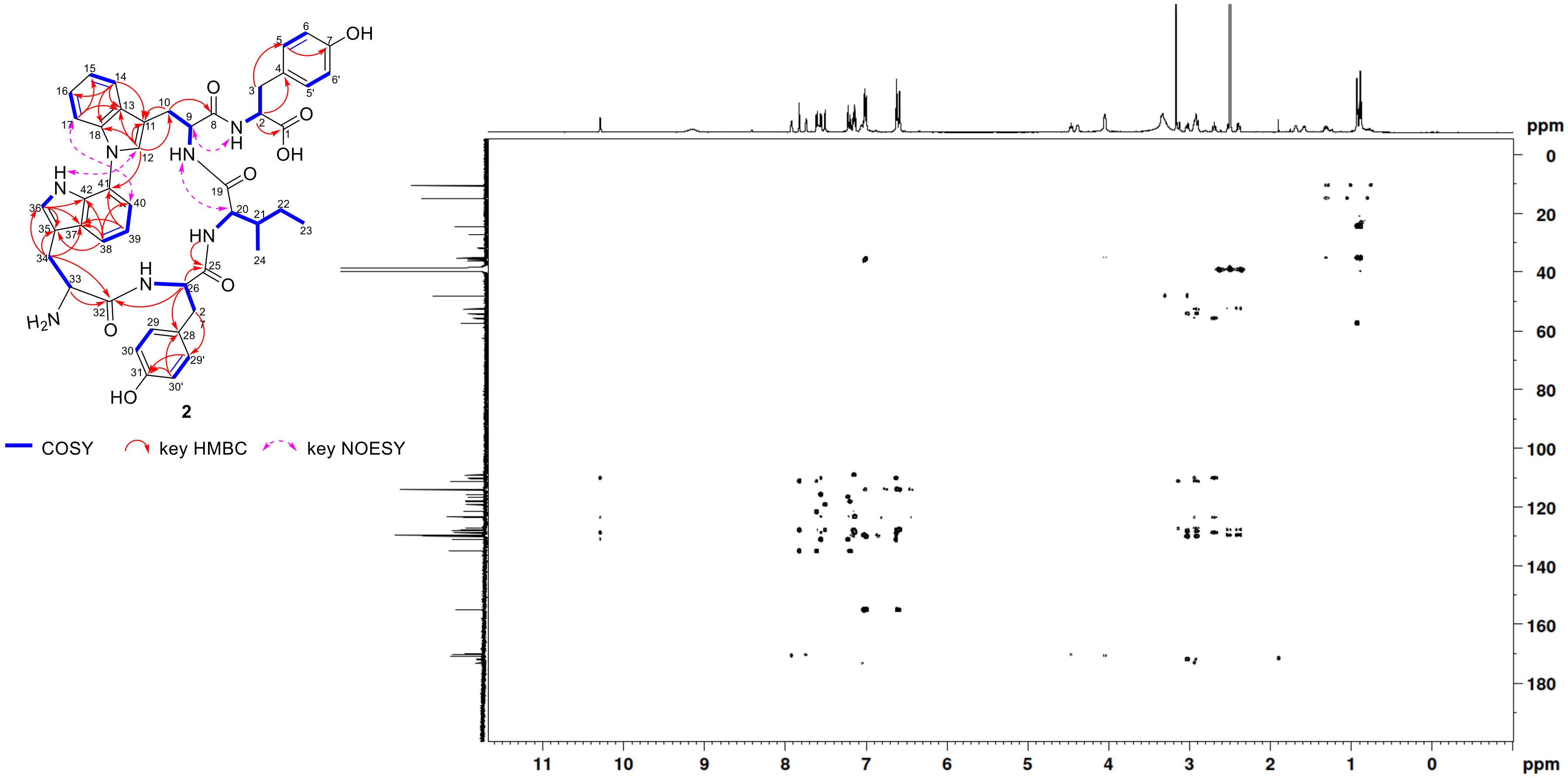

Figure S29. HMBC spectrum (500 MHz) of scabrirubin CB-2 (**2**) in DMSO-d_6.

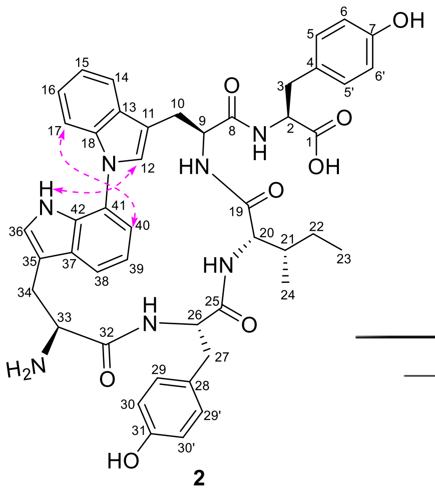

2

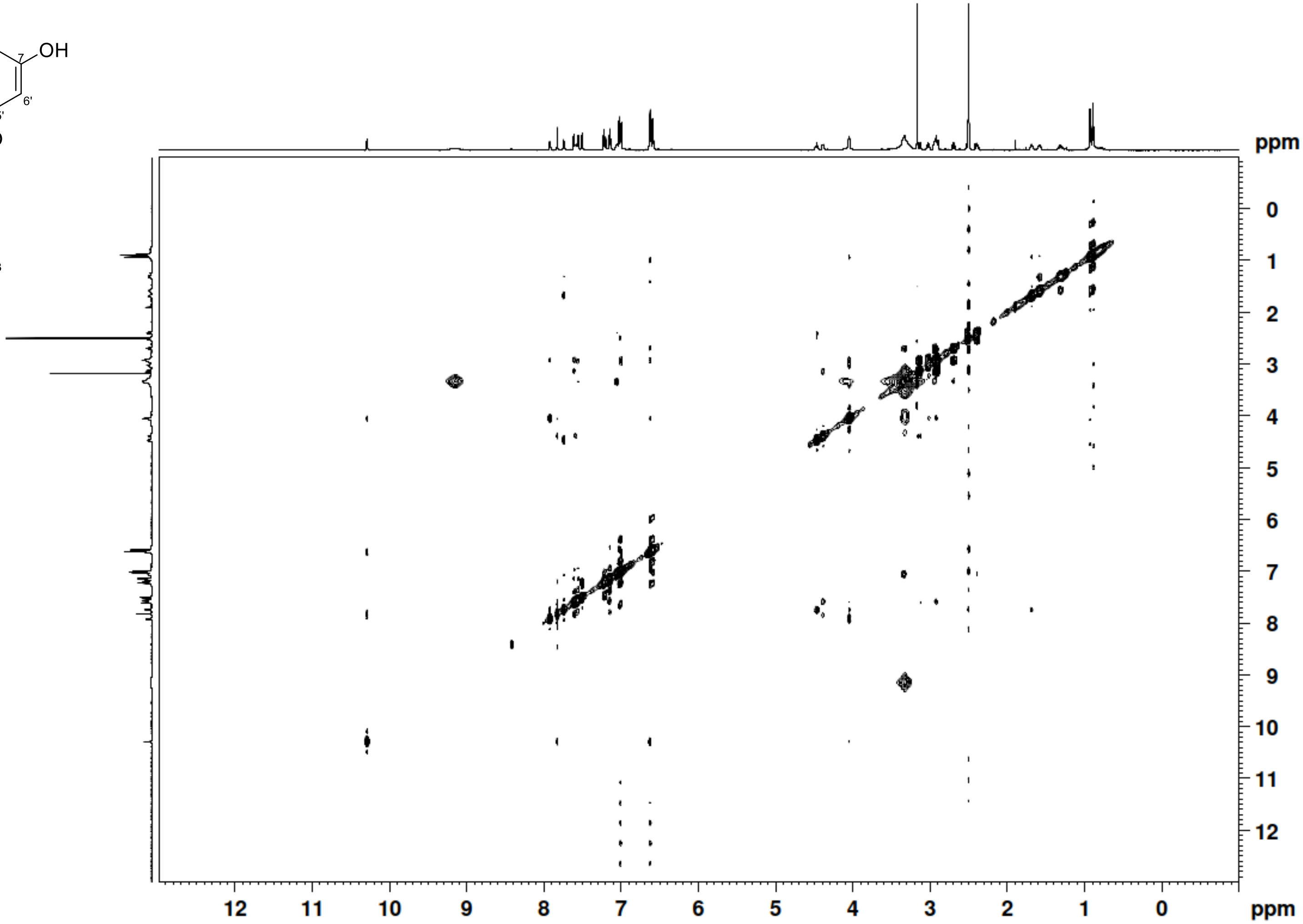

Figure S30. NOESY spectrum (500 MHz) of scabrirubin CB-2 (**2**) in DMSO-d_6.

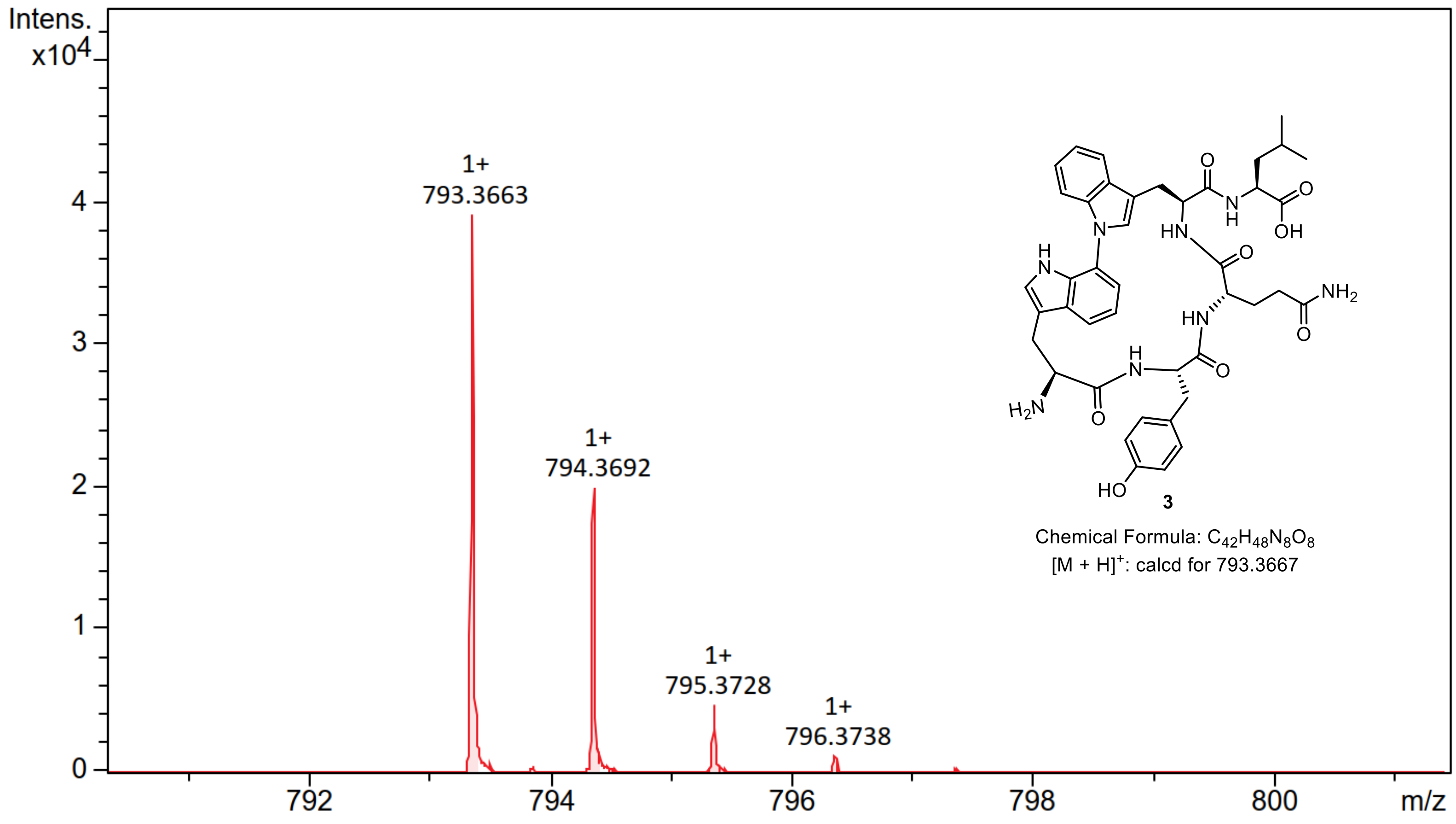

Figure S31. HPLC-ESI-QTOF-HRMS analysis of scabrirubin CB-3 (**3**).

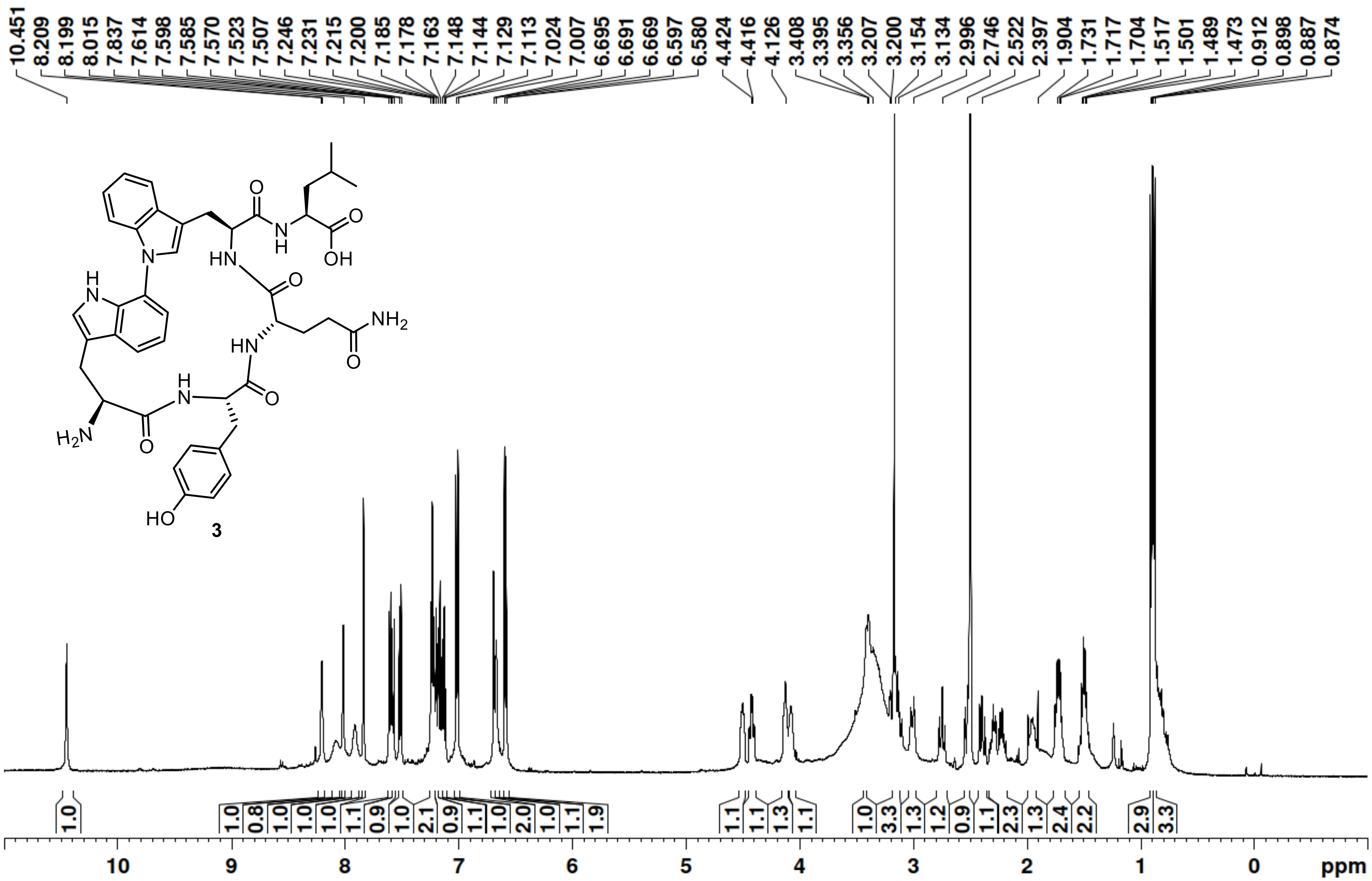

Figure S32. ^{1}H NMR spectrum (500 MHz) of scabrirubin CB-3 (**3**) in DMSO-d_6.

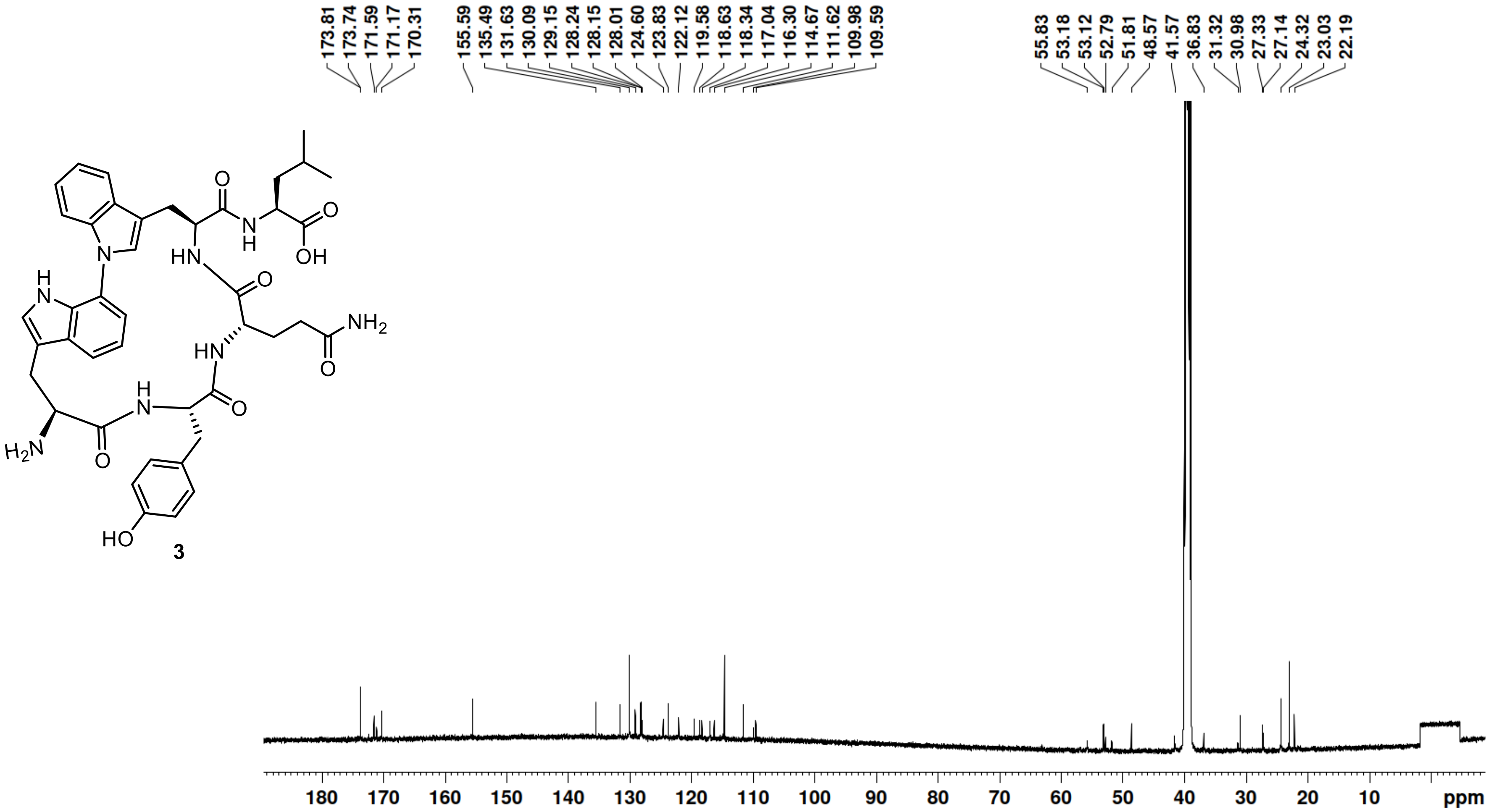

Figure S33. ^{13}C NMR spectrum (125 MHz) of scabrirubin CB-3 (**3**) in DMSO-d_6.

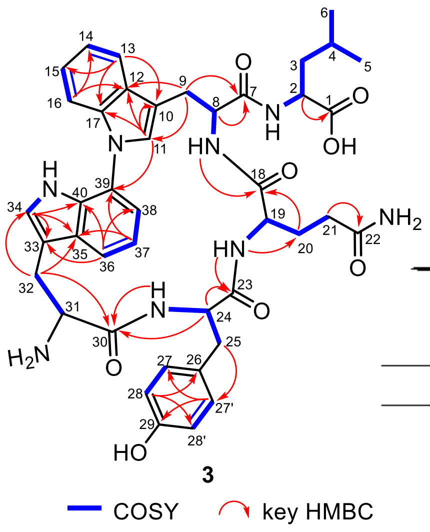

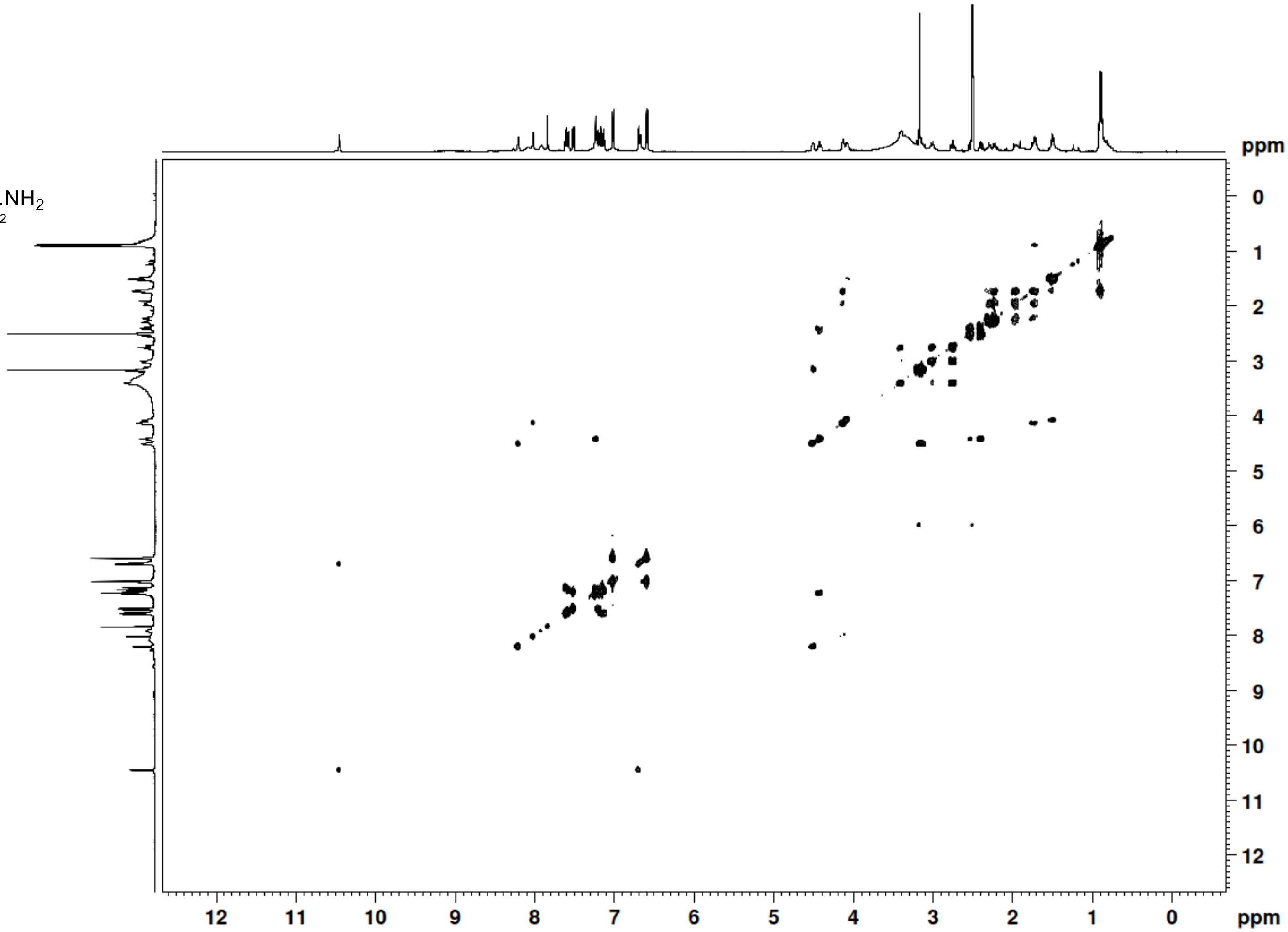

Figure S34. COSY spectrum (500 MHz) of scabrirubin CB-3 (**3**) in DMSO-d_6.

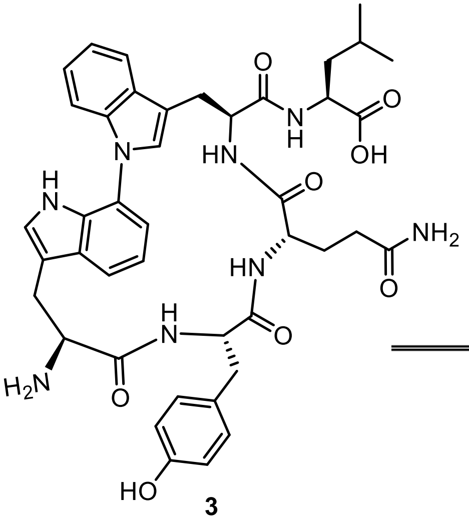

3

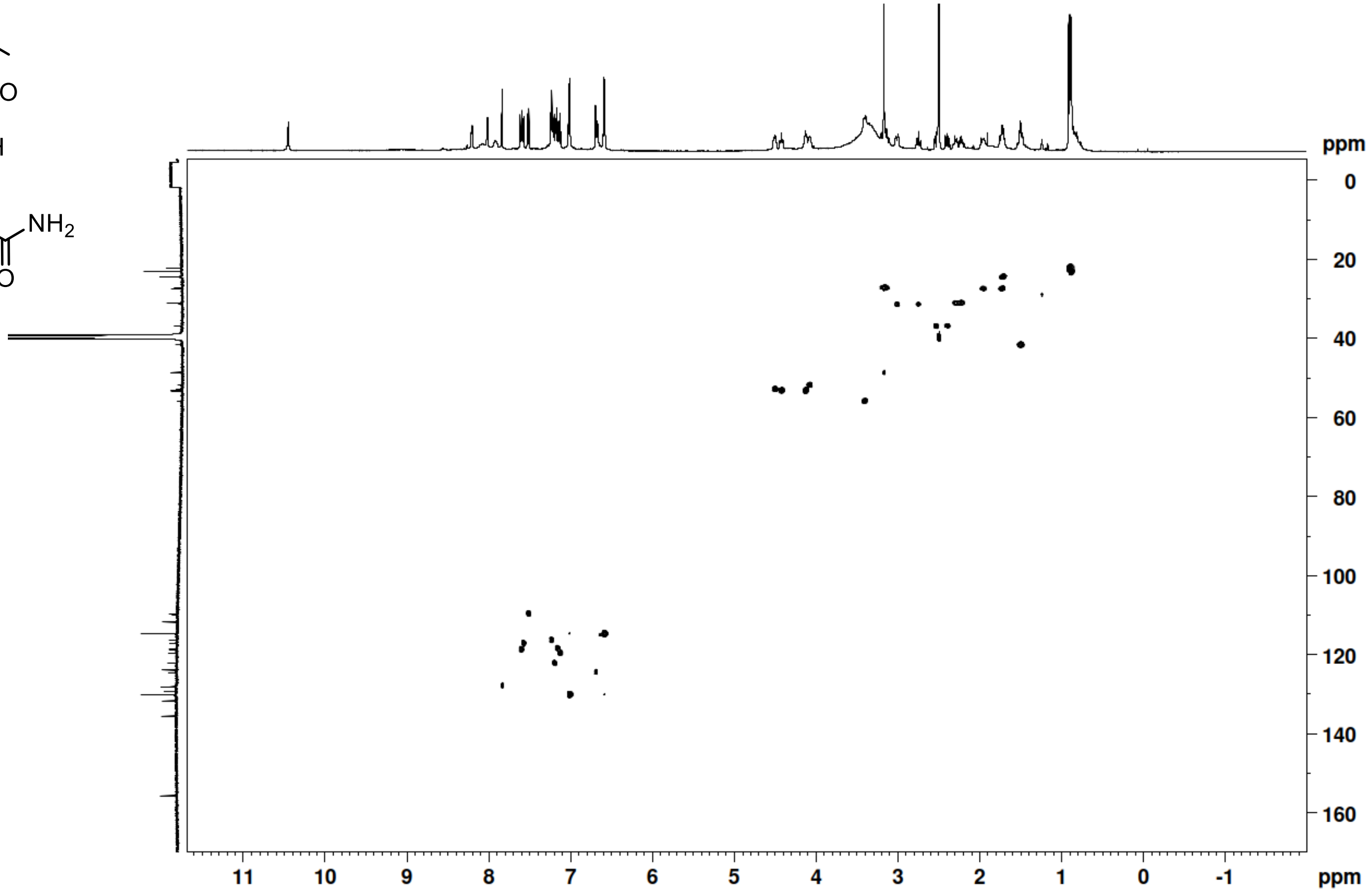

Figure S35. HSQC spectrum (500 MHz) of scabrirubin CB-3 (**3**) in DMSO-d_6.

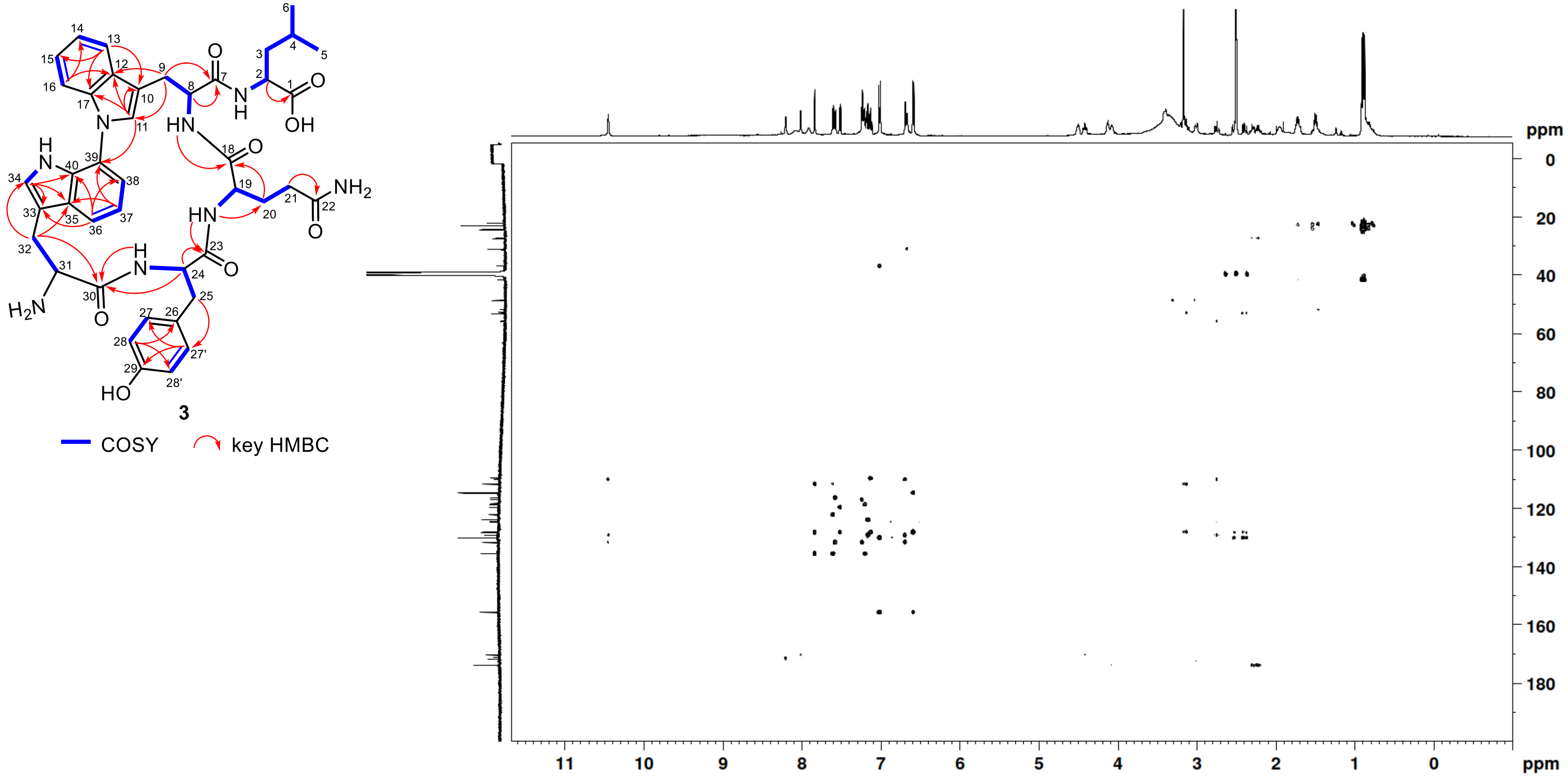

Figure S36. HMBC spectrum (500 MHz) of scabrirubin CB-3 (**3**) in DMSO-d_6.

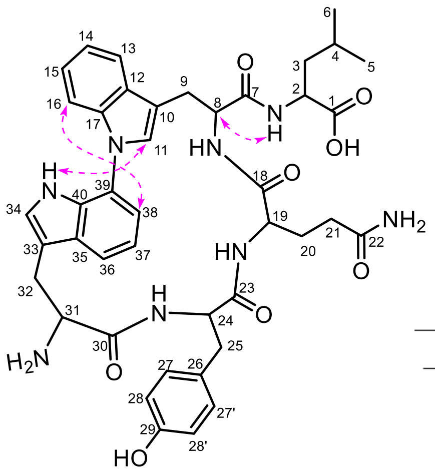

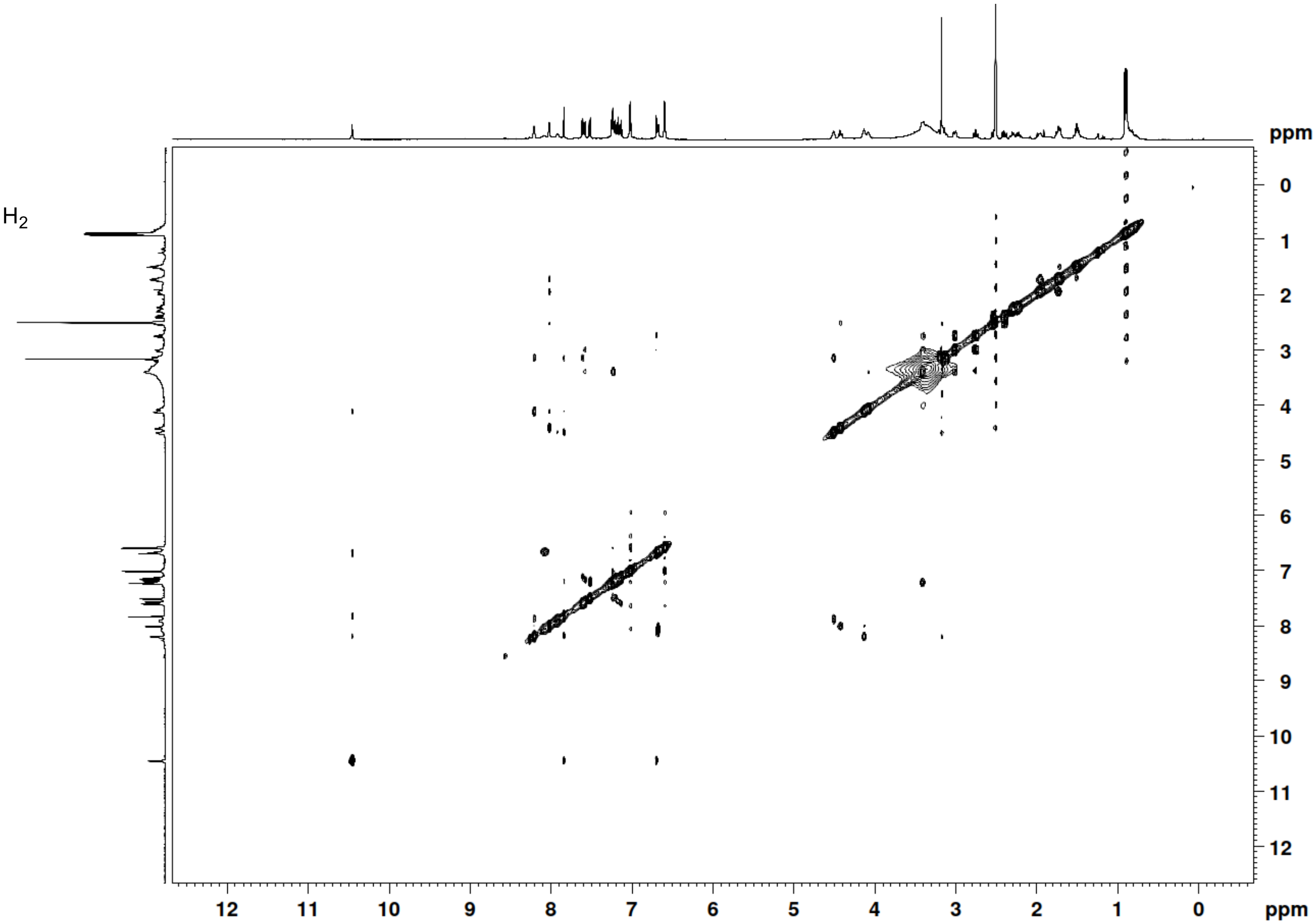

Figure S37. NOESY spectrum (500 MHz) of scabrirubin CB-3 (**3**) in DMSO-d_6.

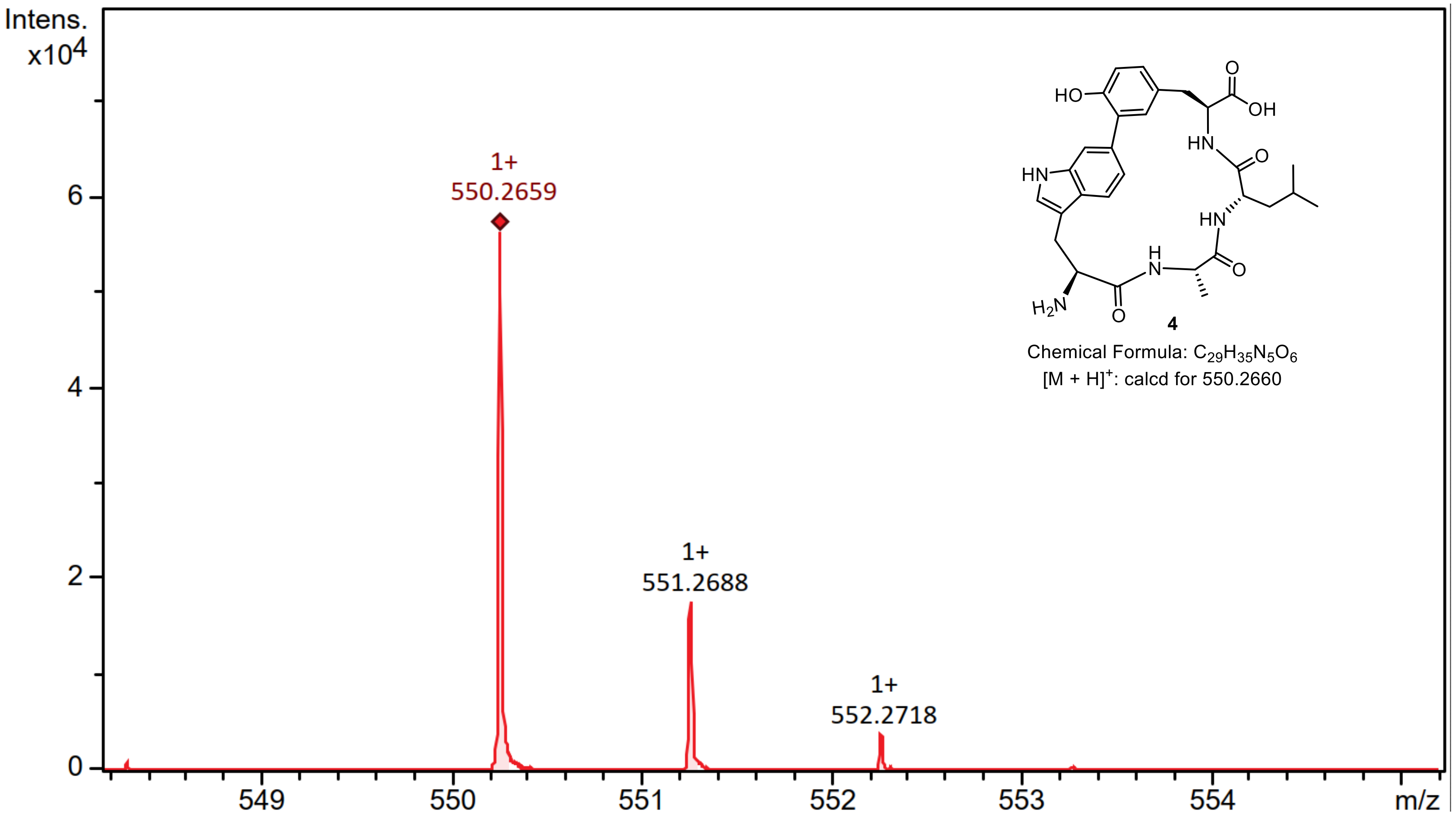

Figure S38. HPLC-ESI-QTOF-HRMS analysis of scabrirubin CB-4 (**4**).

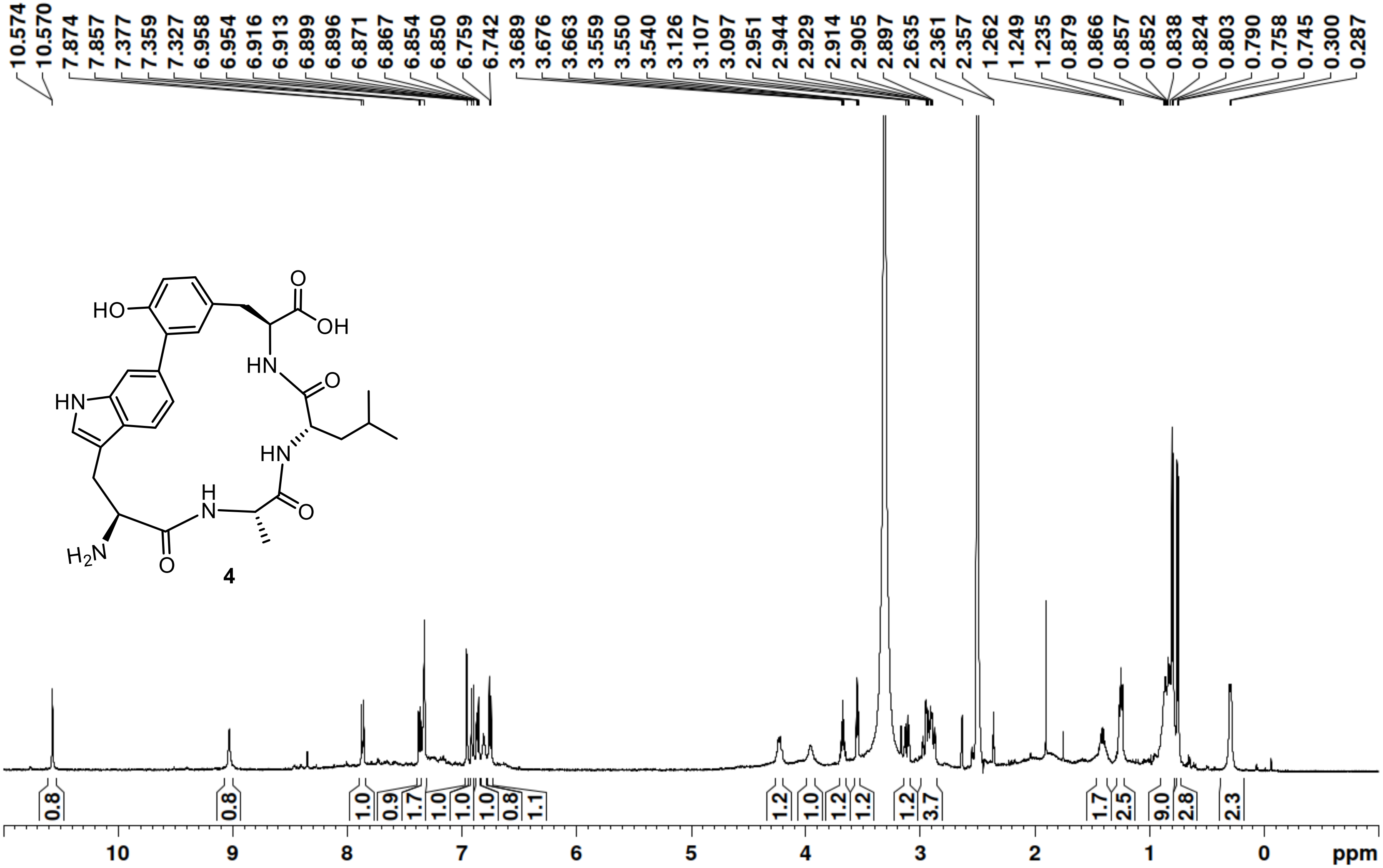

Figure S39. ^{1}H NMR spectrum (500 MHz) of scabrirubin CB-4 (**4**) in DMSO-d_6.

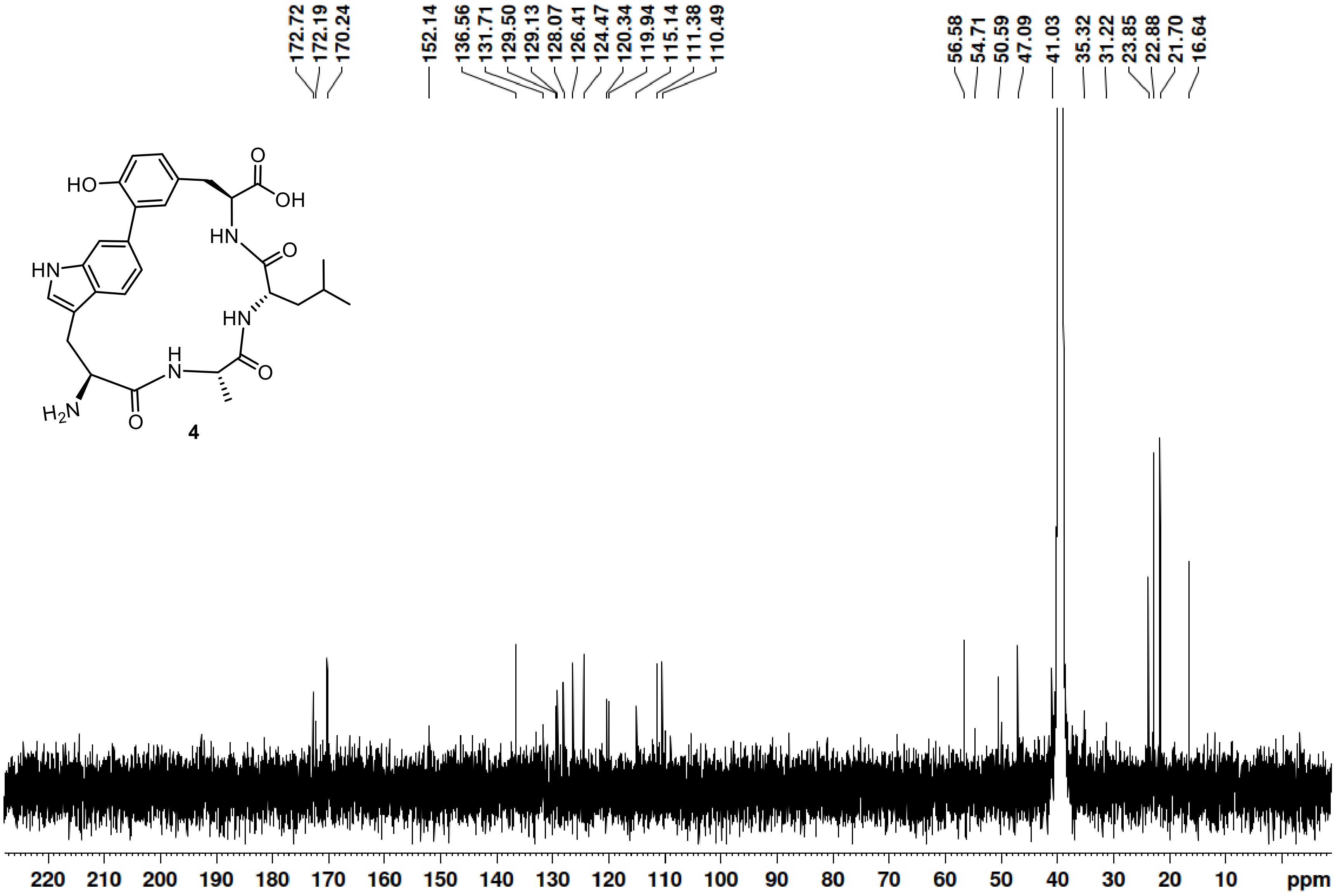

Figure S40. ^{13}C NMR spectrum (125 MHz) of scabrirubin CB-4 (**4**) in DMSO-d_6.

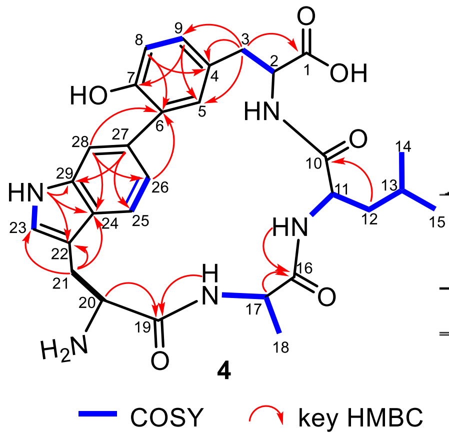

4

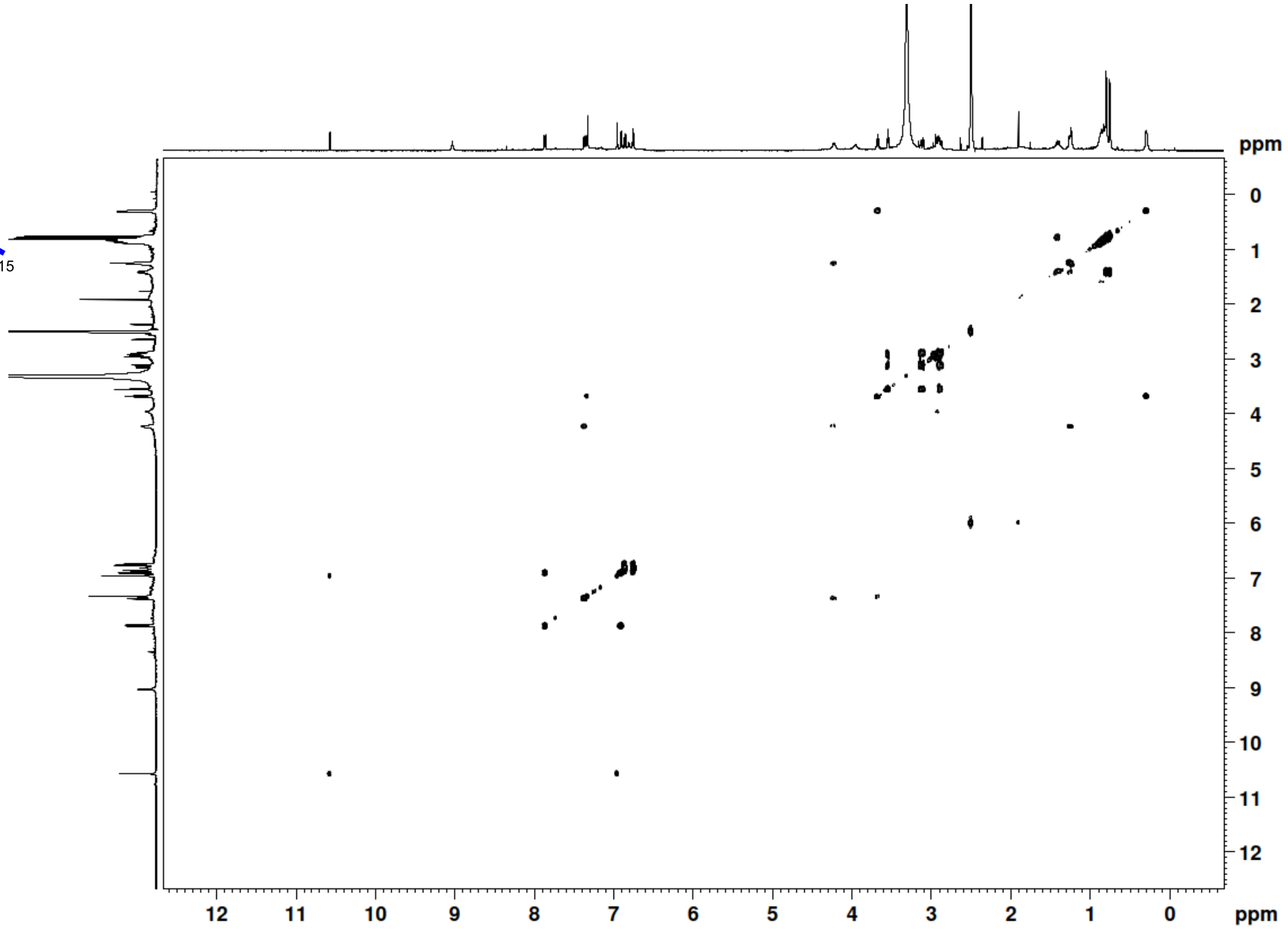

Figure S41. COSY spectrum (500 MHz) of scabrirubin CB-4 (**4**) in DMSO-d_6.

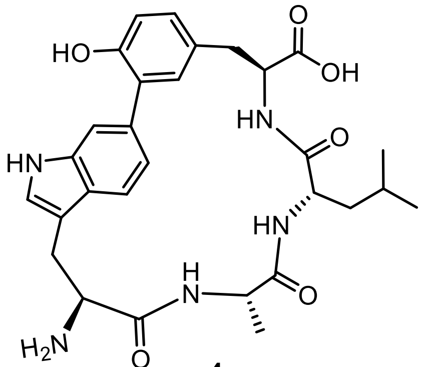

4

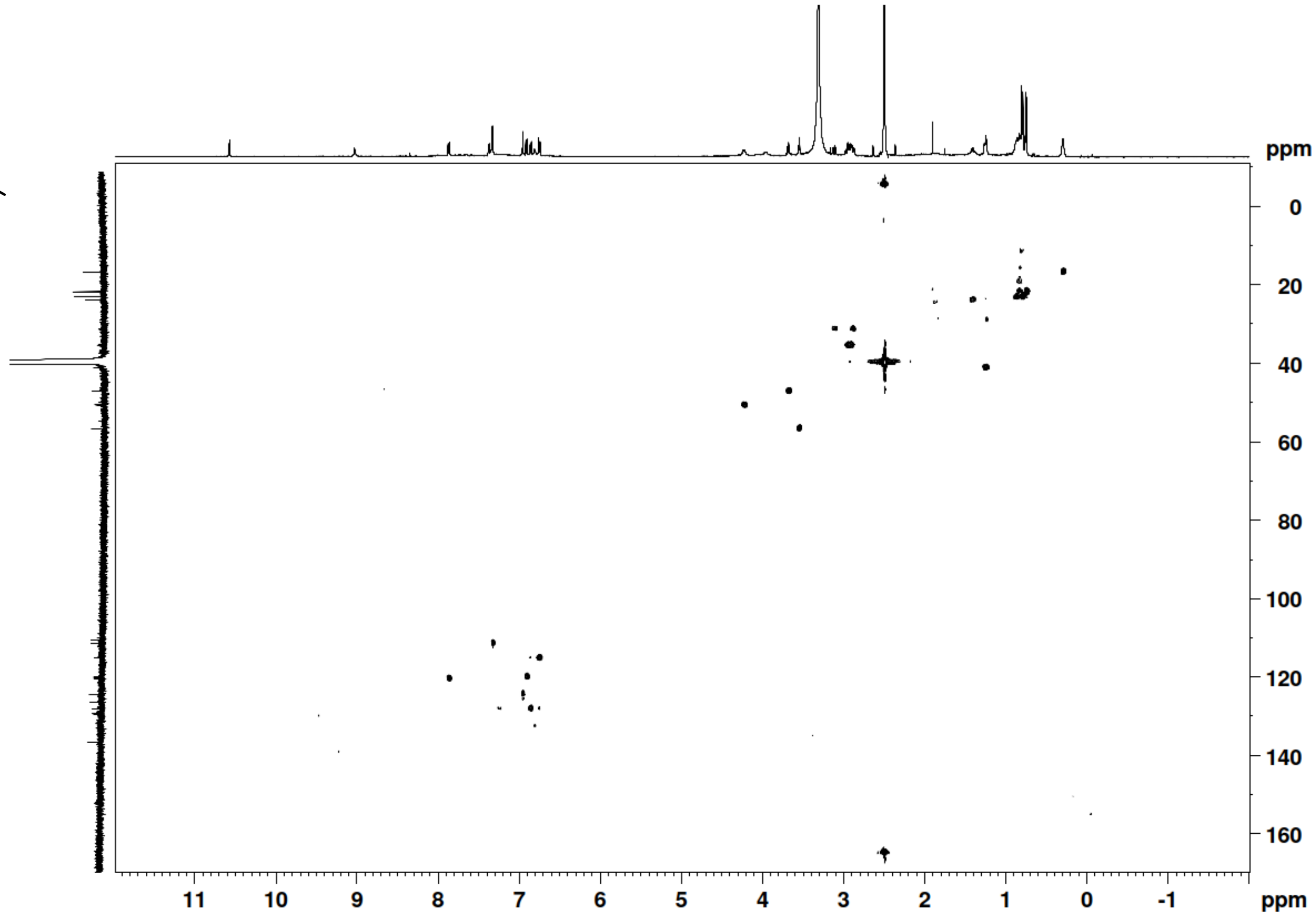

Figure S42. HSQC spectrum (500 MHz) of scabrirubin CB-4 (**4**) in DMSO-d_6.

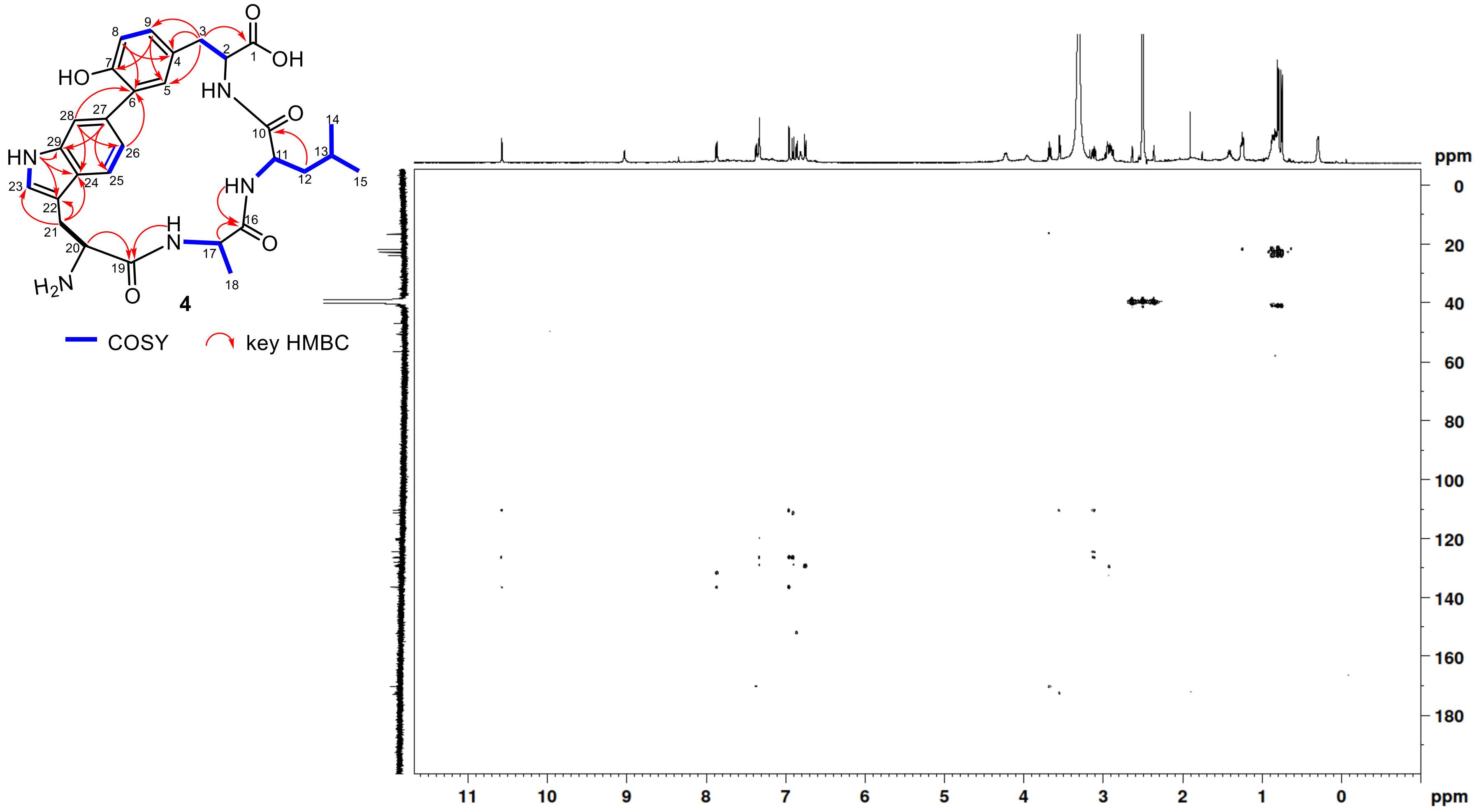

Figure S43. HMBC spectrum (500 MHz) of scabrirubin CB-4 (**4**) in DMSO-d_6.

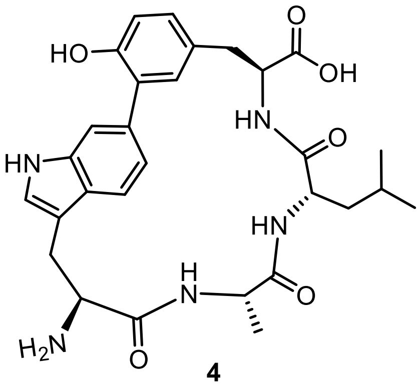

4

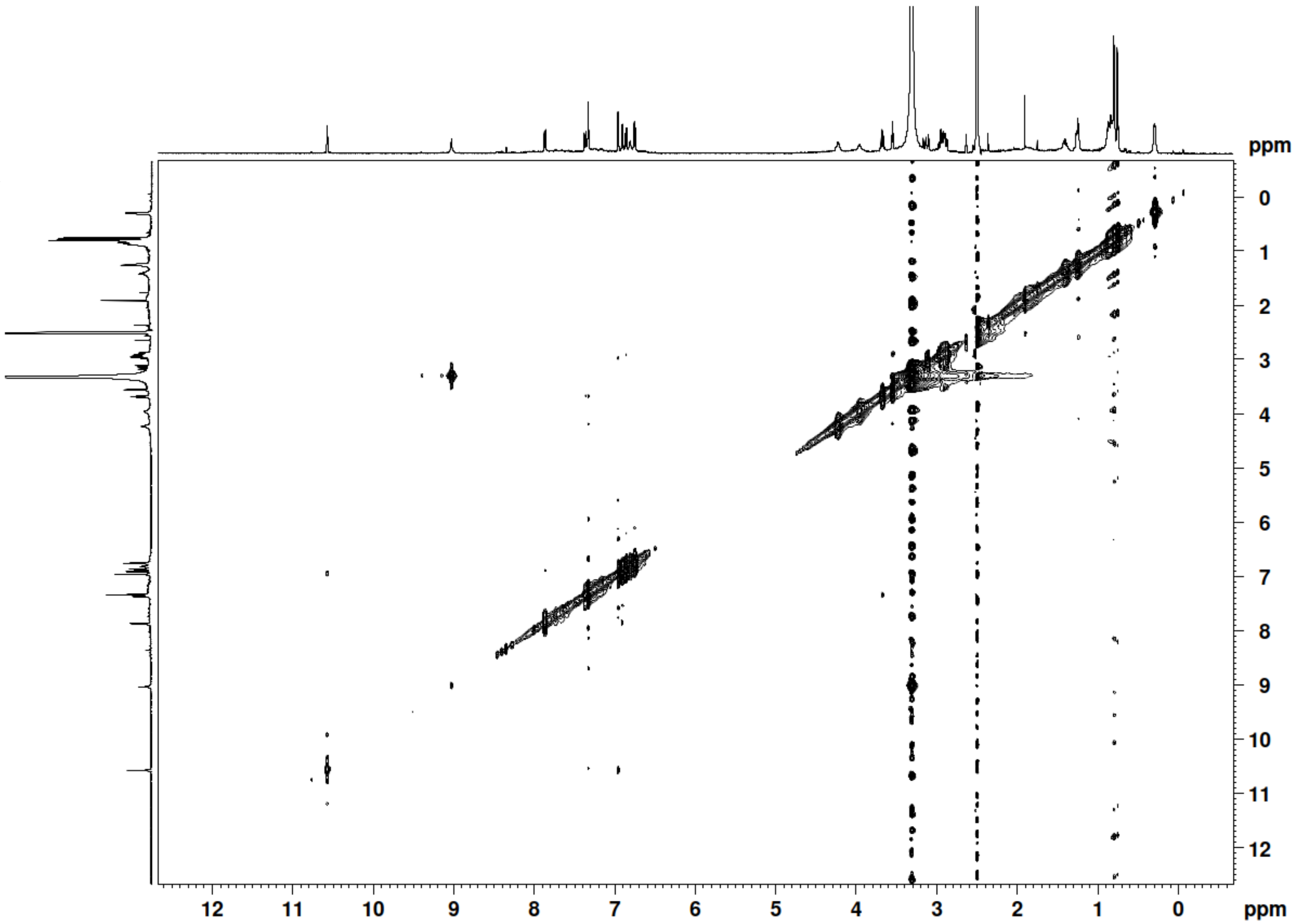

Figure S44. NOESY spectrum (500 MHz) of scabrirubin CB-4 (**4**) in DMSO-d_6.

A

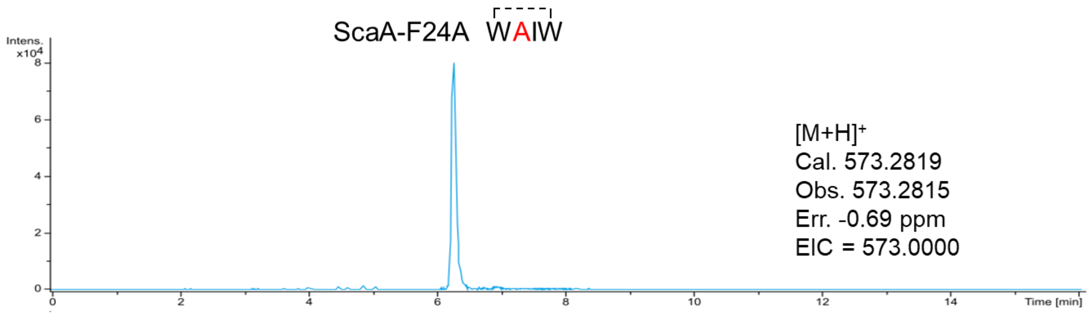

B

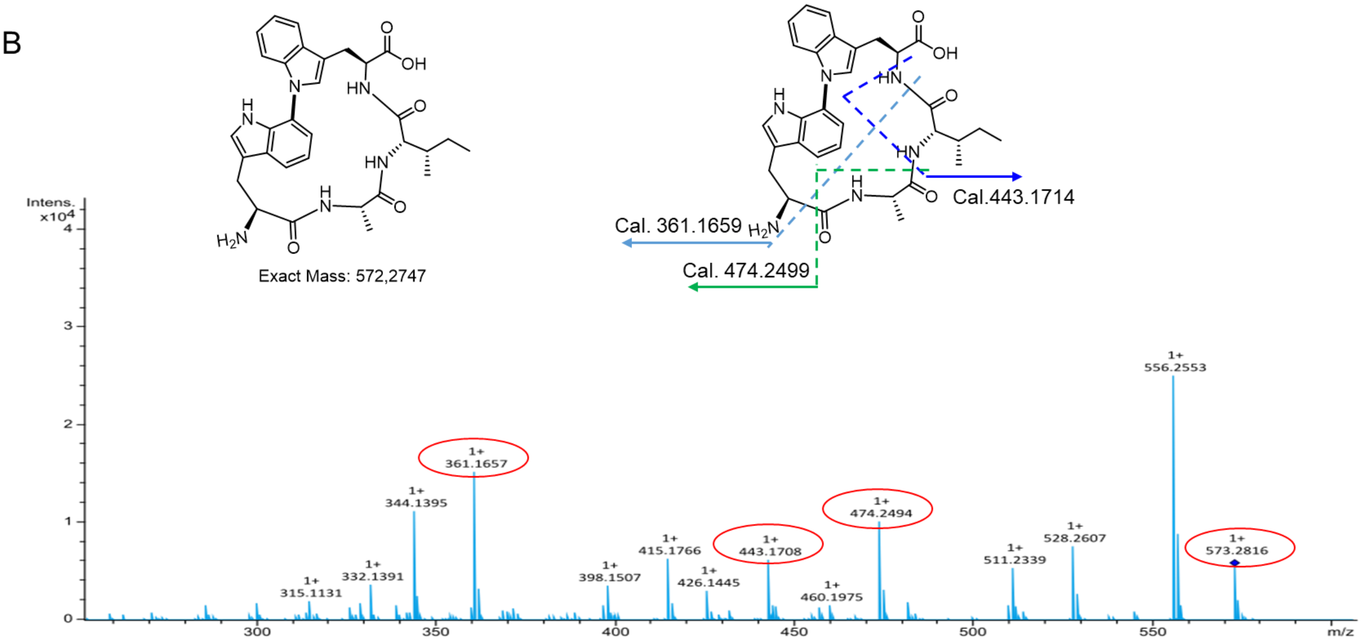

Figure S45. HRMS analysis of extracts of the recombinant *S. albus* strain harboring *scaA*-F24A and *scaB*. A) Extracted ion chromatogram of the product from the coexpression of *scaA*-F24A with *scaB*; B) MS/MS spectrum of the corresponding atropopeptide detected at *m*/*z* 573.2815 $[M+H]^+$ with key fragments that indicate the presence of a bond between the two Trp residues highlighted.

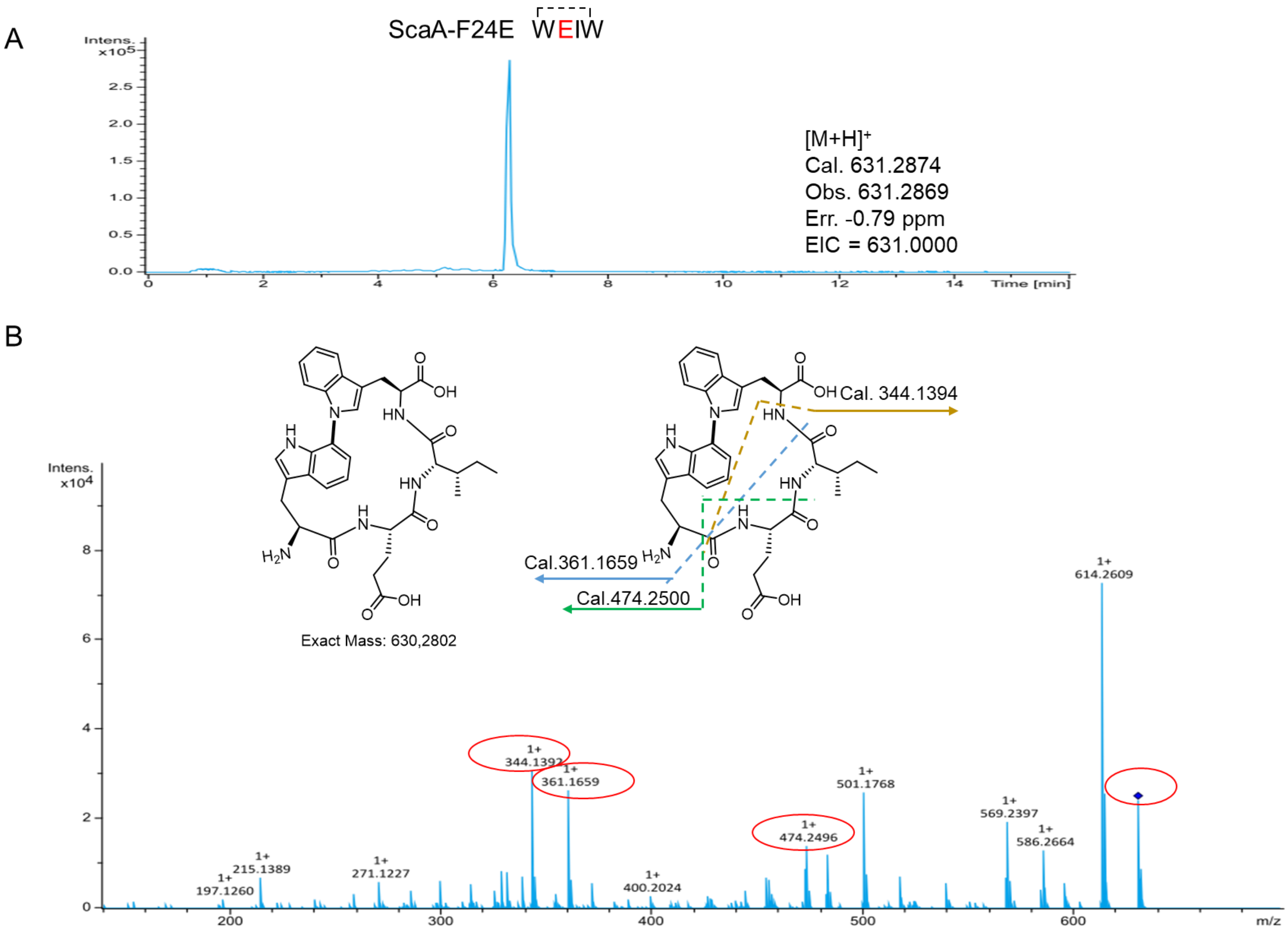

Figure S46. HRMS analysis of extracts of the recombinant *S. albus* strain harboring *scaA*-F24E and *scaB*. A) Extracted ion chromatogram of the product from the coexpression of *scaA*-F24E with *scaB*; B) MS/MS spectrum of the corresponding atropopeptide detected at *m*/*z* 631.2869 $[M+H]^+$ with key fragments that indicate the presence of a bond between the two Trp residues highlighted.

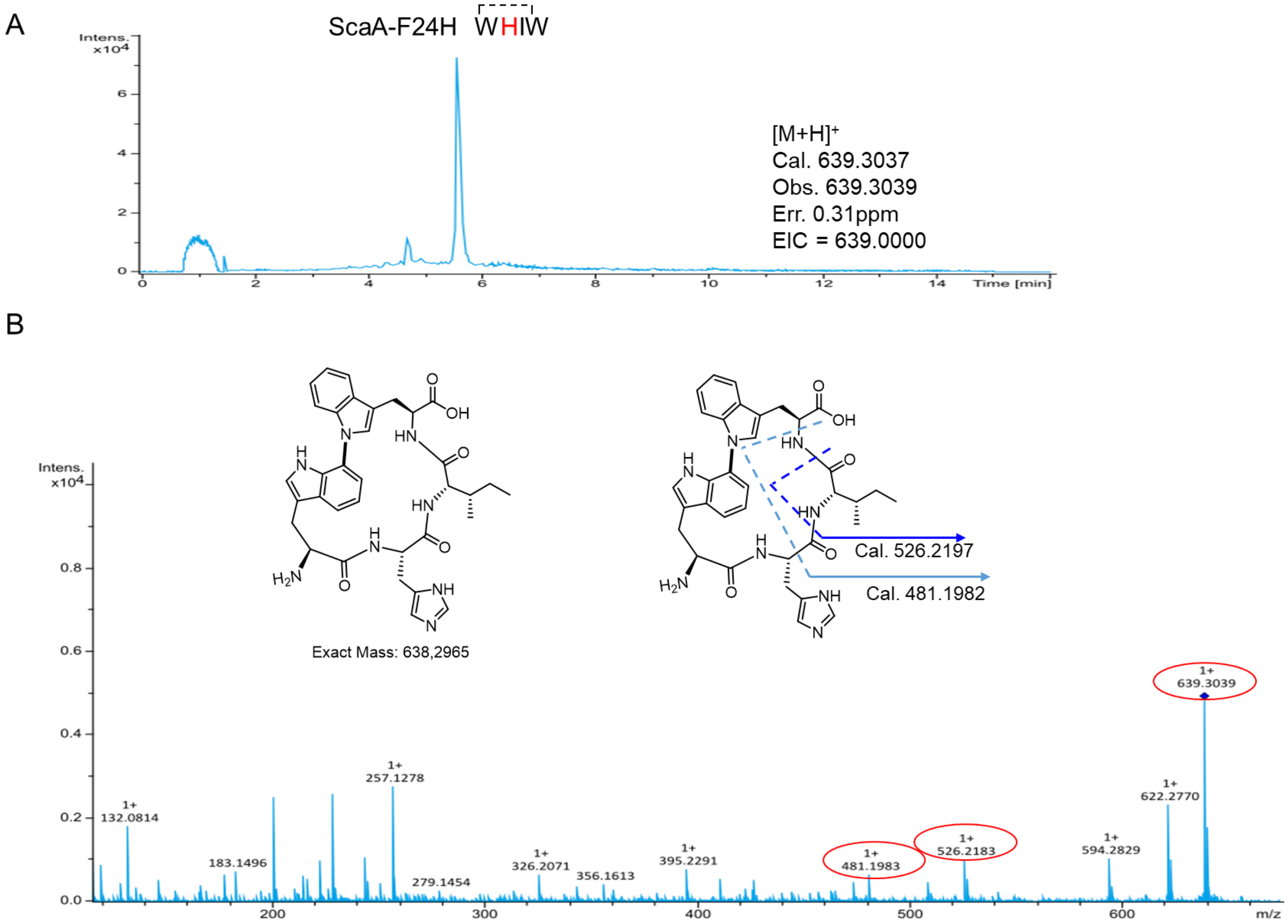

Figure S47. HRMS analysis of extracts of the recombinant *S. albus* strain harboring *scaA*-F24H and *scaB*. A) Extracted ion chromatogram of the product from the coexpression of *scaA*-F24H with *scaB*; B) MS/MS spectrum of the corresponding atropopeptide detected at *m*/*z* 639.3039 $[M+H]^+$ with key fragments that indicate the presence of a bond between the two Trp residues highlighted.

A

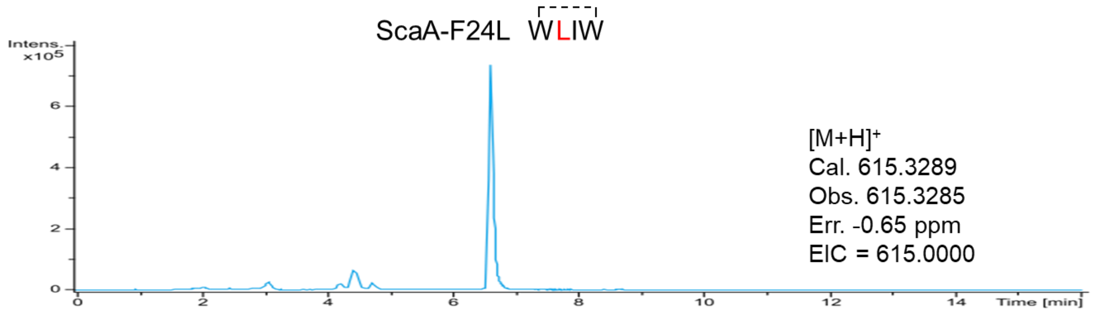

B

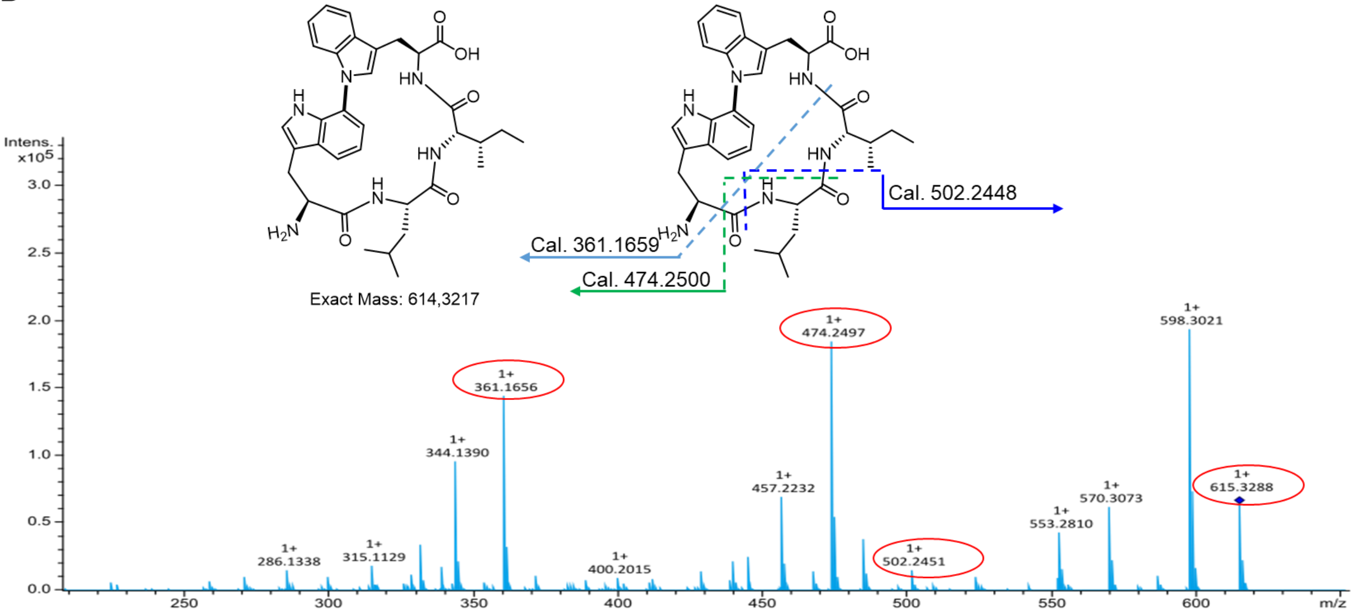

Figure S48. HRMS analysis of extracts of the recombinant *S. albus* strain harboring *scaA*-F24L and *scaB*. A) Extracted ion chromatogram of the product from the coexpression of *scaA*-F24L with *scaB*; B) MS/MS spectrum of the corresponding atropopeptide detected at *m*/*z* 615.3285 [M+H]+ with key fragments that indicate the presence of a bond between the two Trp residues highlighted.

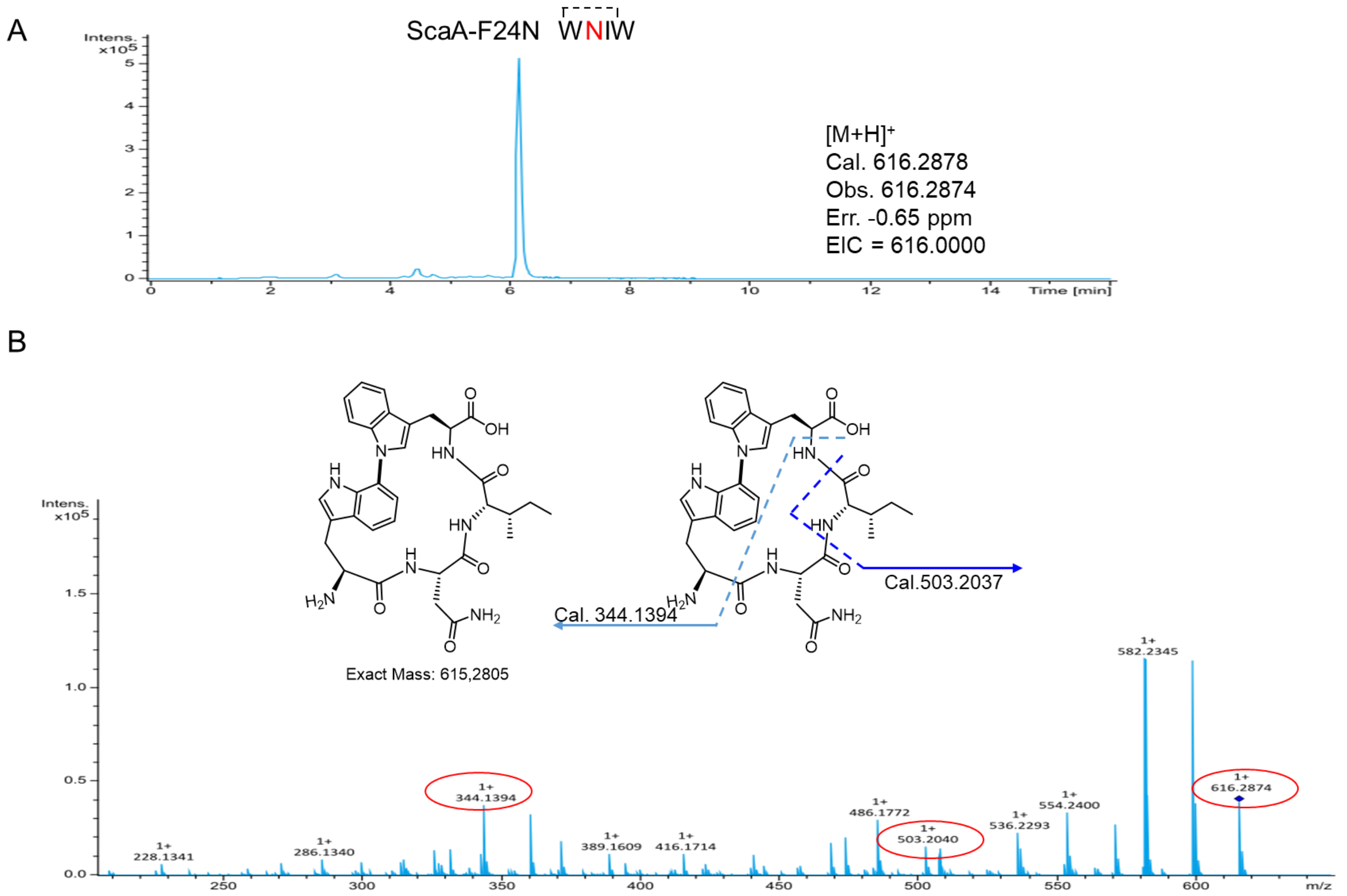

Figure S49. HRMS analysis of extracts of the recombinant *S. albus* strain harboring *scaA*-F24N and *scaB*. A) Extracted ion chromatogram of the product from the coexpression of *scaA*-F24N with *scaB*; B) MS/MS spectrum of the corresponding atropopeptide detected at *m*/*z* 616.2874 $[M+H]^+$ with key fragments that indicate the presence of a bond between the two Trp residues highlighted.

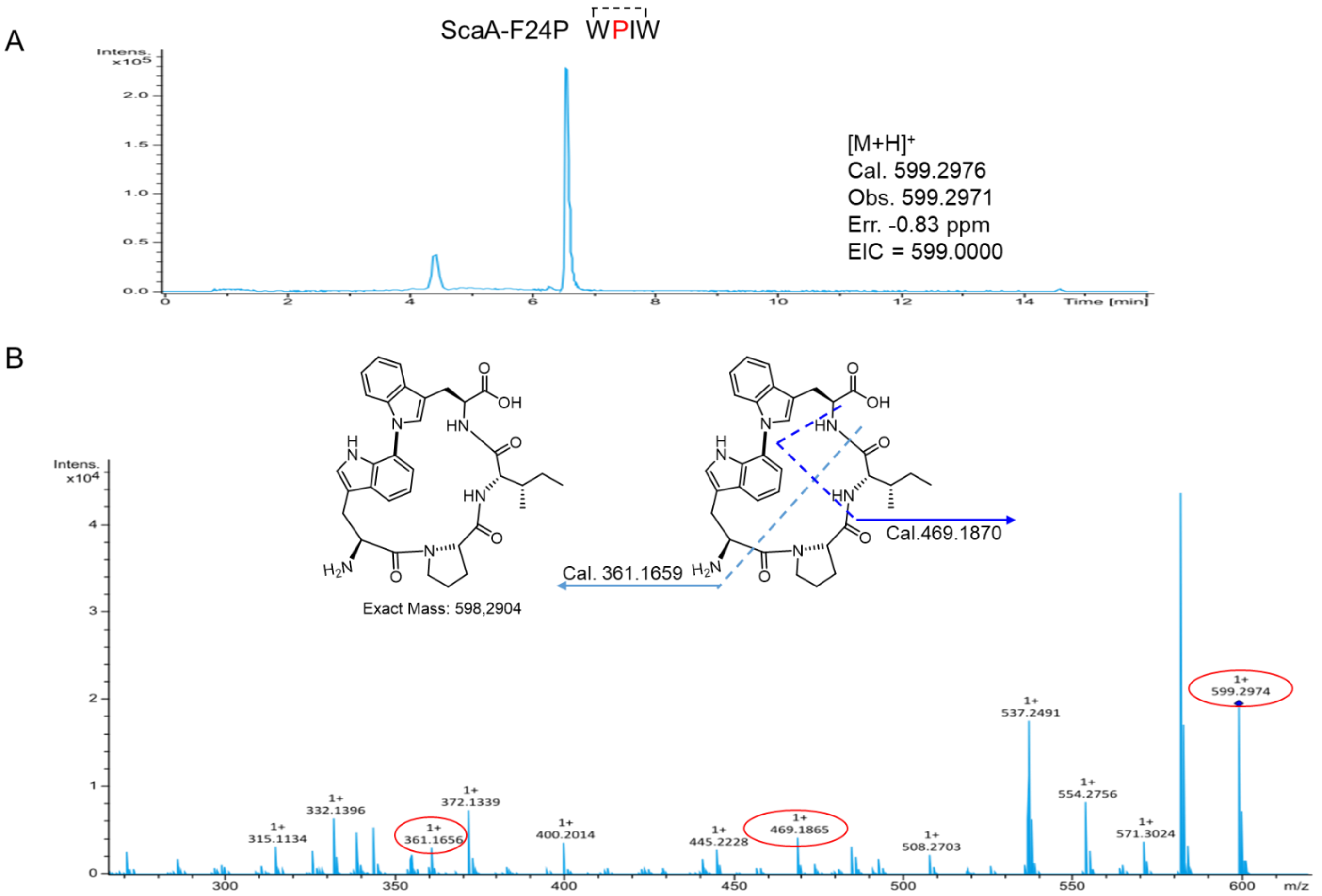

Figure S50. HRMS analysis of extracts of the recombinant *S. albus* strain harboring *scaA*-F24P and *scaB*. A) Extracted ion chromatogram of the product from the coexpression of *scaA*-F24P with *scaB*; B) MS/MS spectrum of the corresponding atropopeptide detected at *m*/*z* 599.2971 [M+H]+ with key fragments that indicate the presence of a bond between the two Trp residues highlighted.

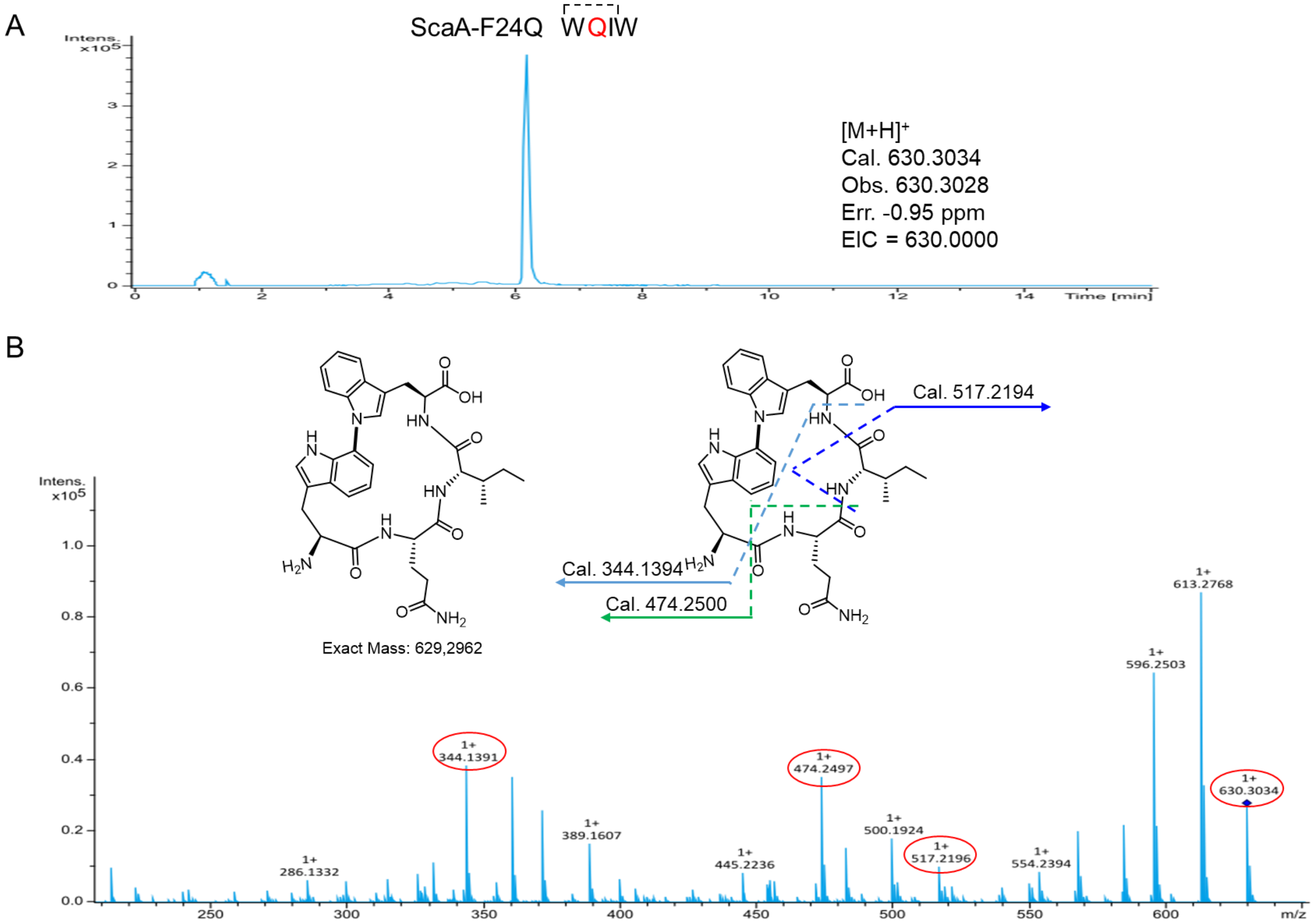

Figure S51. HRMS analysis of extracts of the recombinant *S. albus* strain harboring *scaA*-F24Q and *scaB*. A) Extracted ion chromatogram of the product from the coexpression of *scaA*-F24Q with *scaB*; B) MS/MS spectrum of the corresponding atropopeptide detected at *m*/*z* 630.3028 $[M+H]^+$ with key fragments that indicate the presence of a bond between the two Trp residues highlighted.

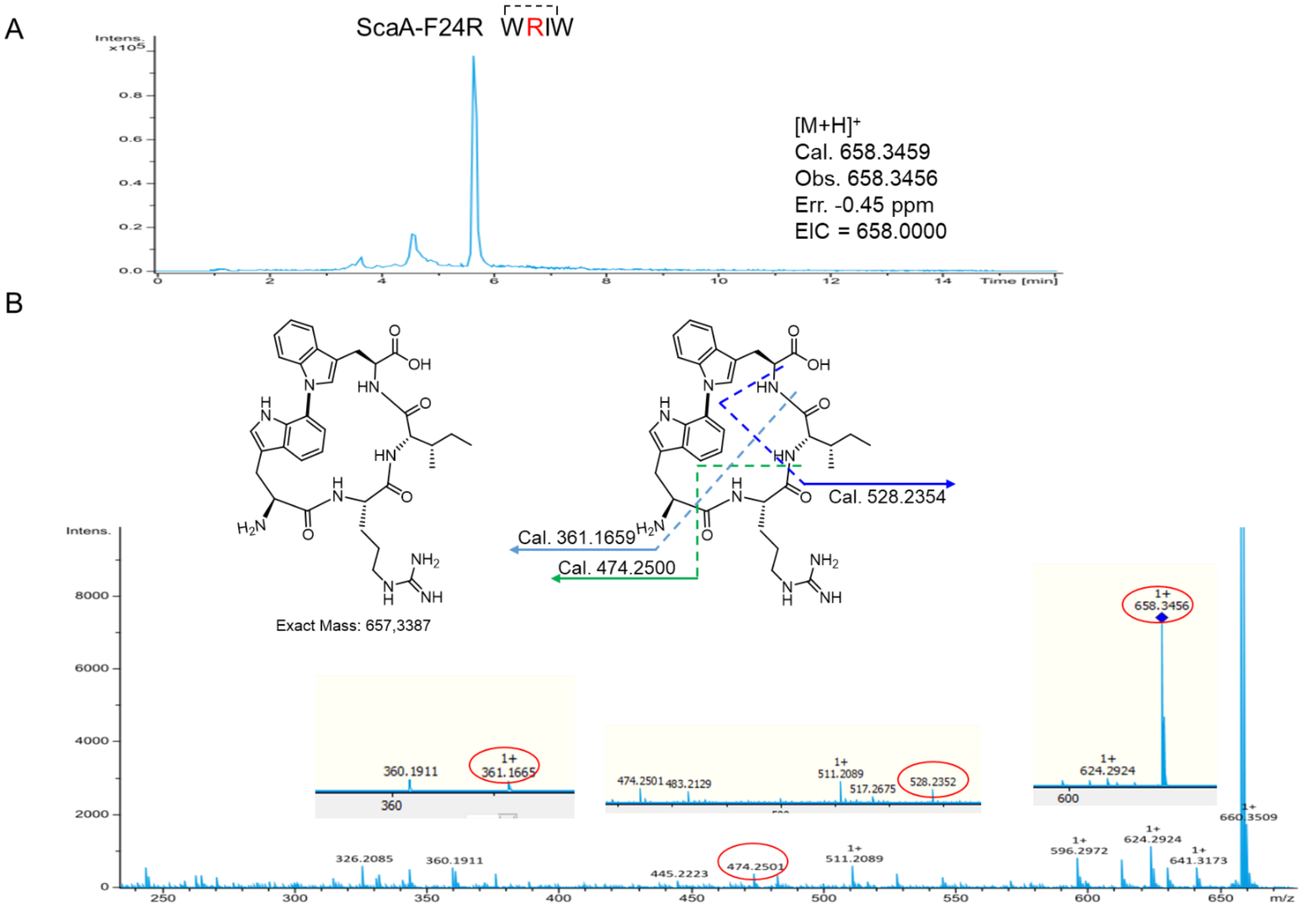

Figure S52. HRMS analysis of extracts of the recombinant *S. albus* strains harboring *scaA*-F24R and *scaB*. A) Extracted ion chromatogram of the product from the coexpression of *scaA*-F24R with *scaB*; B) MS/MS spectrum of the corresponding atropopeptide detected at *m*/*z* 658.3456 $[M+H]^+$ with key fragments that indicate the presence of a bond between the two Trp residues highlighted. The parent ion *m*/*z* 658.3456 $[M+H]^+$ and fragments with *m*/*z* 361 $[M+H]^+$,or *m*/*z* 528 $[M+H]^+$ can be observed in the magnified inserted regions.

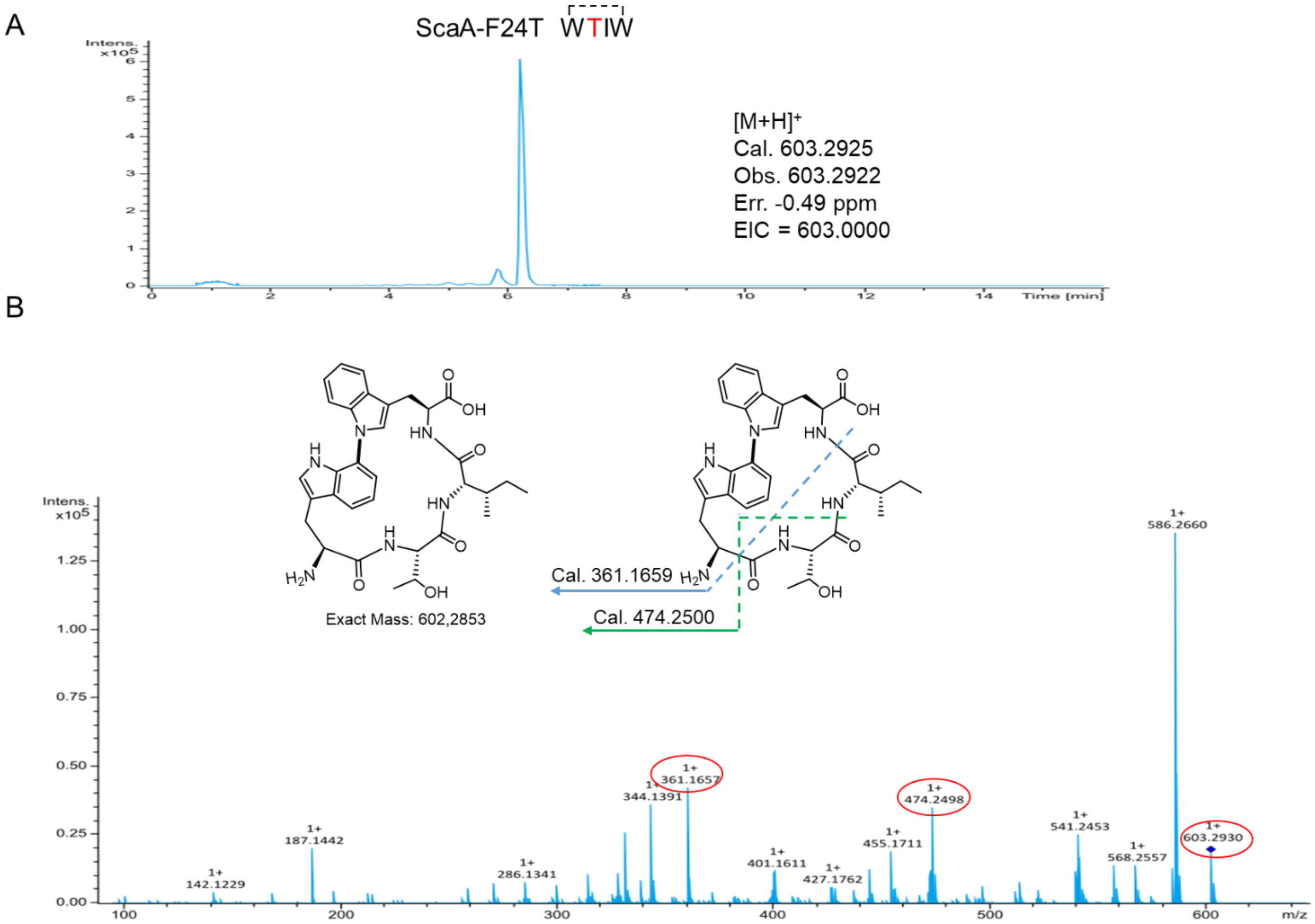

Figure S53. HRMS analysis of extracts of the recombinant *S. albus* strain harboring *scaA*-F24T and *scaB*. A) Extracted ion chromatogram of the product from the coexpression of *scaA*-F24T with *scaB*; B) MS/MS spectrum of the corresponding atropopeptide detected at *m*/*z* 603.2922 $[M+H]^+$ with key fragments that indicate the presence of a bond between the two Trp residues highlighted.

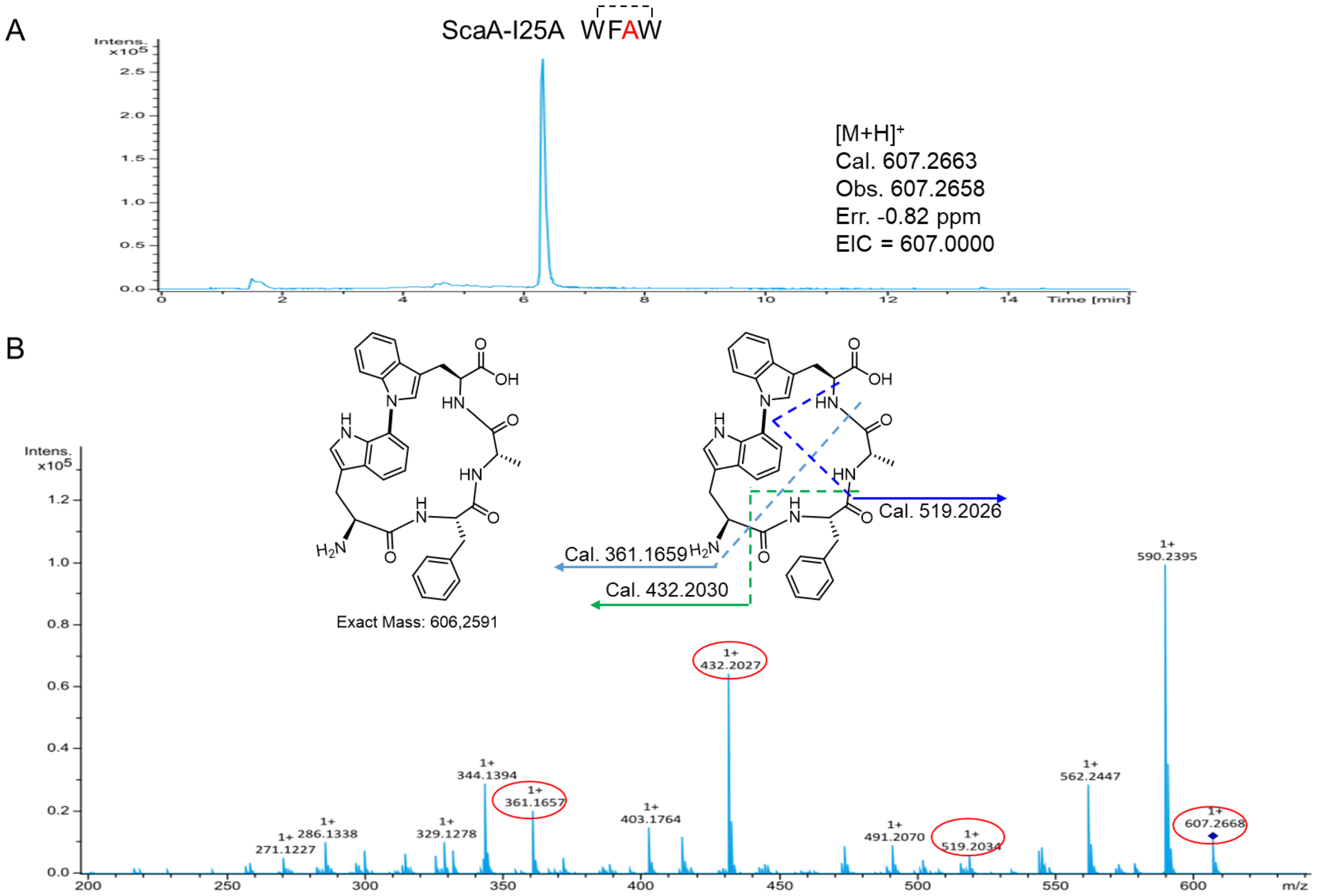

Figure S54. HRMS analysis of extracts of the recombinant *S. albus* strain harboring *scaA*-I25A and *scaB*. A) Extracted ion chromatogram of the product from the coexpression of *scaA*-I25A with *scaB*; B) MS/MS spectrum of the corresponding atropopeptide detected at *m*/*z* 607.2658[M+H]$^+$ with key fragments that indicate the presence of a bond between the two Trp residues highlighted.

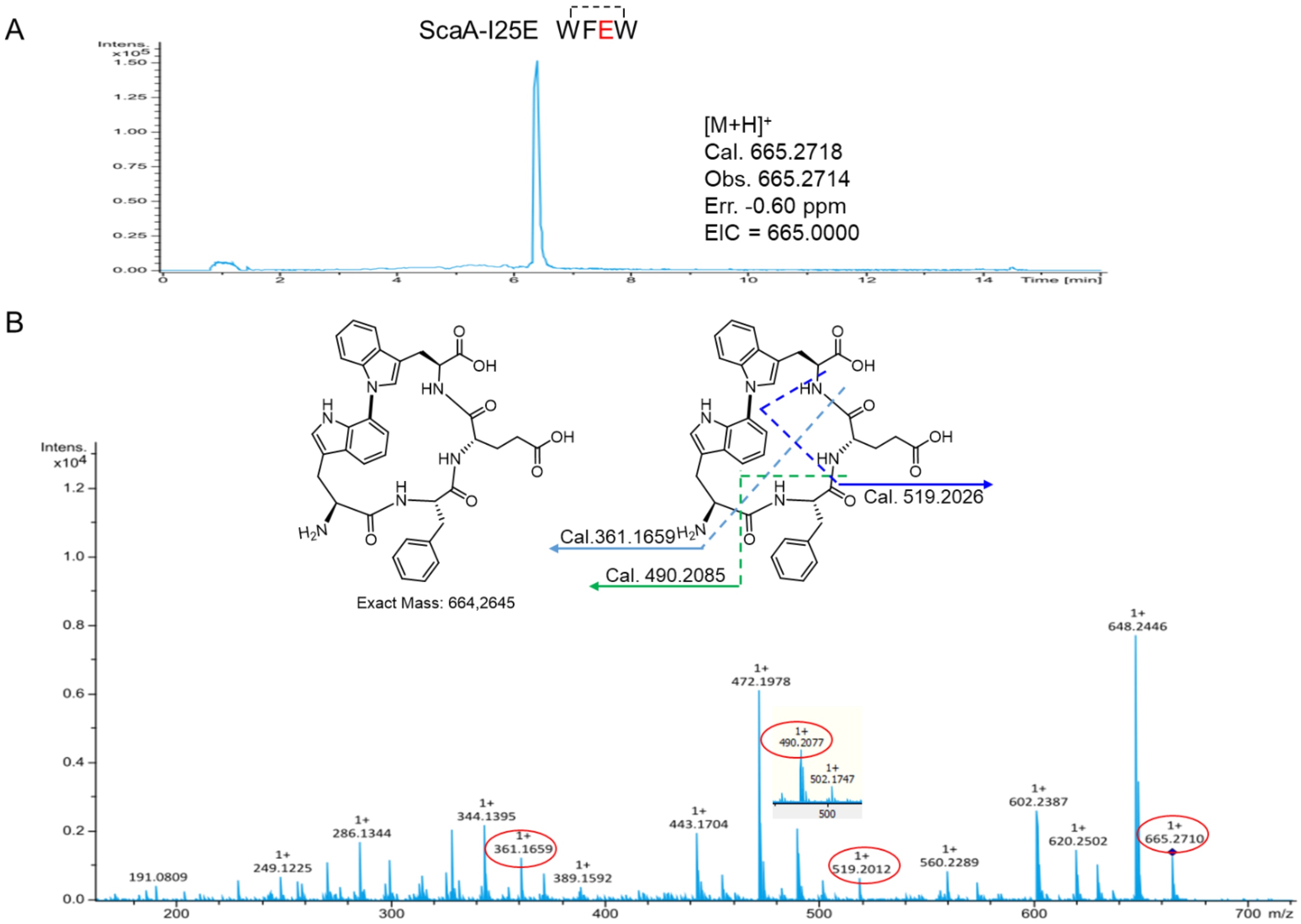

Figure S55. HRMS analysis of extracts of the recombinant *S. albus* strain harboring *scaA*-I25E and *scaB*. A) Extracted ion chromatogram of the product from the coexpression of *scaA*-I25E with *scaB*; B) MS/MS spectrum of the corresponding atropopeptide detected at *m*/*z* 665.2714[M+H]$^+$ with key fragments that indicate the presence of a bond between the two Trp residues highlighted. The fragment with *m*/*z* 490 [M+H]$^+$ can be observed in the magnified inserted region.

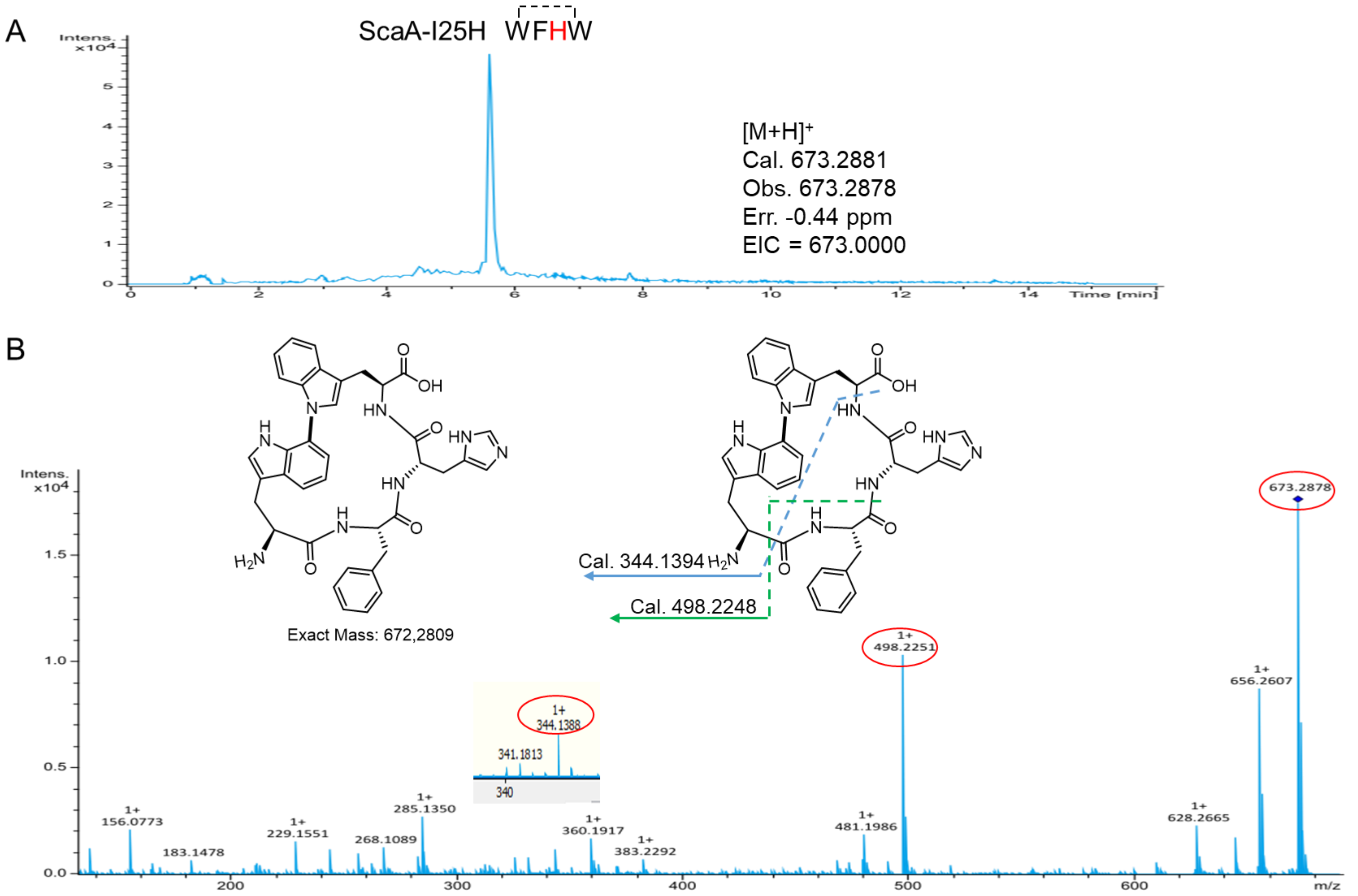

Figure S56. HRMS analysis of extracts of the recombinant *S. albus* strain harboring *scaA*-I25H and *scaB*. A) Extracted ion chromatogram of the product from the coexpression of *scaA*-I25H with *scaB*; B) MS/MS spectrum of the corresponding atropopeptide detected at *m*/*z* 673.2878 $[M+H]^+$ with key fragments that indicate the presence of a bond between the two Trp residues highlighted. The fragment with *m*/*z* 344 $[M+H]^+$ can be observed in the magnified inserted region.

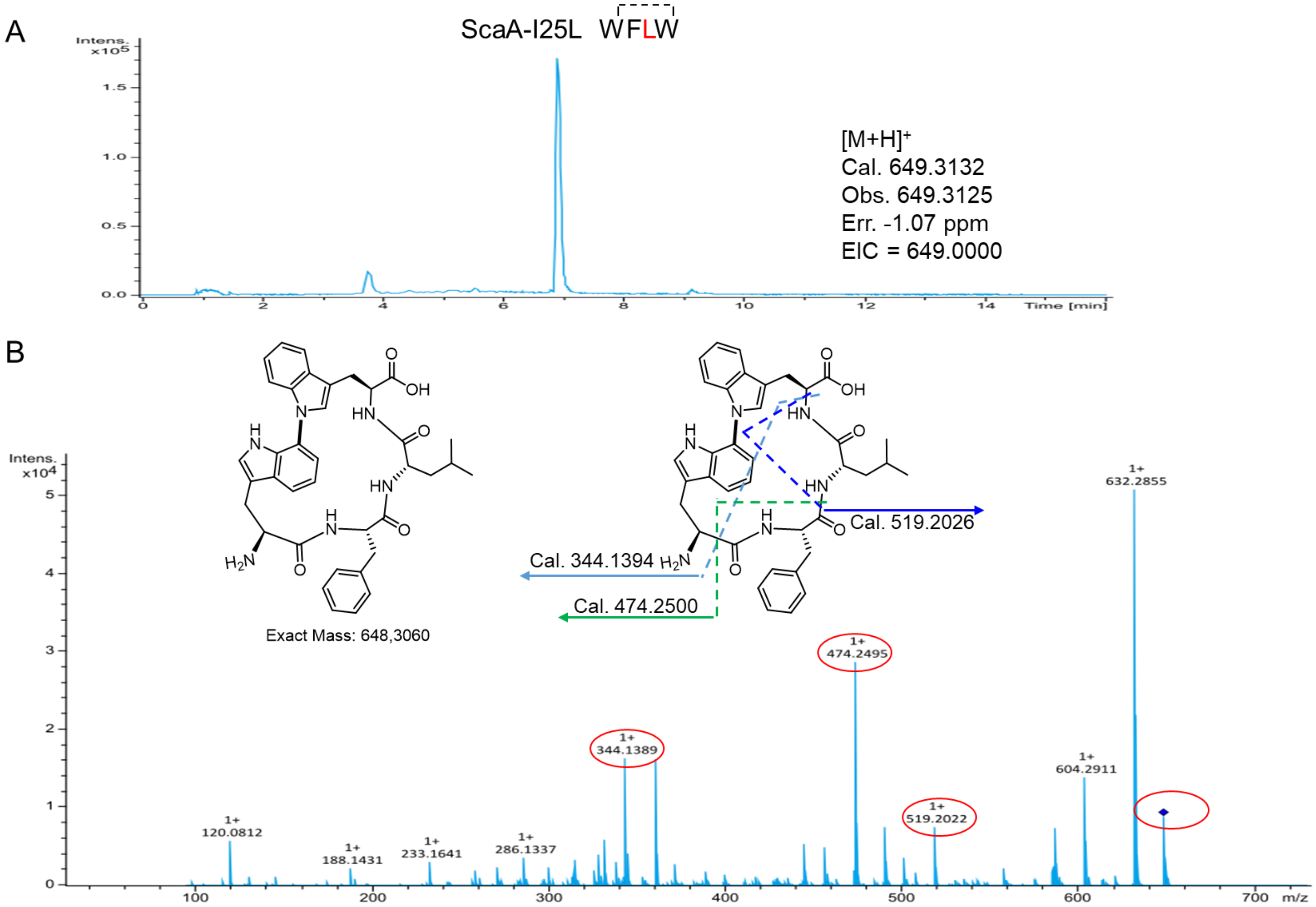

Figure S57. HRMS analysis of extracts of the recombinant *S. albus* strain harboring *scaA*-I25L and *scaB*. A) Extracted ion chromatogram of the product from the coexpression of *scaA*-I25L with *scaB*; B) MS/MS spectrum of the corresponding atropopeptide detected at *m*/*z* 649.3125 [M+H]$^+$ with key fragments that indicate the presence of a bond between the two Trp residues highlighted.

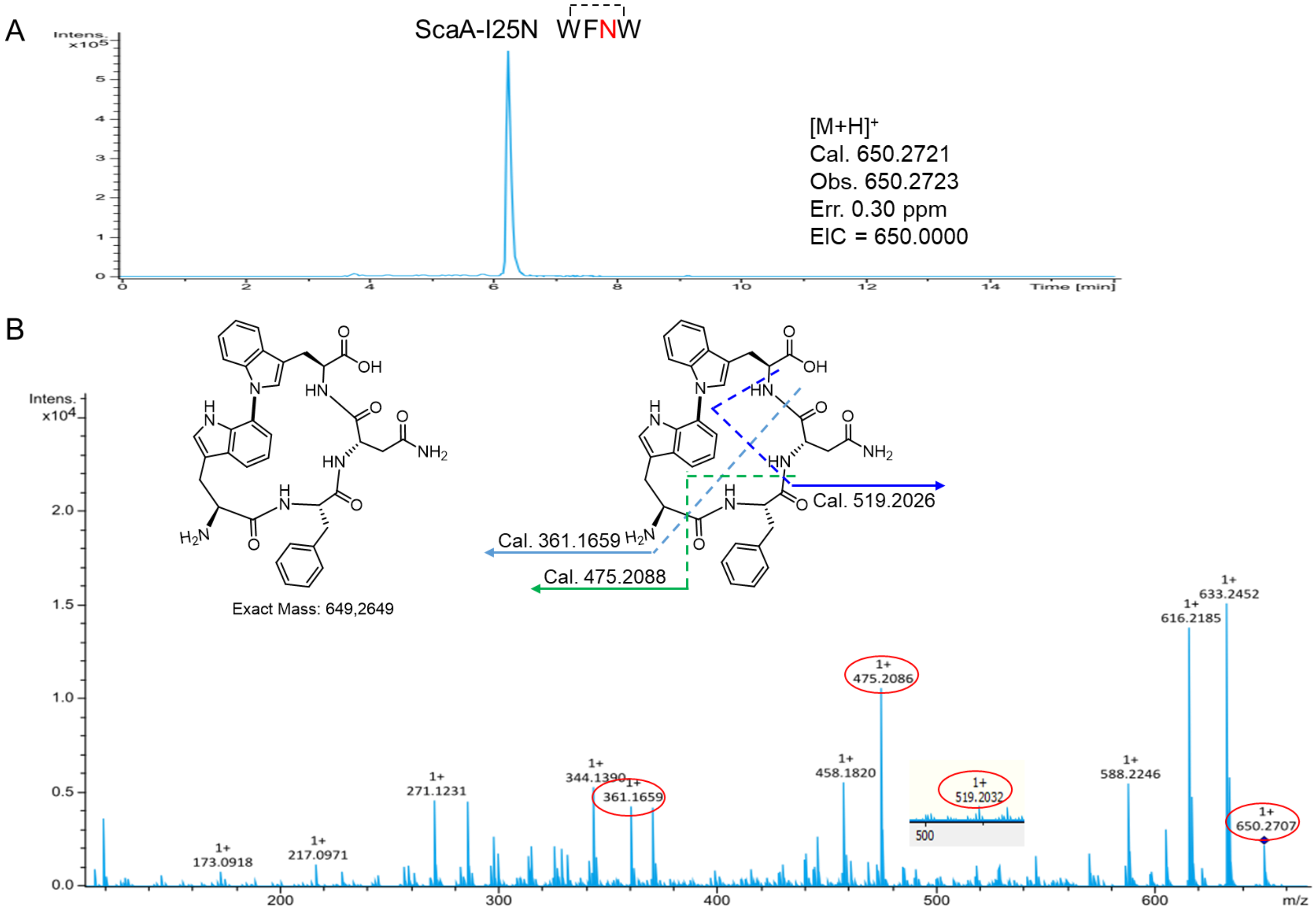

Figure S58. HRMS analysis of extracts of the recombinant *S. albus* strain harboring *scaA*-I25N and *scaB*. A) Extracted ion chromatogram of the product from the coexpression of *scaA*-I25N with *scaB*; B) MS/MS spectrum of the corresponding atropopeptide detected at *m*/*z* 650.2723 $[M+H]^+$ with key fragments that indicate the presence of a bond between the two Trp residues highlighted. The fragment with *m*/*z* 519 $[M+H]^+$ can be observed in the magnified inserted region.

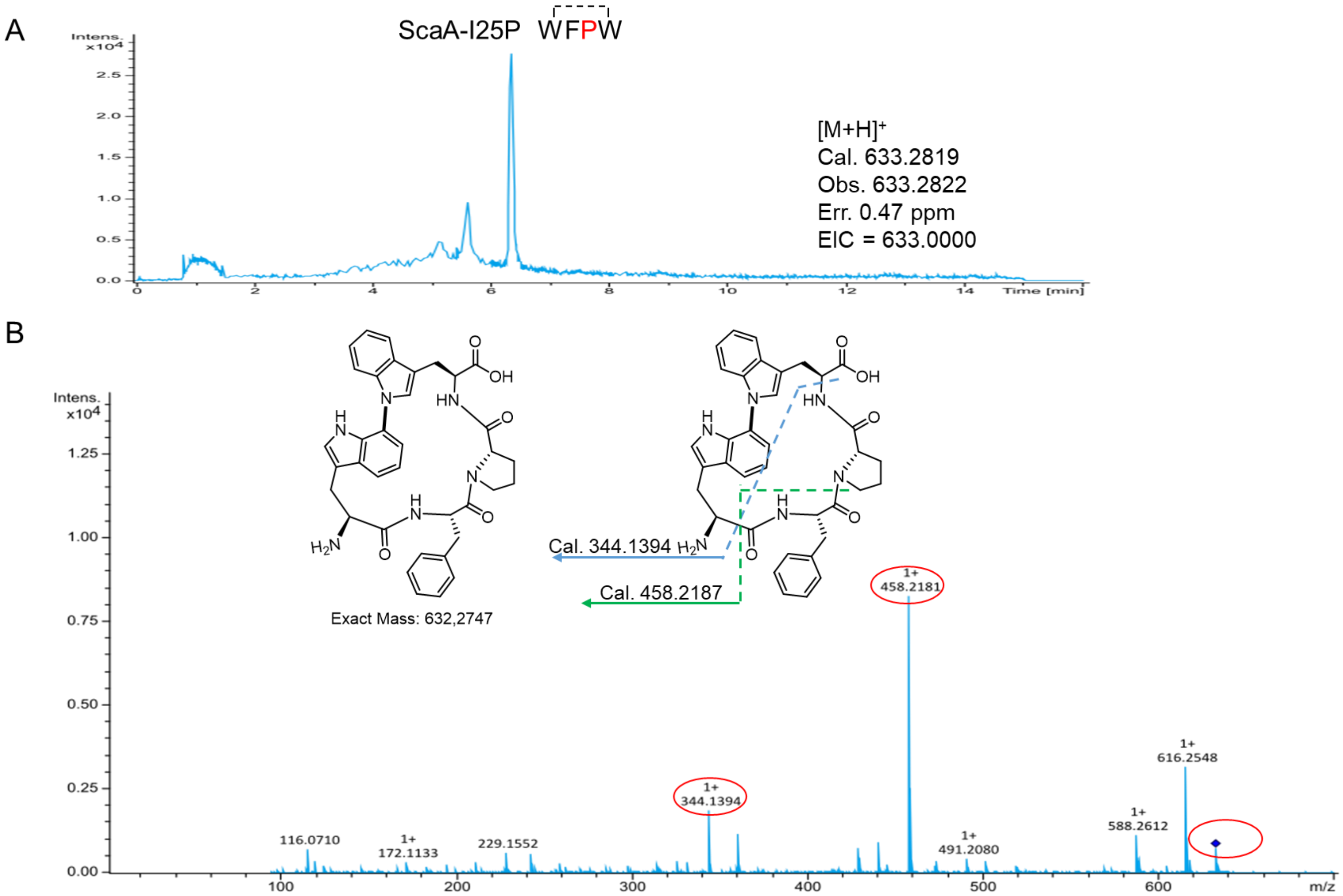

Figure S59. HRMS analysis of extracts of the recombinant *S. albus* strain harboring *scaA*-I25P and *scaB*. A) Extracted ion chromatogram of the product from the coexpression of *scaA*-I25P with *scaB*; B) MS/MS spectrum of the corresponding atropopeptide detected at *m*/*z* 633.2822 [M+H]$^+$ with key fragments that indicate the presence of a bond between the two Trp residues highlighted.

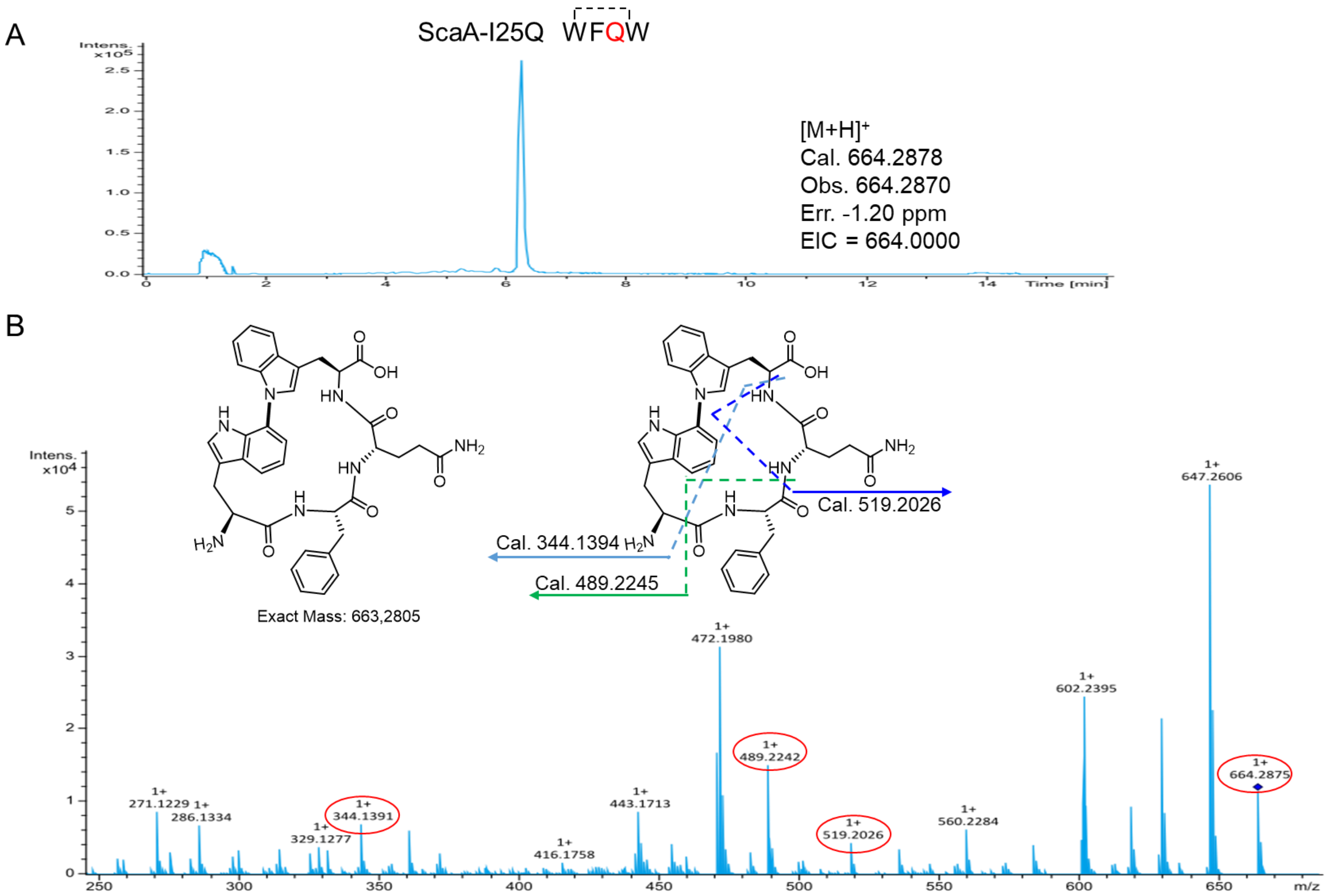

Figure S60. HRMS analysis of extracts of the recombinant *S. albus* strain harboring *scaA*-I25Q and *scaB*. A) Extracted ion chromatogram of the product from the coexpression of *scaA*-I25Q with *scaB*; B) MS/MS spectrum of the corresponding atropopeptide detected at *m*/*z* 664.2870 [M+H]$^+$ with key fragments that indicate the presence of a bond between the two Trp residues highlighted.

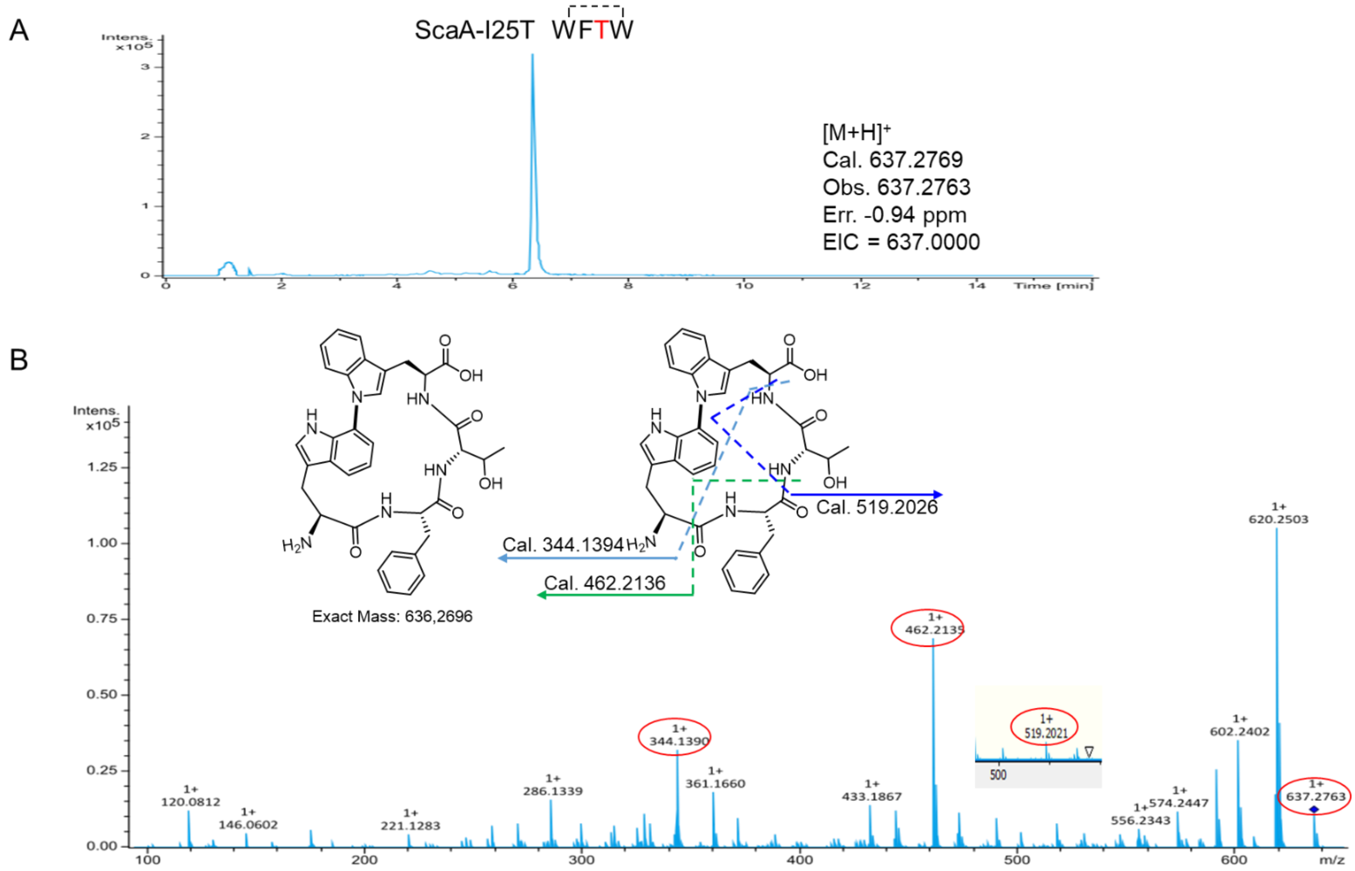

Figure S61. HRMS analysis of extracts of the recombinant *S. albus* strain harboring *scaA*-I25T and *scaB*. A) Extracted ion chromatogram of the product from the coexpression of *scaA*-I25T with *scaB*; B) MS/MS spectrum of the corresponding atropopeptide detected at *m*/*z* 637.2763 $[M+H]^+$ with key fragments that indicate the presence of a bond between the two Trp residues highlighted. The fragment with *m*/*z* 519 $[M+H]^+$ can be observed in the magnified inserted region.

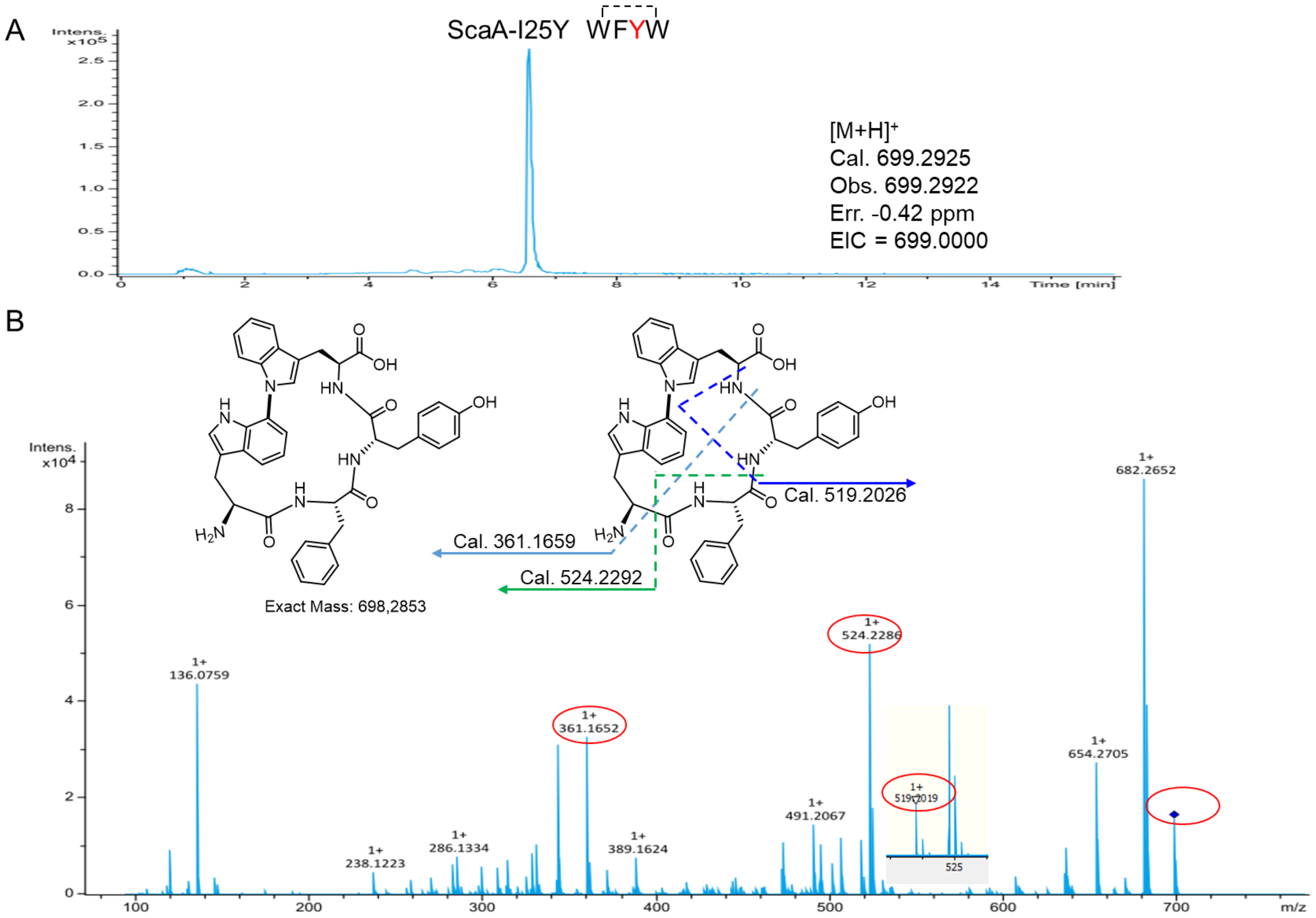

Figure S62. HRMS analysis of extracts of the recombinant *S. albus* strain harboring *scaA*-I25Y and *scaB*. A) Extracted ion chromatogram of the product from the coexpression of *scaA*-I25Y with *scaB*; B) MS/MS spectrum of the corresponding atropopeptide detected at *m*/*z* 699.2922 [M+H]+ with key fragments that indicate the presence of a bond between the two Trp residues highlighted. The fragment with *m*/*z* 519 [M+H]+ can be observed in the magnified inserted region.

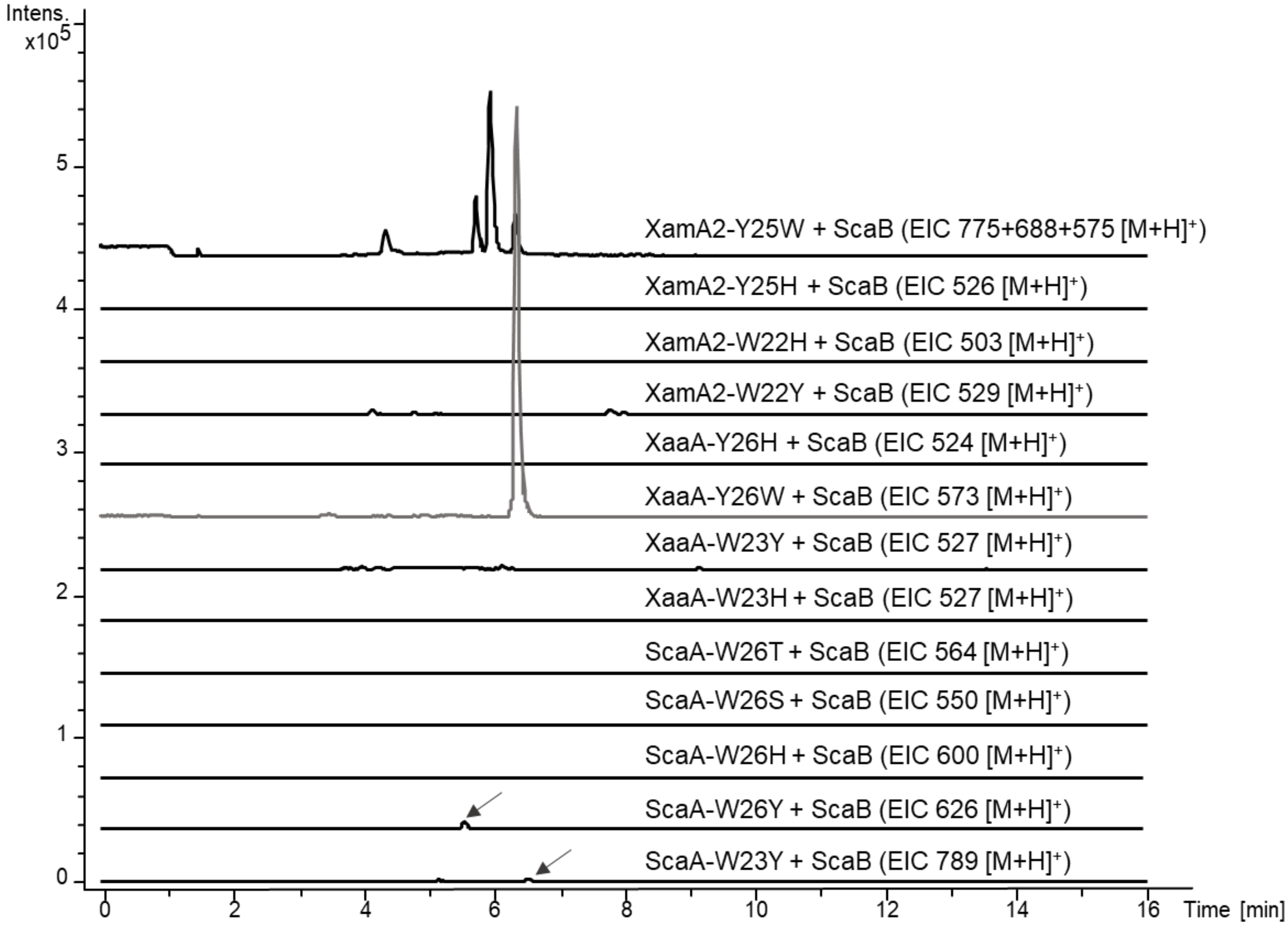

Figure S63. Effect of substitutions in crosslink-forming amino acid residues in XamA2, XaaA and ScaA precursors. Extracted ion chromatography of products generated from the coexpression of precursor variants with ScaB. The arrows indicate products in low yield.

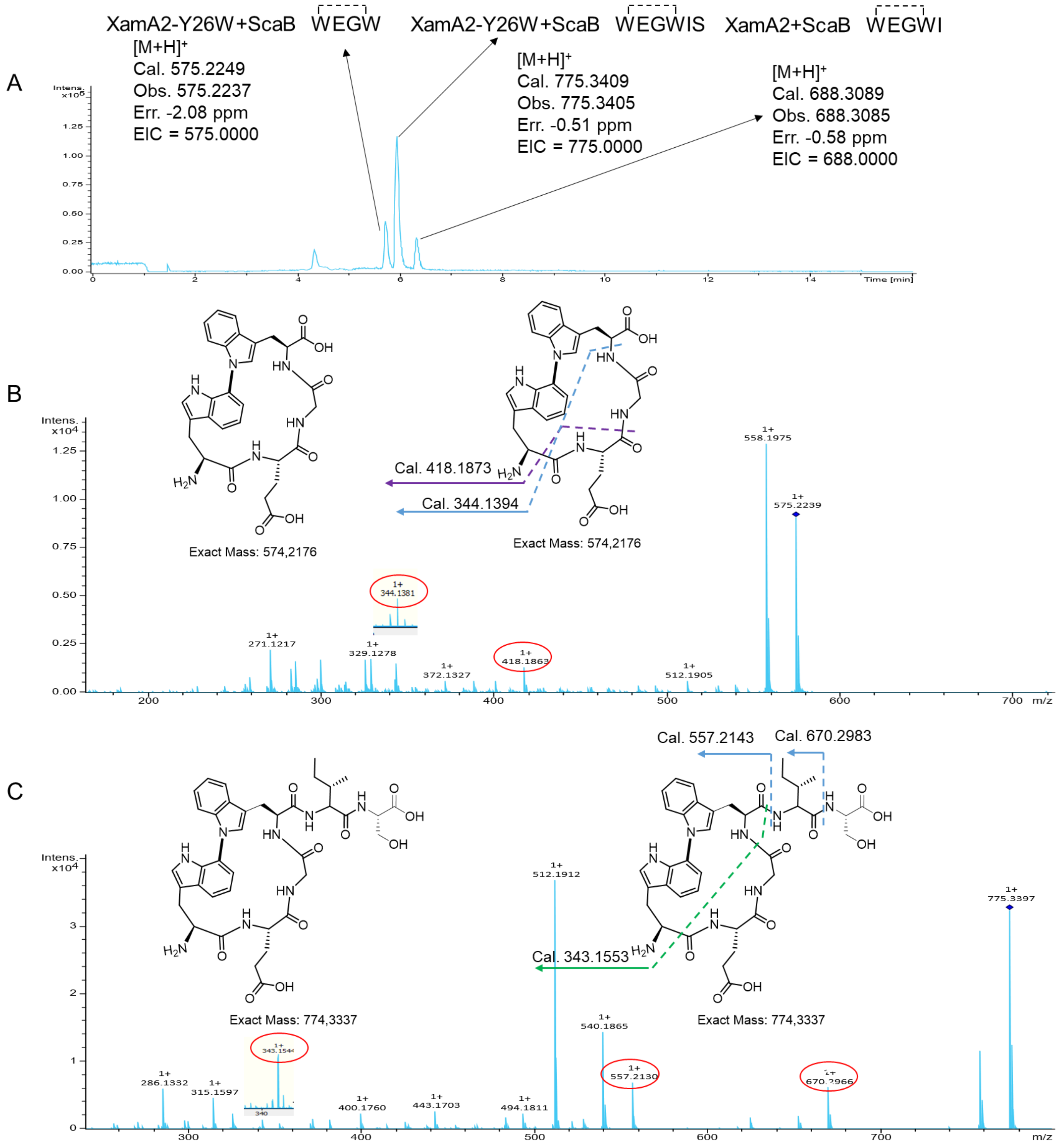

Figure S64. HRMS analysis of extracts of the recombinant *S. albus* strain harboring *xamA2*-Y25W and *scaB*. A) Extracted ion chromatogram of the product from the coexpression of *xamA2*-Y25W with *scaB*; B) MS/MS spectrum of the corresponding atropopeptide detected at *m*/*z* 575.2237 $[M+H]^+$ with key fragments that indicate the presence of a bond between Trp and Trp. The fragment with *m*/*z* 344.1381 $[M+H]^+$ can be observed in the magnified inserted region; C) MS/MS spectrum of the corresponding atropopeptide detected at *m*/*z* 775.3405 $[M+H]^+$ with key fragments that indicate the presence of a bond between Trp and Trp highlighted. The fragment with *m*/*z* 3443.1544 $[M+H]^+$ can be observed in the magnified inserted region.

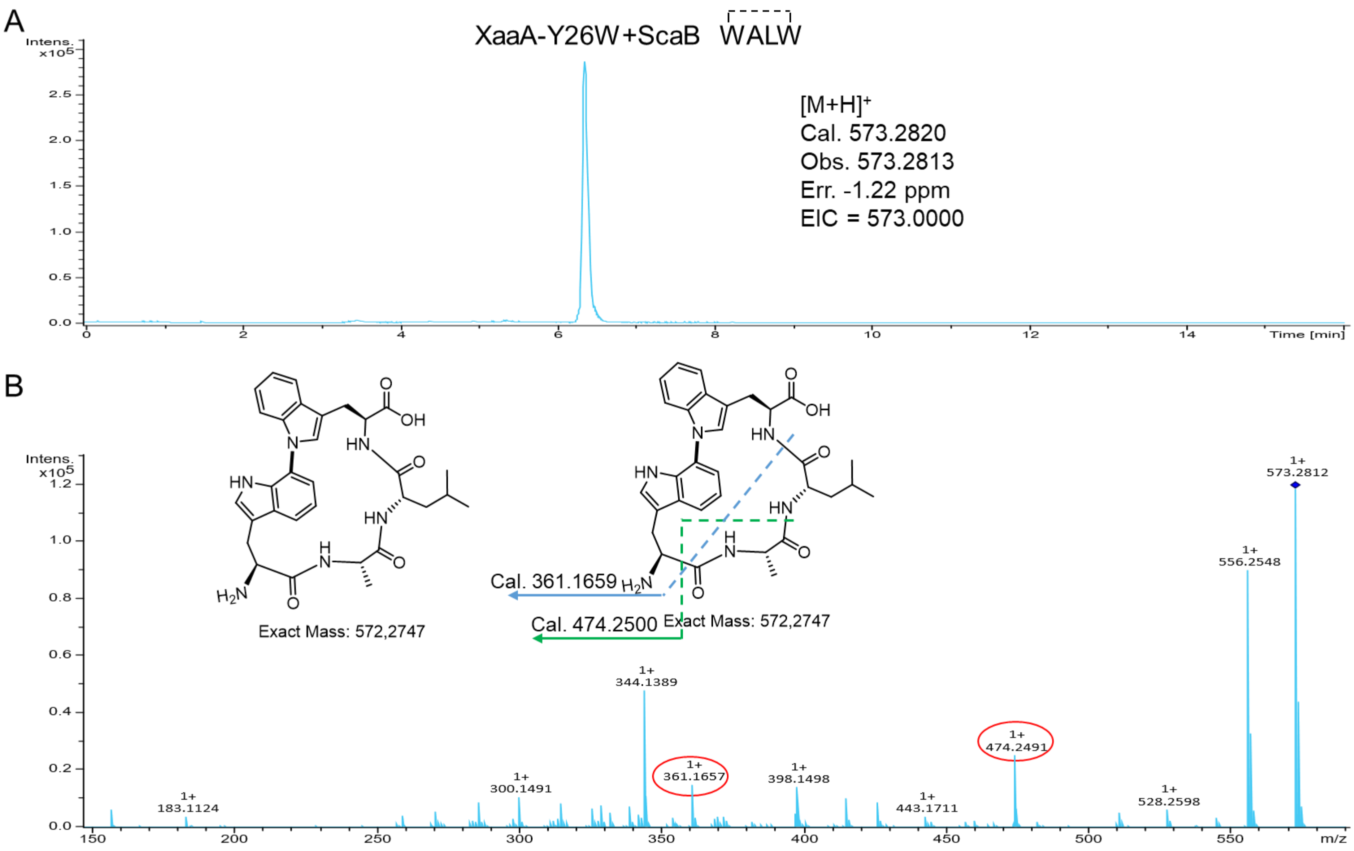

Figure S65. HRMS analysis of extracts of the recombinant *S. albus* strain harboring *xaaA*-Y26W and *scaB*. A) Extracted ion chromatogram of the product from the coexpression of *xaaA*-Y26W with *scaB*; B) MS/MS spectrum of the corresponding atropopeptide detected at *m*/*z* 573.2813 $[M+H]^+$ with key fragments that indicate the presence of a bond between Trp and Trp.

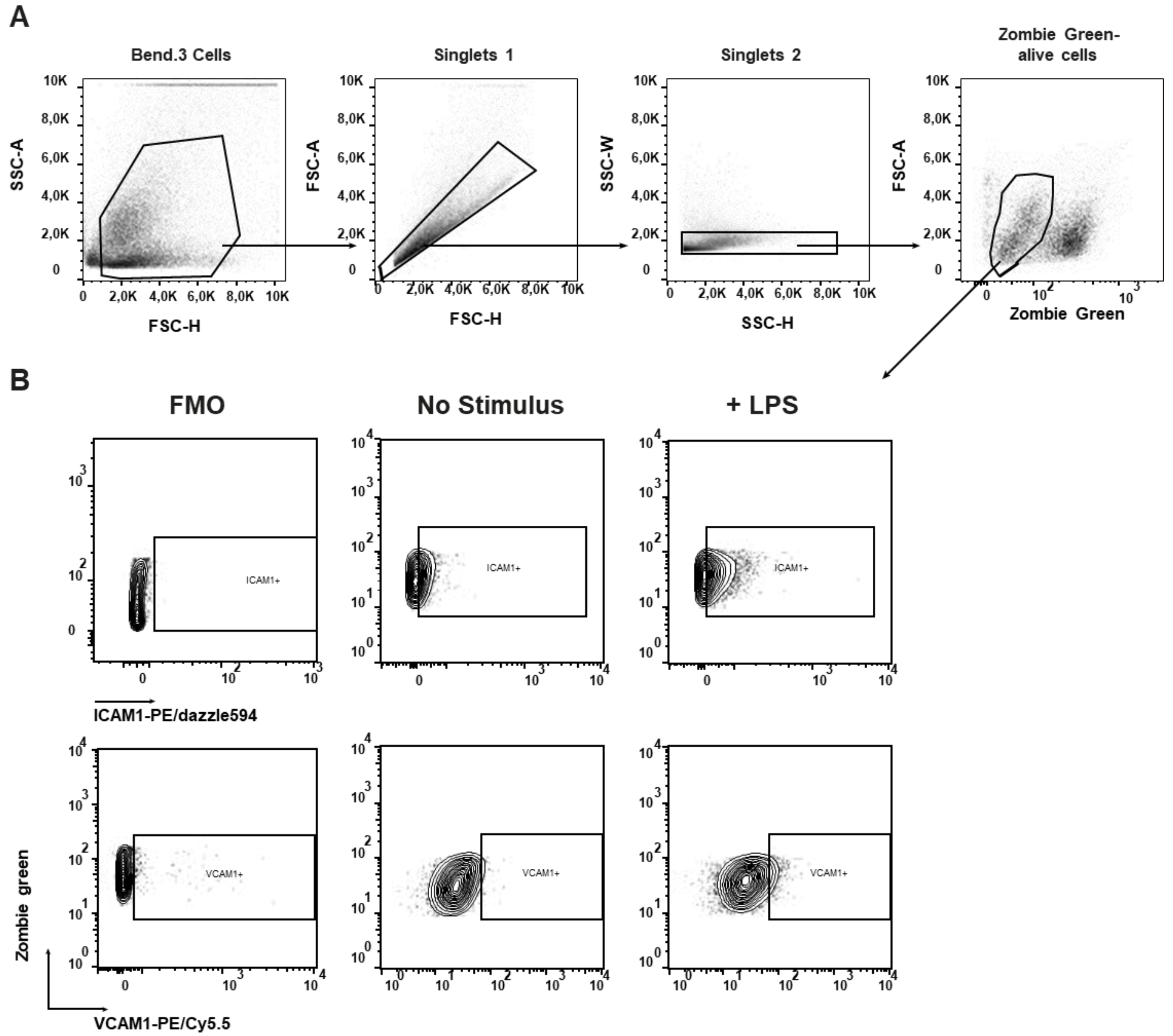

Figure S66. Gating strategy for flow cytometry analysis of bEnd.3 cell inflammation assay. Cells were discriminated from debris and larger clumps employing front scatter versus side scatter morphology plots. This was followed by single cell selection via two sequential doublet exclusion gates and subsequent exclusion of dead- or damaged ZombieGreen^{TM+} cells (A). Positivity for the inflammatory markers ICAM1 and VCAM1 in alive single cells was then determined using fluorescence minus one (FMO) controls for each respective marker (B).

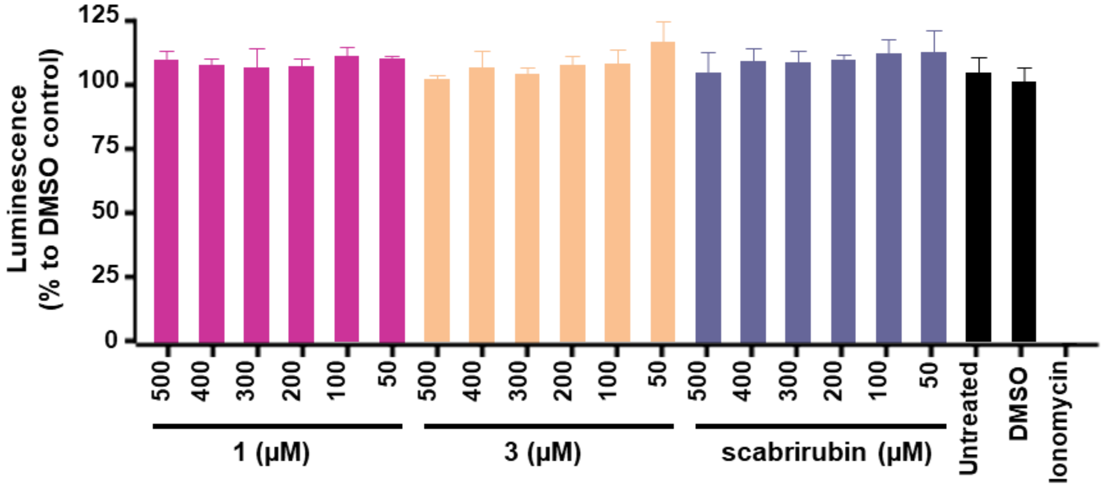

Figure S67. Effect of atropopeptides on the viability of MDCK II cells. Cell viability was assessed by measuring adenosine triphosphate (ATP) levels using the CellTiter-Glo assay after the exposure of MDCK II cells to atropopeptides for 48 h. Cell viability was normalized to the dimethyl sulfoxide (DMSO) control, which was set to 100%.

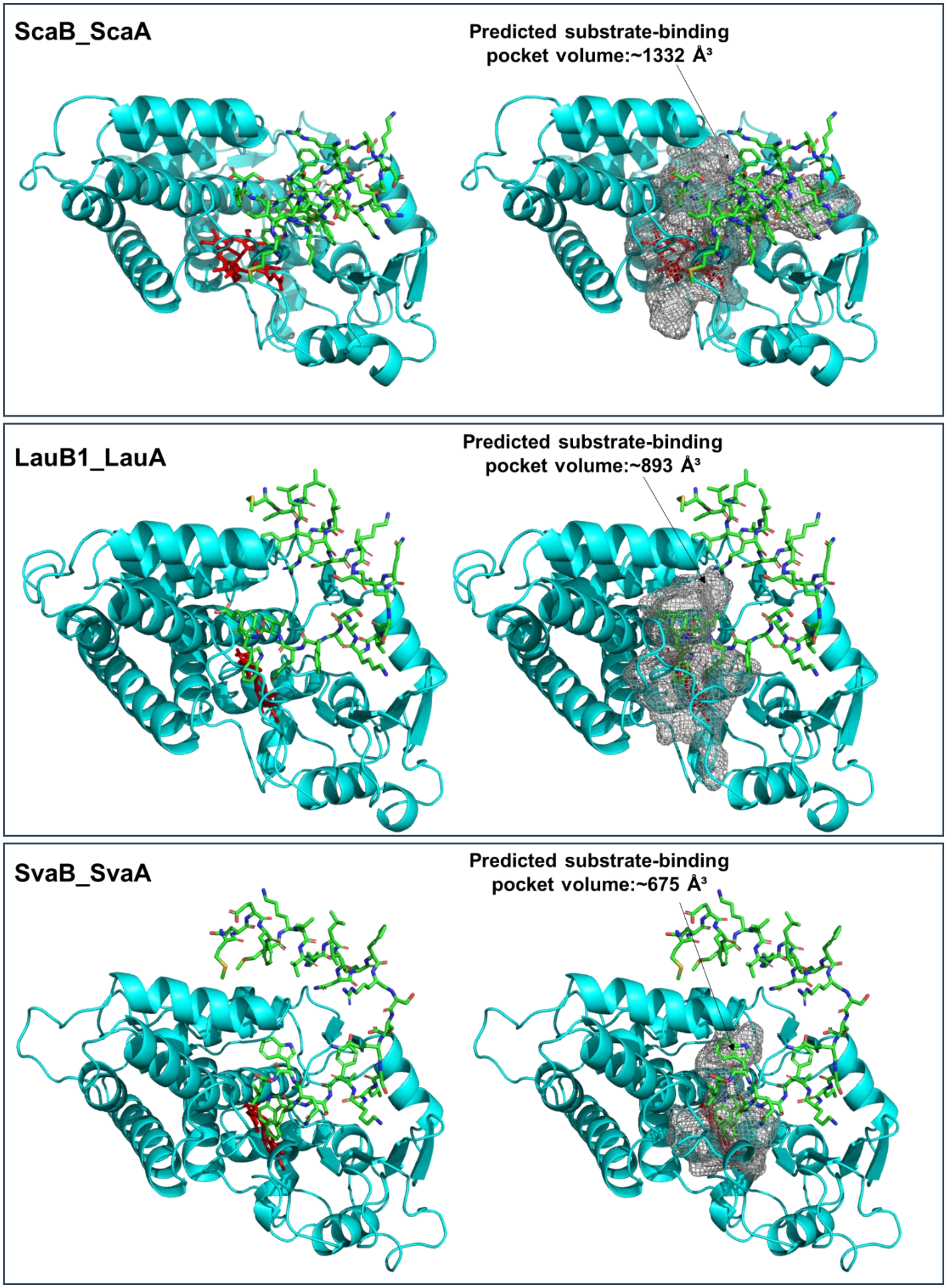

Figure S68. Predicted structural models of ScaB, LauB1 and SvaB and their precursor peptides. The predicted substrate-binding pockets are displayed as grey mesh.

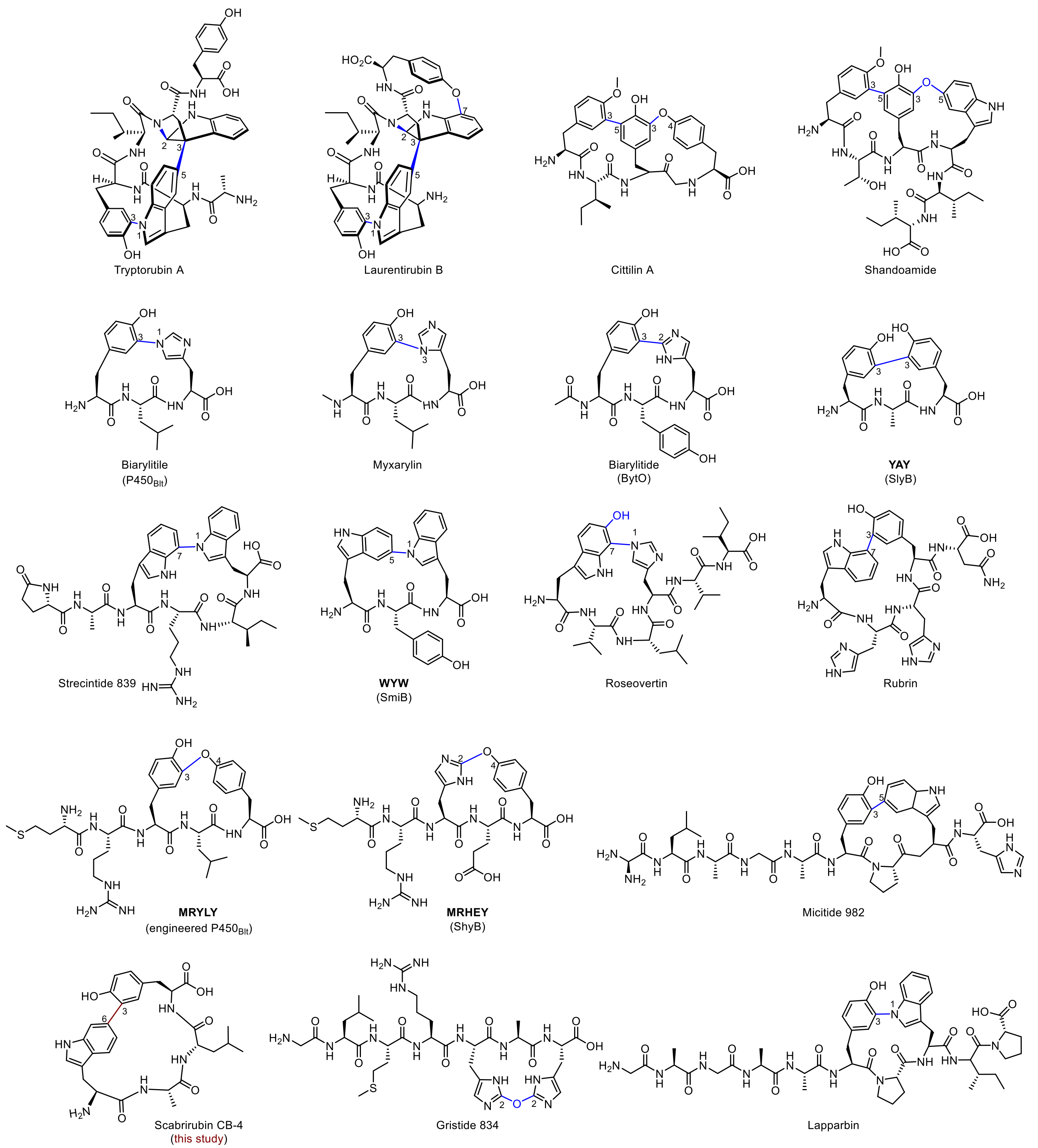

Figure S69. Representative macrocycles installed by P450s in RiPP biosynthesis. One representative RiPP for each type of crosslink is shown with the crosslink-forming atoms numbered.

Table S1. Calculated mass for linear core peptide, monocyclic peptide and observed mass for monocyclic product

Atropopeptide core peptide	Calculated mass for monocyclic peptide $[M+H]^+$	Calculated mass for monocyclic peptide $[M+H]^+$	Observed mass for monocyclic peptide $[M+H]^+$	Δppm
SvaA(WYQWL)	795.3824	793.3667	793.3662	-0.63
NouA(WYGW)	611.2612	609.2456	609.2454	0.32
LauA(WYIW)	667.3238	665.3082	665.3080	-0.60
KatA(WYFW)	701.3082	699.2925	699.2925	0.00
JumA(WYIW)	667.3238	665.3082	665.3079	-0.45
LucA(WYLW)	667.3238	665.3082	665.3086	0.60
CapA(WYLW)	667.3238	665.3082	665.3080	-0.30
SkaA(WYHW)	691.2987	689.2830	689.2830	-0.58
XatA(WLHW)	641.3194	639.3037	639.3037	0.00
XaaA(WALY)	552.2816	550.2660	550.2655	-0.90
XamA1(WPHWY)	788.3514	786.3430	786.3424	-0.76
XamA2(WEGYIS)	667.3086; 754.3406[a]	665.2929; 752.3249	665.2929; 752.3248	0.60; -0.13
XavA(WGLY)	538.2660	536.2503	536.2505	0.37
XvpA(WTEYF)	598.2507; 745.3191 [b]	596.2351; 743.3035	/	/
XvvA(WPEYF)	594.2558; 741.3242 [c]	592.2402; 739.3086	/	/
ScaA-F24A(WAIW)	575.2976	573.2819	573.2815	-0.69
ScaA-F24E(WEIW)	633.3031	631.2874	631.2869	-0.79
ScaA-F24H(WHIW)	641.3194	639.3037	639.3039	0.31
ScaA-F24L(WLIW)	617.3445	615.3289	615.3285	-0.65
ScaA-F24N(WNIW)	618.3034	616.2878	616.2874	-0.65
ScaA-F24P(WPIW)	601.3133	599.2976	599.2971	-0.83
ScaA-F24Q(WQIW)	632.3191	630.3034	630.3028	-0.95
ScaA-F24R(WRIW)	660.3616	658.3459	658.3456	-0.45
ScaA-F24T(WTIW)	605.3082	603.2925	603.2922	-0.49
ScaA-I25A(WFAW)	609.2820	607.2663	607.2658	-0.82
ScaA-I25E(WFEW)	667.2874	665.2718	665.2714	-0.60
ScaA-I25H(WFHW)	675.3038	673.2881	673.2878	-0.44
ScaA-I25L(WFLW)	651.3289	649.3132	649.3132	-1.07
ScaA-I25N(WFNW)	652.2878	650.2721	650.2723	0.30
ScaA-I25P(WFPW)	635.2976	633.2819	633.2822	0.47
ScaA-I25Q(WFQW)	666.3034	664.2878	664.2870	-1.20
ScaA-I25R(WFRW)	694.3460	692.3303	/	/
ScaA-I25T(WFTW)	639.2926	637.2769	637.2763	-0.94
ScaA-I25Y(WFYW)	701.3082	699.2925	699.2922	-0.42

[a]Two calculated mass means two possibilities. One is for pentapeptide (WEGYI), another one is for hexapeptide (WEGYIS).
[b]Two calculated mass means two possibilities. One is for tetrapeptide (WTEY), another one is for pentapeptide (WTEYF).
[c]Two calculated mass means two possibilities. One is for tetrapeptide (WPEY), another one is for pentapeptide (WPEYF).

Table S2. NMR data of scabrirubin CB-1 (**1**)

position	δ_H, (mult, J in Hz)	δ_C, type
1		173.7, C
2	4.38, m	52.8, CH
2-NH	7.99, d (6.0)	
3	3.24, m	27.0, CH_2
	3.13, dd (15.6; 9.6)	
4		112.1, C
5	7.79, s	128.1, CH
6		128.4, C
7	7.66, d (7.8)	118.9, CH
8	7.11, t (7.8)	119.5, CH
9	7.18, t (7.8)	122.0, CH
10	7.50, d (7.8)	109.6, CH
11		135.6, C
12		170.4, C
13	4.11, m	57.8, CH
13-NH	7.69, br s	
14	1.69, m	36.2, CH
15	1.62, m	25.2, CH_2
	1.35, m	
16	0.90, t (7.2)	11.0, CH_3
17	0.93, d (7.2)	15.1, CH_3
18		170.6, C
19	4.46, td (9.6;4.2)	53.3, CH
19-NH	7.35, d (9.6)	
20	2.57, dd (13.8; 4.2)	36.8, CH_2
	2.46, dd (13.8; 9.6)	
21		128.4, C
22/22′	7.02, d (8.4)	130.1, CH
23/23′	6.60, d (8.4)	114.7, CH
24		155.6, C
25		173.5, C
26	3.36, dd (12.6; 3.6)	56.2, CH
27	2.94, dd (12.6; 3.6)	31.7, CH_2
	2.74, t (12.6)	
28		110.6, C
29	6.72, br s	124.4, CH
29-NH	10.36, s	
30		129.3, C
31	7.56, d (7.8)	117.0, CH
32	7.14, m	118.3, CH
33	7.22, d (7.8)	116.2, CH
34		123.9, C
35		131.5, C

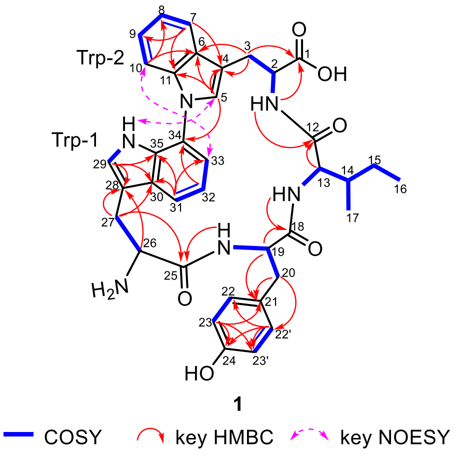

1

Table S3. NMR data of scabrirubin CB-2 (**2**)

position	δ_H, (mult, *J* in Hz)	δ_C, type
1		171.9, C
2	4.05, m	54.2, CH
2-NH	7.59, s	
3	3.03, dd (13.8; 5.4)	35.5, CH_2
	2.91, m	
4		128.1, C
5/5′	7.00, d (8.4)	130.0, CH
6/6′	6.62, d (8.4)	113.9, CH
7		155.0, C
8		170.1, C
9	4.38, m	52.6, CH
9-NH	7.92, s	
10	3.14, d (15.6)	27.1, CH_2
	2.92, m	
11		111.2, C
12	7.83, s	127.1, CH
13		127.9, C
14	7.61, d (7.8)	118.1, CH
15	7.15, m	119.1, CH
16	7.20, t (7.8)	121.6, CH
17	7.51, d (7.8)	109.1, CH
18		135.0, C
19		170.7, C
20	4.05, dd (6.0; 3.6)	57.4, CH
20-NH	7.75, s	
21	1.68, m	35.2, CH
22	1.58, m	24.4, CH_2
	1.31, m	
23	0.89, t (7.2)	10.5, CH_3
24	0.93, d (7.2)	14.9, CH_3
25		170.2, C
26	4.47, td (9.6; 4.2)	52.4, CH
26-NH	7.06, br d (9.6)	
27	2.52, m	36.1, CH_2
	2.40, dd (13.8; 9.6)	
28		127.7, C
29/29′	7.02, d (8.4)	129.6, CH
30/30′	6.59, d (8.4)	114.1, CH
31		155.1, C
32		173.1, C
33	3.34, m	55.7, CH
34	2.93, m	31.8, CH_2
	2.70,t (12.0)	
35		110.2, C
36	6.63, s	123.6, CH
36-NH	10.29, s	
37		128.11, C
38	7.56, d (7.8)	116.5, CH
39	7.14, m	117.7, CH
40	7.23, d (7.8)	115.8, CH
41		123.3, C
42		131.1, C

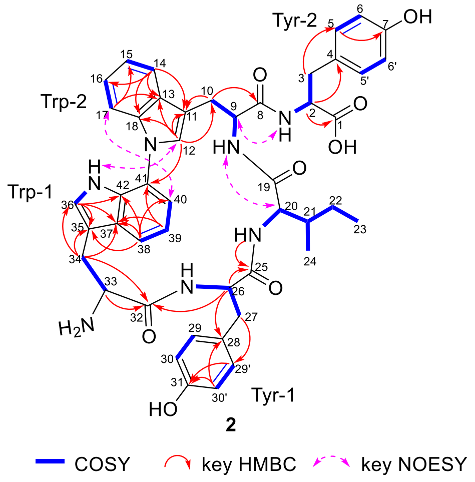

Table S4. NMR data of scabrirubin CB-3 (**3**)

position	δ_H, (mult, *J* in Hz)	δ_C, type
1		173.7, C
2	4.08, m	51.81, CH
2-NH	7.92, s	
3	1.50, m	41.6, CH_2
4	1.72, m	24.3, CH
5	0.91, d (7.0)	22.2, CH_3
6	0.88, d (7.0)	23.0, CH_3
7		171.1, C
8	4.50, m	52.8, CH
8-NH	8.21, d (5.0)	
9	3.16, m	27.1, CH_2
	3.13, m	
10		111.6, C
11	7.84, s	128.2, CH
12		128.1, C
13	7.61, d (8.0)	118.6, CH
14	7.13, t (8.0)	119.6, CH
15	7.20, m	122.1, CH
16	7.52, d (8.0)	109.6, CH
17		135.5, C
18		171.6, C
19	4.13, m	53.2, CH
19-NH	8.02, s	
20	1.96, m	27.3, CH_2
	1.73, m	
21	2.29, m	30.9, CH_2
	2.23, m	
22		173.8, C
23		170.3, C
24	4.42, td (10.0; 4.0)	53.1, CH
24-NH	7.23, s	
25	2.53, m	36.8, CH_2
	2.40, d (10.0)	
26		128.0, C
27/27′	7.0, d (8.5)	130.1, CH
28/28′	6.59, d (8.5)	114.7, CH
29		155.6, C
30		172.5, C
31	3.40, m	55.8, CH
32	3.01; m	31.4, CH_2
	2.75, t (12.0)	
33		109.9, C
34	6.69, d (2.0)	124.6, CH
34-NH	10.45, s	
35		129.2, C
36	7.58, d (7.5)	117.0, CH
37	7.16, t (7.5)	118.3, CH
38	7.24, d (7.5)	116.3, CH
39		123.8, C
40		131.6, C

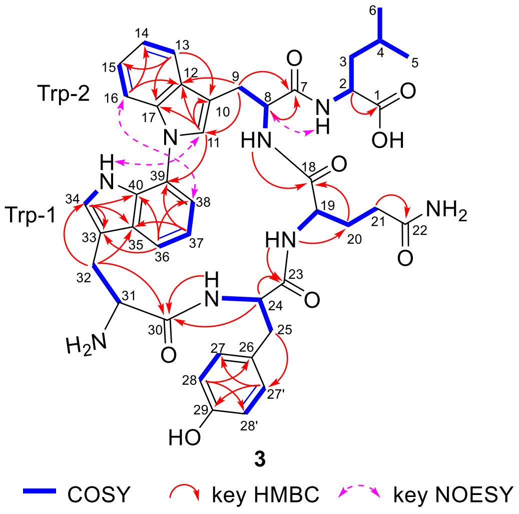

3

Table S5. NMR data of scabrirubin CB-4 (**4**)

position	δ_H, (mult, *J* in Hz)	δ_C, type
1		170.1, C
2	3.96, m	54.7, CH
2-NH		
3	2.96, m	35.3, CH_2
4		129.5, C
5	6.81, br s	132.6, CH[a]
6		129.1, C
7		152.1, C
8	6.75, d (8.5)	115.1, CH
9	6.86, dd (8.5; 2)	128.1, CH
10		171.0, C
11	4.22, m	50.6, CH
11-NH	7.37, d (9.0)	
12	1.24, m	41.0, CH_2
13	1.41, m	23.8, CH
14	0.80, d (6.5)	22.9, CH_3
15	0.75, d (6.5)	21.7, CH_3
16		170.2, C
17	3.67, quint (6.5)	47.1, CH
17-NH	7.34, s	
18	0.29, d (6.5)	16.6, CH_3
19		172.7, C
20	3.55, t (5.0)	56.6, CH
21	3.12, dd (14.5; 5.0)	31.2, CH_2
	2.89, m	
22		110.5, C
23	6.96, d (2.0)	124.5, CH
23-NH	10.57, d (2.0)	
24		126.5, C
25	7.87, d (8.5)	120.3, CH
26	6.91, dd (8.5; 1.5)	119.9, CH
27		131.7, C
28	7.33, s	111.4, CH
29		136.6, C

[a]Assigned based on the ^1H–^{13}C HSQC cross peak.

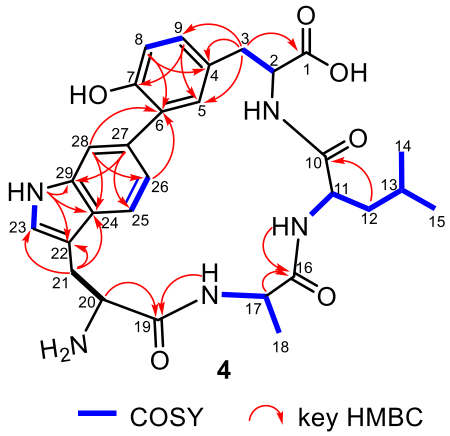

Table S6 Strains and plasmids used in this study

Strains/plasmids	Characteristic(s)	Sources
E.coli		
DH5a	Host strain for cloning	[2]
ET12567/pUZ8002	Donor strain for conjugation	[3]
Xanthomonas		
Xanthomonas vasicola strain NCPPB 989	Wild type strain containing *xavA*	DSMZ
Xanthomonas vasicola pv. vasculorum NCPPB 206	Wild type strain containing *xvvA*	BCCM
Xanthomonas vasicola pv. vasculorum NCPPB 895	Wild type strain containing *xvpA*	BCCM
Xanthomonas translucens pv. translucens DSM 18974	Wild type strain containing *xatA*	DSMZ
Streptomyces		
Streptomyces capoamus DSM 40494	Wild type strain containing *capA*	DSMZ
Streptomyces lucensis DSM 40317	Wild type strain containing *lucA*	DSMZ
Streptomyces noursei DSM 40635	Wild type strain containing *nouA*	DSMZ
Streptomyces kanamyceticus DSM 40500	Wild type strain containing *skaA*	DSMZ
Streptomyces katrae DSM 40550	Wild type strain containing *katA*	DSMZ
S. albus J1074	Heterologous host	[4]
S. albus J1074/*sca*	*S.albus* J1074 harboring *sca* BGC	[5]
S. albus J1074/*sva*	*S.albus* J1074 harboring *sva* BGC	[5]
S. albus J1074/*lauA+lauB1*	*S.albus* J1074 harboring *lauA* and *lauB1*	[5]
S. albus J1074/*svaA*	*S.albus* J1074 harboring *svaA*	This study
S. albus J1074/*scaA+lauB1*	*S.albus* J1074 harboring *scaA* and *lauB1*	This study
S. albus J1074/*scaA+svaB*	*S.albus* J1074 harboring *scaA* and *svaB*	This study
S. albus J1074/*svaA+lauB1*	*S.albus* J1074 harboring *svaA* and *lauB1*	This study
S. albus J1074/*svaA+scaB*	*S.albus* J1074 harboring *svaA* and *scaB*	This study
S. albus J1074/*lauA*	*S.albus* J1074 harboring *lauA*	This study
S. albus J1074/*lauA+svaB*	*S.albus* J1074 harboring *lauA* and *svaB*	This study
S. albus J1074/*lauA+scaB*	*S.albus* J1074 harboring *lauA* and *scaB*	This study
S. albus J1074/*nouA*	*S.albus* J1074 harboring *nouA*	This study
S. albus J1074/*nouA+scaB*	*S.albus* J1074 harboring *nouA* and *scaB*	This study
S. albus J1074/*katA*	*S.albus* J1074 harboring *katA*	This study
S. albus J1074/*katA+scaB*	*S.albus* J1074 harboring *katA* and *scaB*	This study
S. albus J1074/*jumA*	*S.albus* J1074 harboring *jumA*	This study
S. albus J1074/*jumA+scaB*	*S.albus* J1074 harboring *jumA* and *scaB*	This study
S. albus J1074/*lucA*	*S.albus* J1074 harboring *lucA*	This study
S. albus J1074/*lucA+scaB*	*S.albus* J1074 harboring *lucA* and *scaB*	This study
S. albus J1074/*capA*	*S.albus* J1074 harboring *capA*	This study
S. albus J1074/*capA+scaB*	*S.albus* J1074 harboring *capA* and *scaB*	This study
S. albus J1074/*skaA*	*S.albus* J1074 harboring *skaA*	This study
S. albus J1074/*skaA+scaB*	*S.albus* J1074 harboring *skaA* and *scaB*	This study
S. albus J1074/*xatA*	*S.albus* J1074 harboring *xatA*	This study

S. albus J1074/*xatA+scaB*	*S.albus* J1074 harboring *xatA* and *scaB*	This study
S. albus J1074/*xaaA*	*S.albus* J1074 harboring *xaaA*	This study
S. albus J1074/xaaA+scaB	*S.albus* J1074 harboring *xaaA* and *scaB*	This study
S. albus J1074/*xamA1*	*S.albus* J1074 harboring *xamA1*	This study
S. albus J1074/*xamA1+scaB*	*S.albus* J1074 harboring *xamA1* and *scaB*	This study
S. albus J1074/*xamA2*	*S.albus* J1074 harboring *xamA2*	This study
S. albus J1074/*xamA2+scaB*	*S.albus* J1074 harboring *xamA2* and *scaB*	This study
S. albus J1074/*xvpA*	*S.albus* J1074 harboring *xvpA*	This study
S. albus J1074/*xvpA+scaB*	*S.albus* J1074 harboring *xvpA* and *scaB*	This study
S. albus J1074/*xavA*	*S.albus* J1074 harboring *xavA*	This study
S. albus J1074/*xavA+scaB*	*S.albus* J1074 harboring *xavA* and *scaB*	This study
S. albus J1074/*xvvA*	*S.albus* J1074 harboring *xvvA*	This study
S. albus J1074/*xvvA+scaB*	*S.albus* J1074 harboring *xvvA* and *scaB*	This study
S. albus J1074/sca*A*$_{16-28}$+*scaB*	*S.albus* J1074 harboring sca*A*$_{16-28}$ and *scaB*	This study
S. albus J1074/ sca*A*$_{20-28}$+*scaB*	*S.albus* J1074 harboring sca*A*$_{20-28}$ and *scaB*	This study
S. albus J1074/*scaA-F24X+scaB*<br>*(X = A/E/H/L/N/P/Q/R/T)*	*S.albus* J1074 harboring mutated *scaA-F24X* and *scaB*	This study
S. albus J1074/*scaA-I25X+scaB*<br>*(X = A/E/H/L/N/P/Q/R/T/Y)*	*S.albus* J1074 harboring mutated *scaA-I25X* and *scaB*	This study
S. albus J1074/*scaA-W26X+scaB*<br>*(X = T/S/H/Y)*	*S.albus* J1074 harboring mutated *scaA-W26X* and *scaB*	This study
S. albus J1074/*scaA-W23Y+scaB*	*S.albus* J1074 harboring mutated *scaA-W23Y* and *scaB*	This study
S. albus J1074/*xamA2-W22X+scaB*<br>*(X = H/Y)*	*S.albus* J1074 harboring mutated *xamA2-W22X* and *scaB*	This study
S. albus J1074/*xamA2-Y25X+scaB*<br>*(X = H/W)*	*S.albus* J1074 harboring mutated *xamA2-Y25X* and *scaB*	This study
S. albus J1074/*xaaA-W23X+scaB*<br>*(X = H/Y)*	*S.albus* J1074 harboring mutated *xaaA-W23X* and *scaB*	This study
S. albus J1074/*xaaA-Y26X+scaB*<br>*(X = H/W)*	*S.albus* J1074 harboring mutated *xaaA-Y26X* and *scaB*	This study
**plasmids**		
pUWL201	Apr^r, *ermE**p, replicative expression vector in *Streptomyces*	[6]
pUWL201-OriT-*sca*	pUWL201 derivative containing *sca* BGC	[5]
pUWL201-OriT-*sva*	pUWL201 derivative containing *sva* BGC	[5]
pUWL201-OriT-*lauA+lauB1*	pUWL201 derivative containing *lauA* and *lauB1*	[5]
pUWL201-OriT-*jum*	pUWL201 derivative containing *jum* BGC	[5]
pUWL201-OriT-*svaA*	pUWL201 derivative containing *svaA*	This study
pUWL201-OriT-*scaA+lauB1*	pUWL201 derivative containing *scaA* and *lauB1*	This study
pUWL201-OriT-*scaA+svaB*	pUWL201 derivative containing *scaA* and *svaB*	This study
pUWL201-OriT-*svaA+lauB1*	pUWL201 derivative containing *svaA* and *lauB1*	This study
pUWL201-OriT-*svaA+scaB*	pUWL201 derivative containing *svaA* and *scaB*	This study
pUWL201-OriT-*lauA+scaB*	pUWL201 derivative containing *lauA* and *scaB*	This study
pUWL201-OriT-*lauA*	pUWL201 derivative containing *lauA*	This study

pUWL201-OriT-*lauA+svaB*	pUWL201 derivative containing *lauA* and *svaB*	This study
pUWL201-OriT-*nouA*	pUWL201 derivative containing *nouA*	This study
pUWL201-OriT-*nouA+scaB*	pUWL201 derivative containing *nouA* and *scaB*	This study
pUWL201-OriT-*katA*	pUWL201 derivative containing *katA*	This study
pUWL201-OriT-*katA+scaB*	pUWL201 derivative containing *katA* and *scaB*	This study
pUWL201-OriT-*jumA*	pUWL201 derivative containing *jumA*	This study
pUWL201-OriT-*jumA+scaB*	pUWL201 derivative containing *jumA* and *scaB*	This study
pUWL201-OriT-*lucA*	pUWL201 derivative containing *lucA*	This study
pUWL201-OriT-*lucA+scaB*	pUWL201 derivative containing *lucA* and *scaB*	This study
pUWL201-OriT-*capA*	pUWL201 derivative containing *capA*	This study
pUWL201-OriT-*capA+scaB*	pUWL201 derivative containing *capA* and *scaB*	This study
pUWL201-OriT-*skaA*	pUWL201 derivative containing *skaA*	This study
pUWL201-OriT-*skaA+scaB*	pUWL201 derivative containing *skaA* and *scaB*	This study
pUWL201-OriT-*xatA*	pUWL201 derivative containing *xatA*	This study
pUWL201-OriT-*xatA+scaB*	pUWL201 derivative containing *xatA* and *scaB*	This study
pUWL201-OriT-*xaaA*	pUWL201 derivative containing *xaaA*	This study
pUWL201-OriT-*xaaA+scaB*	pUWL201 derivative containing *xaaA* and *scaB*	This study
pUWL201-OriT-*xamA1*	pUWL201 derivative containing *xamA1*	This study
pUWL201-OriT-*xamA1+scaB*	pUWL201 derivative containing *xamA1* and *scaB*	This study
pUWL201-OriT-*xamA2*	pUWL201 derivative containing *xamA2*	This study
pUWL201-OriT-*xamA2+scaB*	pUWL201 derivative containing *xamA2* and *scaB*	This study
pUWL201-OriT-*xvpA*	pUWL201 derivative containing *xvpA*	This study
pUWL201-OriT-*xvpA+scaB*	pUWL201 derivative containing *xvpA* and *scaB*	This study
pUWL201-OriT-*xavA*	pUWL201 derivative containing *xavA*	This study
pUWL201-OriT-*xavA+scaB*	pUWL201 derivative containing *xavA* and *scaB*	This study
pUWL201-OriT-*xvvA*	pUWL201 derivative containing *xvvA*	This study
pUWL201-OriT-*xvvA+scaB*	pUWL201 derivative containing *xvvA* and *scaB*	This study
pUWL201-OriT-$scaA_{16\text{-}28}$+*scaB*	pUWL201 derivative containing $scaA_{16\text{-}28}$ and *scaB*	This study
pUWL201-OriT-$scaA_{20\text{-}28}$+*scaB*	pUWL201 derivative containing $scaA_{20\text{-}28}$ and *scaB*	This study
pUWL201-OriT-*scaA-F24A+scaB*	pUWL201 derivative containing *scaA-F24A* and *scaB*	This study
pUWL201-OriT-*scaA-F24E+scaB*	pUWL201 derivative containing *scaA-F24E* and *scaB*	This study
pUWL201-OriT-*scaA-F24H+scaB*	pUWL201 derivative containing *scaA-F24H* and *scaB*	This study
pUWL201-OriT-*scaA-F24L+scaB*	pUWL201 derivative containing *scaA-F24L* and *scaB*	This study
pUWL201-OriT-*scaA-F24N+scaB*	pUWL201 derivative containing *scaA-F24N* and *scaB*	This study
pUWL201-OriT-*scaA-F24P+scaB*	pUWL201 derivative containing *scaA-F24P* and *scaB*	This study
pUWL201-OriT-*scaA-F24Q+scaB*	pUWL201 derivative containing *scaA-F24Q* and *scaB*	This study
pUWL201-OriT-*scaA-F24R+scaB*	pUWL201 derivative containing *scaA-F24R* and *scaB*	This study
pUWL201-OriT-*scaA-F24T+scaB*	pUWL201 derivative containing *scaA-F24T* and *scaB*	This study
pUWL201-OriT-*scaA-I25A+scaB*	pUWL201 derivative containing *scaA-I25A* and *scaB*	This study
pUWL201-OriT-*scaA-I25E+scaB*	pUWL201 derivative containing *scaA-I25E* and *scaB*	This study
pUWL201-OriT-*scaA-I25H+scaB*	pUWL201 derivative containing *scaA-I25H* and *scaB*	This study
pUWL201-OriT-*scaA-I25L+scaB*	pUWL201 derivative containing *scaA-I25L* and *scaB*	This study
pUWL201-OriT-*scaA-I25N+scaB*	pUWL201 derivative containing *scaA-I25N* and *scaB*	This study
pUWL201-OriT-*scaA-I25P+scaB*	pUWL201 derivative containing *scaA-I25P* and *scaB*	This study

pUWL201-OriT-*scaA-I25Q+scaB*	pUWL201 derivative containing *scaA-I25Q* and *scaB*	This study
pUWL201-OriT-*scaA-I25R+scaB*	pUWL201 derivative containing *scaA-I25R* and *scaB*	This study
pUWL201-OriT-*scaA-I25T+scaB*	pUWL201 derivative containing *scaA-I25T* and *scaB*	This study
pUWL201-OriT-*scaA-I25Y+scaB*	pUWL201 derivative containing *scaA-I25Y* and *scaB*	This study
pUWL201-OriT-*xamA2-Y25W+scaB*	pUWL201 derivative containing *xamA2-Y25W* and *scaB*	This study
pUWL201-OriT-xamA2-Y25H+scaB	pUWL201 derivative containing xamA2-Y25H and scaB	This study
pUWL201-OriT-xamA2-W22H+scaB	pUWL201 derivative containing xamA2-W22H and scaB	This study
pUWL201-OriT-xamA2-W22Y+scaB	pUWL201 derivative containing xamA2-W22Y and scaB	This study
pUWL201-OriT-xaaA-Y26W+scaB	pUWL201 derivative containing xaaA-Y26W and scaB	This study
pUWL201-OriT-xaaA-Y26H+scaB	pUWL201 derivative containing xaaA-Y26H and scaB	This study
pUWL201-OriT-xaaA-W23Y+scaB	pUWL201 derivative containing xaaA-W23Y and scaB	This study
pUWL201-OriT-xaaA-W23H+scaB	pUWL201 derivative containing xaaA-W23H and scaB	This study
pUWL201-OriT-scaA-W26T+scaB	pUWL201 derivative containing scaA-W26T and scaB	This study
pUWL201-OriT-scaA-W26S+scaB	pUWL201 derivative containing scaA-W26S and scaB	This study
pUWL201-OriT-scaA-W26H+scaB	pUWL201 derivative containing scaA-W26H and scaB	This study
pUWL201-OriT-scaA-W26Y+scaB	pUWL201 derivative containing scaA-W26Y and scaB	This study
pUWL201-OriT-scaA-W23Y+scaB	pUWL201 derivative containing scaA-W23Y and scaB	This study

Table S7. Primers used in this study

Primers	Fragment	Sequences (5' to 3')
**Combinatorial biosynthesis of P450 with different precursors**		
**Construction of pUWL201-OriT/lauA+svaB**		
LA-F	*lauA* containing	cgtctagaacaggaggccccatATGAAGCTTCTCTTCGCC
SvaB-LA-R		cattggcatgggtgacctcctCGAGCGGACAGACATCGGCG
LA-svaB-F	*svaB* and pUWL backbone	aggaggtcacccatgccaatg
pUWL-OriT-R		atggggcctcctgttctag
**Construction of pUWL201-OriT/scaA+svaB**		
scaA-F	*svaA* containing	GTCTAGAACAGGAGGCCCCATGTGATCAAGATCGTCAACTC
sva-scaA-R		tggcatgggtgacctcctCGGTGCTCTCCACATGGCGC
Sca-svaB-F	*svaB* and pUWL backbone	GCCATGTGGAGAGCACCGaggaggtcacccatgccaatg
pUWL-OriT-R		atggggcctcctgttctag
**Construction of pUWL201-OriT/scaA+lauB1**		
scaA-F	*scaA* containing	GTCTAGAACAGGAGGCCCCATGTGATCAAGATCGTCAACTC
sca-LB1-R		GTGGAACACCGTGGCGCGCATCGGTGCTCTCCACATGGCGC
LauB1-F	*lauB1* and pUWL backbone	ATGCGCGCCACGGTGTTCCAC
pUWL-OriT-R		atggggcctcctgttctag
**Construction of pUWL201-OriT/svaA+lauB1**		
svaA -F	*svaA* containing	gtctagaacaggaggccccatatggaggaatttatgaagc
sva-LB1-R		TGGAACACCGTGGCGCGCATgggtgacctcctactccgttg
LauB1-F	*lauB1* and pUWL backbone	ATGCGCGCCACGGTGTTCCAC
pUWL-OriT-R		atggggcctcctgttctag
**Construction of pUWL201-OriT/svaA+scaB**		
svaA -F	*svaA* containing	gtctagaacaggaggccccatatggaggaatttatgaagc
Sva-scaB-R		CGTATCGGAACGACTCATgggtgacctcctactccg
scab-svaA-F	*scaB* and pUWL backbone	cggagtaggaggtcacccATGAGTCGTTCCGATACGG
pUWL-OriT-R		atggggcctcctgttctag
**Construction of pUWL201-OriT/lauA+scaB**		
LA-F	*lauA* containing	cgtctagaacaggaggccccatATGAAGCTTCTCTTCGCC
lauA-scaB-R		GTTCTCAGGATGTGCGGACTAGTACCAGATGTACCAG
scaB-VF	*scaB* and pUWL backbone	TCCGCACATCCTGAGAAC
scaB-VR		ATGGGGCCTCCTGTTCTAG
**Precursors coexpressed with ScaB**		
scaB-VF	*scaB* and pUWL backbone	TCCGCACATCCTGAGAAC
scaB-VR		ATGGGGCCTCCTGTTCTAG
**Construction of pUWL201-OriT/stlA+scaB**		
stlA-F1	*stlA*	GTCTAGAACAGGAGGCCCCATATGAAGCTCCTCTTCGCC
stlA-R1		CGGTTCTCAGGATGTGCGGATCAGTACCAGAGGTACCAG
**Construction of pUWL201-OriT/scaA+scaB**		
scaA-F	*scaA*	CTAGAACAGGAGGCCCCATATGAAGCTCCTTTTCGCCC
scaA-R		CGGTTCTCAGGATGTGCGGATCAGTACCAGAGGTACCAG
**Construction of pUWL201-OriT/xamA1+scaB**		
xamA1-F	*xamA1*	CTAGAACAGGAGGCCCCATATGAAGCTAATGCTTACC
xamA1-scaB-R		GGTTCTCAGGATGTGCGGATCAGTACCAGTGCGGCCAAG

**Construction of pUWL201-OriT/xamA2+scaB**		
xamA2-scaB-F	*xamA2*	CTAGAACAGGAGGCCCCATATGAAGCTGCTTTTTTCCATC
xamA2-scaB-R		GTTCTCAGGATGTGCGGACTAGTAGGAGATGTATCCCTC
**Construction of pUWL201-OriT/skaA+scaB**		
skaA-F	*skaA*	gtctagaacaggaggccccatATGAAGATCGTTCGCTCG
skaA-R		GGTTCTCAGGATGTGCGGACTAGTACCAGTGGTACCAG
**Construction of pUWL201-OriT/jumA+scaB**		
JumA-F	*jumA*	GTCTAGAACAGGAGGCCCCATATGAAGGTTCTCTTTGCC
JumA-R		CGGTTCTCAGGATGTGCGGATCACCACCAGATGTACCAG
**Construction of pUWL201-OriT/nouA+scaB**		
nouA-scaB-F	*nouA*	GTCTAGAACAGGAGGCCCCATATGAAGTTCGTTCGTCTCG
nouA-scaB-R		CGGTTCTCAGGATGTGCGGATCAGATCCAGCCGTACCAG
**Construction of pUWL201-OriT/xvpA+scaB**		
xvpA-scaB-F	*xvpA*	GTCTAGAACAGGAGGCCCCATATGAAGATACTTTTTTCG
xvpA-scaB-R		CGGTTCTCAGGATGTGCGGACTAAAAATATTCGGTCCAAG
**Construction of pUWL201-OriT/xvvA+scaB**		
xvvA-scaB-F	*xvvA*	GTCTAGAACAGGAGGCCCCATATGAAGATACTTTTTTCG
xvvA-scaB-R		CCGGTTCTCAGGATGTGCGGATTAAAAATATTCGGGCCAAG
**Construction of pUWL201-OriT/xatA +scaB**		
xatA-scaB-F	*xatA*	GTCTAGAACAGGAGGCCCCATATGAACGTGCTTTTTTCG
xatA-scaB-R		GGTTCTCAGGATGTGCGGATCAATACCAGTGTAACCAG
**Construction of pUWL201-OriT/xaaA+scaB**		
xaaA-F	*xaaA*	gtctagaacaggaggccccatATGAAGCTAATGCTTACTATC
xaaA-R		CGGTTCTCAGGATGTGCGGATCAGTACGAGATGTATAAAG
**Construction of pUWL201-OriT/xavA+scaB**		
xavA-F	*xavA*	GTCTAGAACAGGAGGCCCCATATGAAGATACTTTTTTCGATC
xavA-scaB-R		GGTTCTCAGGATGTGCGGATCACATATATAAACCCCAAG
**Construction of pUWL201-OriT/katA+scaB**		
KA-scaB-F	*scaB* and pUWL backbone	GCCCATCGGAGCCTCCGTCGCGAGACCTGCGCCATGTG
pUWL-OriT-R		ATGGGGCCTCCTGTTCTAG
kauA-F	*katA* containing	gtctagaacaggaggccccatATGAAGATTCTTTTCGCCATC
KA-scaB-R		GCAGGTCTCGCGACGGAGGCTCCGATGGGC
**Construction of pUWL201-OriT/xavA/xvvA/xvpA/xatA/jumA/katA/capA/lucA/xaaA/xamA1/xamA2/nouA/lauA/skaA**		
pUWL-OriT-F		aagcttgatatcgaattcc
precursor-puwl-R		GGAATTCGATATCAAGCTTCGGTGCTCTCCACATGGCGCAG
**Construction of pUWL201-OriT/svaA**		
pUWL-OriT-F		aagcttgatatcgaattcc
SvaA-puwl-R		caggaattcgatatcaagctttccgttgctgggctgcccg
**Construction of pUWL201-scaA$_{16-28}$+scaB**		
scaA16-28-F		GTCTAGAACAGGAGGCCCCATGTGAAGTCCCTGAAGTCC
pUWL-oriT-R		atggggcctcctgttctag
**Construction of pUWL201-scaA$_{20-28}$+scaB**		
scaA20-28-F		CTAGAACAGGAGGCCCCATGTGTCCGCCACCTGGTTCATC
pUWL-oriT-R		atggggcctcctgttctag
**For mutation of ScaA-F24X**		

ScaA-F24A-F	ScaA-F24A	CTGAAGTCCGCCACCTGGgcCATCTGGTACAGCTGATC
ScaA-F24A-R		gcCCAGGTGGCGGACTTCAGGGACTTCTCGGTCCTG
ScaA-F24E-F	ScaA-F24E	CTGAAGTCCGCCACCTGGgagATCTGGTACAGCTGATC
ScaA-F24E-R		ctcCCAGGTGGCGGACTTCAGGGACTTCTCGGTCCTG
ScaA-F24H-F	ScaA-F24H	CTGAAGTCCGCCACCTGGcaCATCTGGTACAGCTGATC
ScaA-F24H-R		tgCCAGGTGGCGGACTTCAGGGACTTCTCGGTCCTG
ScaA-F24L-F	ScaA-F24L	CTGAAGTCCGCCACCTGGcTCATCTGGTACAGCTG
ScaA-F24L-R		gCCAGGTGGCGGACTTCAGGGACTTCTCGGTCCTG
ScaA-F24N-F	ScaA-F24N	CTGAAGTCCGCCACCTGGaaCATCTGGTACAGCTGATC
ScaA-F24N-R		ttCCAGGTGGCGGACTTCAGGGACTTCTCGGTCCTG
ScaA-F24P-F	ScaA-F24P	CTGAAGTCCGCCACCTGGccaATCTGGTACAGCTGATC
ScaA-F24P-R		tggCCAGGTGGCGGACTTCAGGGACTTCTCGGTCCTG
ScaA-F24Q-F	ScaA-F24Q	CTGAAGTCCGCCACCTGGcagATCTGGTACAGCTGATC
ScaA-F24Q-R		ctgCCAGGTGGCGGACTTCAGGGACTTCTCGGTCCTG
ScaA-F24R-F	ScaA-F24R	CTGAAGTCCGCCACCTGGcgCATCTGGTACAGCTG
ScaA-F24R-R		cgCCAGGTGGCGGACTTCAGGGACTTCTCGGTCCTG
ScaA-F24T-F	ScaA-F24T	CTGAAGTCCGCCACCTGGacCATCTGGTACAGCTGATC
ScaA-F24T-R		gtCCAGGTGGCGGACTTCAGGGACTTCTCGGTCCTG
**For mutation of ScaA-I25X**		
ScaA-I25A-F	ScaA-I25A	GAAGTCCGCCACCTGGTTCgcCTGGTACAGCTGATCC
ScaA-I25A-R		gcGAACCAGGTGGCGGACTTCAGGGACTTCTCGGTCC
ScaA-I25E-F	ScaA-I25E	GAAGTCCGCCACCTGGTTCgagTGGTACAGCTGATCCG
ScaA-I25E-R		ctcGAACCAGGTGGCGGACTTCAGGGACTTCTCGGTCC
ScaA-I25H-F	ScaA-I25H	GAAGTCCGCCACCTGGTTCcaCTGGTACAGCTGATCC
ScaA-I25H-R		tgGAACCAGGTGGCGGACTTCAGGGACTTCTCGGTCC
ScaA-I25L-F	ScaA-I25L	GAAGTCCGCCACCTGGTTCcTCTGGTACAGCTGATC
ScaA-I25L-R		gGAACCAGGTGGCGGACTTCAGGGACTTCTCGGTCC
ScaA-I25N-F	ScaA-I25N	GAAGTCCGCCACCTGGTTCAaCTGGTACAGCTGATC
ScaA-I25N-R		tTGAACCAGGTGGCGGACTTCAGGGACTTCTCGGTC
ScaA-I25P-F	ScaA-I25P	GAAGTCCGCCACCTGGTTCccCTGGTACAGCTGATCC
ScaA-I25P-R		ggGAACCAGGTGGCGGACTTCAGGGACTTCTCGGTC
ScaA-I25Q-F	ScaA-I25Q	GAAGTCCGCCACCTGGTTCcagTGGTACAGCTGATCCG
ScaA-I25Q-R		ctgGAACCAGGTGGCGGACTTCAGGGACTTCTCGGTCC
ScaA-I25R-F	ScaA-I25R	GAAGTCCGCCACCTGGTTCAgaTGGTACAGCTGATCCG
ScaA-I25R-R		tcTGAACCAGGTGGCGGACTTCAGGGACTTCTCGGTC
ScaA-I25T-F	ScaA-I25T	GAAGTCCGCCACCTGGTTCAcCTGGTACAGCTGATC
ScaA-I25T-R		gTGAACCAGGTGGCGGACTTCAGGGACTTCTCGGTCC
ScaA-I25Y-F	ScaA-I25Y	GAAGTCCGCCACCTGGTTCtaCTGGTACAGCTGATCC
ScaA-I25Y-R		taGAACCAGGTGGCGGACTTCAGGGACTTCTCGGTC
**For mutation of XamA2-Y25W**		
XamA2-Y25W-F	GTCCTCCGCTTGGGAGGGATggATCTCCTACTAGTCC	
XamA2-Y25W-R	ccATCCCTCCCAAGCGGAGGACTTCAGGCTCTTC	
**For mutation of XamA2-Y25H**		
XamA2-Y25H-F	GTCCTCCGCTTGGGAGGGAcACATCTCCTACTAGTC	
XamA2-Y25H-R	gTCCCTCCCAAGCGGAGGACTTCAGGCTCTTCTTGG	
**For mutation of XamA2-W22H**		

XamA2-W22H-F	GAGCCTGAAGTCCTCCGCTcacGAGGGATACATCTCC
XamA2-W22H-R	gtgAGCGGAGGACTTCAGGCTCTTCTTGGAGGAGAGTAG
**For mutation of XamA2-W22Y**	
XamA2-W22Y-F	GCCTGAAGTCCTCCGCTTacGAGGGATACATCTCC
XamA2-W22Y-R	gtAAGCGGAGGACTTCAGGCTCTTCTTGGAGGAG
**For mutation of XaaA-Y26H**	
XaaA-Y26H-F	GTCCTCTGCTTGGGCTTTAcACATCTCGTACTGATC
XaaA-Y26H-R	gTAAAGCCCAAGCAGAGGACTTCAAGCTCTTCTTG
**For mutation of XaaA-Y26W**	
XaaA-Y26W-F	GTCCTCTGCTTGGGCTTTATggATCTCGTACTGATCCG
XaaA-Y26W-R	ccATAAAGCCCAAGCAGAGGACTTCAAGCTCTTCTTG
**For mutation of XaaA-W23H**	
XaaA-W23H-F	GAGCTTGAAGTCCTCTGCTcacGCTTTATACATCTCG
XaaA-W23H-R	gtgAGCAGAGGACTTCAAGCTCTTCTTGTTGGAAGAAAG
**For mutation of XaaA-W23Y**	
XaaA-W23Y-F	GAGCTTGAAGTCCTCTGCTTacGCTTTATACATCTCG
XaaA-W23Y-R	gtAAGCAGAGGACTTCAAGCTCTTCTTGTTGGAAG
**For mutation of ScaA-W26T**	
ScaA-W26T-F	GTCCGCCACCTGGTTCATCacGTACAGCTGATCCGCAC
ScaA-W26T-R	gtGATGAACCAGGTGGCGGACTTCAGGGACTTCTCGG
**For mutation of ScaA-W26S**	
ScaA-W26S-F	GTCCGCCACCTGGTTCATCTcGTACAGCTGATCCGCAC
ScaA-W26S-R	gAGATGAACCAGGTGGCGGACTTCAGGGACTTCTCGG
**For mutation of ScaA-W26H**	
ScaA-W26H-F	GTCCCTGAAGTCCGCCACCcacTTCATCTGGTACAGCTG
ScaA-W26H-R	gtgGGTGGCGGACTTCAGGGACTTCTCGGTCCTGAACC
**For mutation of ScaA-W26Y**	
ScaA-W26Y-F	CGCCACCTGGTTCATCTACTACAGCTGATCCG
ScaA-W26Y-R	GTAGATGAACCAGGTGGCGGACTTCAGGGAC
**For mutation of ScaA-W23Y**	
ScaA-W23Y-F	GTCCCTGAAGTCCGCCACCTACTTCATCTGGTAC
ScaA-W23Y-R	GTAGGTGGCGGACTTCAGGGACTTCTCGGTCCTG

Materials and Methods

General materials

All chemicals, reagents and solvents were purchased from Sigma-Aldrich or Carl Roth (Germany), unless stated otherwise. Primer synthesis and plasmids sequencing were performed by Microsynth AG (Germany). Gene synthesis was performed by Integrated DNA Technologies (USA). All restriction enzymes, Q5 High Fidelity DNA polymerase, HiFi DNA Assembly master mix and deoxynucleotides (dNTPs) were purchased from New England Biolabs (UK). Monarch® DNA gel extraction kit, Monarch® Plasmid miniprep kit, Monarch® Genomic DNA Purification kit and Monarch® PCR Clean-up kit were purchased from New England Biolabs (UK).

Strains and culture conditions

Bacterial strains and plasmids used and constructed in this study are summarized in Table S6. *Escherichia coli* strains were cultured in Luria-Bertani (LB) medium or on LB agar (10 g/L tryptone, 5 g/L yeast extract, 10 g/L NaCl, 20 g/L agar) overnight at 37 °C. *Streptomyces albus* J1074 and its derivatives were cultured in MS medium (20 g/L mannitol, 20 g/L soybean flour, 20 g/L agar) at 30 °C for growth, sporulation and conjugation (MS medium with 20 mM $MgCl_2$). *Xanthomonas vasicola* strain NCPPB 989 (medium 9), *Xanthomonas vasicola* pv. *vasculorum* NCPPB 206 (medium 9), *Xanthomonas vasicola* pv. *vasculorum* NCPPB 895 (medium 9), *Xanthomonas translucens* DSM 18974 (medium 1) and *Streptomyces kanamyceticus* strains ATCC 12853 (ISP2) were cultured at 30 °C in the corresponding medium for growth. The small-scale or large-scale fermentation of recombinant strains was performed in Tryptic soy broth medium (TSB) at 30 °C for 6 to 7 days. For the cultivation of strains harboring plasmids, the medium was supplementary with the corresponding antibiotics with the final concentration: 50 µg/mL kanamycin, 50 µg/mL chloramphenicol, 50 µg/mL apramycin.

DNA isolation, manipulation and sequencing

The DNA sequence of *xaaA*, *xamA1* and *xamA2* was synthesized by GeneArt (ThermoFisher Scientific). The corresponding sequences are listed in the materials and methods section. DNA isolation and manipulation in *Escherichia coli*, *Xanthomonas* and *Streptomyces* were performed using Monarch® Genomic DNA Purification kit according to manufacturer's instructions unless stated otherwise. Primers used in this study are listed in Table S7. PCR reactions using Q5 High Fidelity DNA polymerase were prepared according to the manufacturer's protocol. PCR reactions were performed using the following conditions: initial denaturation (95 °C, 10 min), 30 cycles of denaturation (95 °C, 30 s), annealing (55 °C –68 °C, depending on the melting temperature of each primer, 30 s) and elongation (72 °C, time depending on the length of PCR product, 30 s/1 kb), and final extension (72 °C, 10 min). DNA fragments obtained by PCR were purified by agarose gel electrophoresis and isolated using the Monarch gel extraction kit according to the manufacturer's instructions. The amplified plasmid backbone or engineered plasmids were purified using a PCR Clean-up kit followed by *Dpn*I digestion according to the manufacturer's instructions.

General bioinformatics analysis

The amino acid identities were calculated with EMBOSS Needle.[7] A Maximum-likelihood phylogenetic tree of 288 non-redundant putative atropopeptide-modifying P450s identified by AtropoFinder,[5] with the $P450_{Blt}$ from *Micromonospora* sp. MW-13 (PDB: 8U2M)[1] as an outgroup was constructed. To do so, the amino acid sequences were retrived and aligned using MUSCLE 5.1 with default parameters. The alignment was trimmed using CIAlign[8] (CIAlign –infilep450_sequences_with_biarylitide.fasta --remove_insertions --visualise --interpret --crop_ends). The tree was created using IQ-TREE multicore version 2.0.7[9] (iqtree2 -s CIAlign_cleaned.fasta -m MFP -B 1000 -alrt 1000 -nt AUTO) and visualized using iTOL.[10] The sequence logos of the selected atropopeptide precursors were created with WebLog[11] using sequence alignments of

selected atropopeptide precursors generated by ClustalW (https://www.genome.jp/tools-bin/clustalw) with default settings. The P450-precursor complex modeling was performed using AlphaFold 2 in multimer mode with standard parameters.[12] The structures were visualized in pymol (http://www.pymol.org/). The substrate-binding pocket and its volume was predicted using POCASA[13].

Construction of recombinant plasmids

A) Plasmids used for combinatorial co-expression of three atropopeptide BGCs
The fragments containing the precursor encoding genes (*scaA*, *svaA*, *lauA*) and the backbones containing cytochrome P450 encoding genes (*scaB*, *svaB*, *lauB1*) were amplified by the primer pairs listed in Table S7 using the previously constructed plasmids pUWL201-OriT-*sca*, pUWL201-OriT-*svaR*, pUWL201-OriT-*lauA*+*lauB1* (Table S6)[5] as templates. After amplification, these fragments were purified, digested with *Dpn*I, and assembled in different combinations with HiFi assembly. The assembled product was subsequently transformed into *E. coli* DH5a. Recombinant plasmids were confirmed by sequencing.

B) Plasmids used for combinatorial co-expression of variable atropopeptide precursors with *scaB*
Different atropopeptide precursor encoding genes were amplified with the corresponding primer pairs (Table S7) using the genomic DNA or synthetic DNA as template. These fragments were then assembled with the backbone fragment containing the *scaB* gene, which was amplified with primer pairs ScaB-VF/VR (Table S7) using pUWL201-OriT-sca (Table S6) as template and digested with *Dpn*I. The assembled product was subsequently transformed into *E. coli* DH5a. Recombinant plasmids were confirmed by sequencing.

C) Plasmids used for expression of truncated ScaA variants
The primers used for constructing truncated ScaA variants are listed in Table S7. Plasmid pUWL201-OriT-sca (Table S6) was reversely amplified with each primer pair. The amplified fragments were purified, digested by *Dpn*I, and cyclized using the HiFi assembly kit. The assembled product was subsequently transformed into *E. coli* DH5a. The recombinant plasmids were confirmed by sequencing.

D) Plasmids used for site-directed mutagenesis of precursor peptides
The primers used for site-directed mutagenesis are listed in Table S7. Plasmid pUWL201-OriT-sca, pUWL201-OriT-xamA2+scaB, pUWL201-OriT-xaaA+scaB were reversely amplified, digested by *Dpn*I and then directly transformed into *E. coli* DH5a. The recombinant plasmids were confirmed by sequencing.

Heterologous expression of recombinant BGCs

The recombinant plasmids were transformed into *E. coli* ET12567/pUZ8002 strains, respectively, which were subsequently introduced into *S. albus* J1074 strains by conjugation. Briefly, the *E. coli* ET12567/pUZ8002 containing the recombinant plasmid was cultured in 10 mL of LB medium with appropriate antibiotics at 37 °C, 180 rpm to an OD_{600} 0.7–1.0. The cells were harvested, washed twice with 10 mL of LB medium and resuspended in 200 µL of LB medium. The resuspended cells were then used as donor strains. *S. albus* J1074 was streaked on MS agar plates and incubated at 30 °C for 5–7 days for sporulation. The *S. ablus* J1074 spores from a quarter of a MS agar plate were harvested, resuspended in 400 µL TSB medium and heated at 50 °C for 10 min. The spore suspensions were then incubated at 30 °C for 0.5–1h to serve as the recipient strains. The donor strains and the recipient strains were mixed and diluted 1000-fold with LB medium. After dilution, 200 µL of the mixed strains were spread onto MS agar plates containing 20 mM $MgCl_2$. After incubation at 30 °C for 14–16 hours, each plate was overlayed with apramycin (50 µg/mL) and trimethoprim (100 µg/mL) solutions and further incubated for 3–4 days until exconjugants were visible. The exconjugants were individually picked and re-streaked on MS agar plates containing apramycin (50 µg/mL) and trimethoprim (100 µg/mL) and incubated at 30 ℃ for 3–4 days. Two to three positive clones were randomly selected for small-scale fermentation and subjected to metabolite analysis by LC-MS.

Small-scale fermentation and LC-MS analysis

For small-scale fermentations, the exconjugants streaked onto MS agar were collected and transferred into a 250 mL Erlenmeyer flask containing 50 mL of TSB medium with apramycin (50 µg/mL) and incubated at 30 °C, 180 rpm shaking for 6 days. The culture was supplemented with 5% (w/v) Diaion HP-20 resin at the fourth day and then cultured for another two days. The resin was harvested by centrifugation (3214 x g, 10 min) and extracted with 15 mL acetone under sonication for 15 min. 1 mL of the extracts was dried under reduced pressure and dissolved in 1 mL of methanol. After centrifugation (3214 x g, 10 min), 50 µL of the crude extract was mixed with 100 µL of methanol, which was then subjected to LC-MS analysis.

High-performance liquid chromatography-electrospray ionization –quadrupole time of flight-high resolution mass spectrometry (HPLC-ESI-QTOF-HRMS) measurements were conducted on an Ultimate 3000 LC system (Thermo Fisher) coupled to an Impact II QTOF mass spectrometer (Bruker) with an ACQUITY UPLC BEH C18 column (130 Å, 2.1 mm × 100 mm, 1.7 µm particle size, Waters) at a flow rate of 0.4 mL/min at 40 ℃ (5%- 95% acetonitrile/Milli-Q water supplemented with 0.1 % (v/v) formic acid, 16 min). Data were acquired in positive mode at a scan range between 100 to 1200 *m*/*z*. 5 µL of sample was injected for each analysis. The software DataAnalysis 4.3 (Bruker) and Metabolite Detect (Bruker) were used to analyze the data.

Large-scale fermentation and purification of compounds

For the large-scale fermentation of the recombinant strains, 10 L cultures were prepared. The recombinant strains were taken from –80 °C, streaked on MS agar plates with 50 µg/mL apramycin and 100 µg/mL trimethoprim, and incubated at 30 °C for 6 to 7 days for sporulation. 4 L cultures were prepared as follows: the spores collected from one MS agar plate was transferred into a 5 L Erlenmeyer flask containing 1 L of TSB medium with 50 µg/mL apramycin. Another 6 L cultures were prepared as follows: the spores collected from one MS agar plate was transferred into five 1 L Erlenmeyer flasks with 200 mL of TSB medium containing 50 µg/mL apramycin in each flask. These 10 L cultures were incubated at 30 °C, 180 rpm shaking for total 7 days, then supplemented with 5% (w/v) Diaion HP-20 resin at the fourth days and incubated for another three days. The resins from the 10 L cultures were harvested by filtration through a metal sieve (40 mesh). The harvested resins were washed with water and extracted with 5 L of acetone. The extract was concentrated under reduced pressure, and the obtained crude extract was subjected to normal phase silica gel column chromatography (230 to 400 mesh) and eluted with $CHCl_3$/CH_3OH (1:0, 4:1, 2:1, 0:1(3 times), v/v, 600 mL) to yield 6 fractions (Fr. 1 to Fr. 6).

Compounds **1** and **2** were purified from the crude extract of the recombinant strains *S.albus*/*lauA*+*scaB*. The target compounds were first observed in Fr. 3 and Fr. 4 after normal phase silica gel column chromatography. These two fractions were subjected to preparative HPLC using a cholester C18 column (20ID × 250 mm, COSMOSIL) to afford Fr.1.1 (t_R = 10.8 min, containing compound **1**) and Fr.1.2 (t_R = 13.5 min, containing compound **2**) with an isocratic elution of acetonitrile/water 30% (0–20 min) at a flow rate of 10 mL/min. Fr.1.1 was further subjected to the semi-preparative HPLC (XBridge® peptide BEH C18, 10 mm × 250 mm, 5 µm particle size, Waters) to afford compound **1** (6.7 mg, t_R = 14 min) with an isocratic elution of acetonitrile/water 26% (0–20 min) at a flow rate of 3 mL/min and the peptides of interest were identified by UV at 280 nm. Fr.1.2 was further subjected to the semi-preparative HPLC using a cholester column (10ID × 250 mm, COSMOSIL) to afford compound **2** (2.8 mg, t_R = 14 min) with an isocratic elution of acetonitrile/water 26% (0–20 min) at a flow rate of 3 mL/min.

Compounds **3** were purified from the crude extract of the recombinant strain *S.albus*/*svaA*+*scaB*. The target compounds were first observed in Fr. 4 after normal phase silica gel column chromatography. Fr. 4 was subjected to preparative HPLC using an Edipse XDB C18 column (21.2 × 250 mm, 7 µm particle size,

Aglilent) to afford Fr.1.1 (t_R = 20 min, containing compound **3**) with a gradient elution of acetonitrile/water 25%–45% (0–20 min) at a flow rate of 10 mL/min. Fr.1.1 was further subjected to the semi-preparative HPLC (XBridge® peptide BEH C18, 10 mm × 250 mm, 5 μm particle size, Waters) to afford compound **3** (2.5 mg, t_R = 8 min) with an isocratic elution of acetonitrile/water 31% (0–20 min) at a flow rate of 3 mL/min.

Compound **4** was purified from the crude extract of the recombinant strain *S.albus/xaaA+scaB*. The target compound was first observed in Fr. 4 and Fr. 5 after normal phase silica gel column chromatography. These two fractions were subjected to preparative HPLC using a Edipse XDB C18 column (9.4 × 250 mm, 5 μm particle size, Aglilent) to afford Fr.1.1 (t_R = 18.6 min, containing compound **4**) with a gradient elution of acetonitrile/water 10%–13% (0-20 min) at a flow rate of 10 mL/min. Fr.1.1 was further purified by semi-preparative HPLC (XBridge® peptide BEH C18, 10 mm × 250 mm, 5 μm particle size, Waters) to afford compound **4** (1.33 mg, t_R = 18 min) with a gradient elution of acetonitrile/water 10%–30% (0–20 min) at a flow rate of 3 mL/min.

Physical and spectroscopic data of isolated compounds

Compound **1**: yellow powder (6.7 mg); ^{1}H and ^{13}C NMR data, see Table S2; HRMS (ESI-QTOF) *m*/*z* [M + H]$^+$ calcd for $C_{37}H_{41}N_6O_6^+$ 665.3082, found 665.3083;

Compound **2**: yellow powder (2.8 mg); ^{1}H and ^{13}C NMR data, see Table S3; HRMS (ESI-QTOF) *m*/*z* [M + H]$^+$ calcd for $C_{46}H_{50}N_7O_8^+$ 828.3715, found 828.3718;

Compound **3**: yellow powder (2.5 mg); ^{1}H and ^{13}C NMR data, see Table S4; HRMS (ESI-QTOF) *m*/*z* [M + H]$^+$ calcd for $C_{42}H_{49}N_8O_8^+$ 793.3667, found 793.3663;

Compound **4**: yellow powder (1.33 mg); ^{1}H and ^{13}C NMR data, see Table S5; HRMS (ESI-QTOF) *m*/*z* [M + H]$^+$ calcd for $C_{29}H_{36}N_5O_6^+$ 550.2660, found 550.2659.

NMR analysis

^{1}H, ^{13}C NMR, HMBC, HSQC, ^{1}H–^{1}H COSY and NOESY spectra were acquired on Bruker AV500 and DRX600 spectrometers. Chemical shifts (*δ*) were acquired in ppm with reference to the residual solvents of DMSO-*d*$_5$ (*δ* 2.50 for ^{1}H NMR), DMSO-*d*$_6$ (*δ* 39.51 for ^{13}C NMR). Spectra were processed using TOPSPIN 4.3.0 (Bruker).

Structure elucidation

Compound **1** was isolated as a yellow powder. Its molecular formula was determined to be $C_{37}H_{40}N_6O_6$ based on a protonated ion at *m*/*z* 665.3083 (calcd for $C_{37}H_{41}N_6O_6^+$, 665.3082, Δ 0.15 ppm) in HR-ESI-QTOF-MS data (Figure S17). Analysis of the ^{1}H and ^{13}C NMR spectra (Figures S17 and S18), with the aid of the HSQC spectrum (Figure S20), revealed the presence of 35 carbons including four carbonyl carbons, nine nonprotonated sp^2 carbons,11 sp^2 methines, five sp^3 methines, four sp^3 methylenes, and two methyls. These signals and amide proton signals (δ_H 7.99, 7.69, 7.35) are characteristic for peptides (Table S2). The typical AA′XX′ spin system (δ_H 7.02, 6.60) and benzyl methylene signals (δ_H 2.57, 2.46) in the ^{1}H NMR spectrum indicated the presence of a Tyr residue, which is supported by the HMBC correlations of H-19/C-18, H-19/C-21, H$_2$-20/C-21, H$_2$-20/C-22, H-22/C-24, and H-23/C-24 (Figures S21). A continuous spin system in the aromatic region (δ_H 7.66, 7.11, 7.18, 7.50), a singlet signal for an aromatic proton (δ_H 7.79), and nonequivalent aryl methylene signals (δ_H 3.24, 3.13) suggest the presence of a Trp residue (Trp-2). Another constituted spin system includes an aliphatic methyl doublet and an aliphatic methyl triplet (δ_H 7.69, 4.11, 1.69, 1.62, 1.35, 0.93, 0.90), indicating the presence of an Ile residue. Furthermore, the 7-substituted Trp (Trp-1) was assigned based on the ^{1}H–^{1}H COSY correlations of NH-29/H-29, H-33/H-32 and H-32/H-31 along with HMBC correlations from H-26 to C-28, from H$_2$-27 to C-28 and C-30, and from H-29 to C-28, C-30, and C-35, from H-31 to C-30, and C-35, and from H-32 to C-34 (Figures S19 and S21). The amino acid

sequence of **1** was determined to be NH_2-Trp-Tyr-Ile-Trp-CO_2H based on the HMBC correlations from amide protons to carbonyl carbons (Figure S21). The HMBC correlation from H-5 of Trp-2 to C-34 of Trp-1 suggested the presence of a C–N bond between C-34 of Trp-1 and N-5 of Trp-2. This aryl C–N bond was further confirmed by the NOESY correlations of H-5/NH-29 and H-10/H-33 (Figure S22). The stereochemistry of each amino acid was deduced as L-configured based on the ribosomal biosynthetic origin and no epimerization enzymes involved in its biosynthesis.

Compound **2** was isolated as a yellow powder. Its molecular formula was determined to be $C_{46}H_{49}N_7O_8$ based on a protonated ion at *m/z* 828.3718 (calcd for $C_{46}H_{50}N_7O_8^+$, 828.3715, Δ 0.36 ppm) in HR-ESI-QTOF-MS data (Figure S23). The ^1H and ^{13}C NMR spectra of **2** (Figure S24 and S25) are akin to those of **1** except for the presence of signals for an additional Tyr residue (Tyr-2), indicating that **2** is a pentapeptide congener of **1**. The NOSEY correlations of H-9/NH-2 and HMBC correlation from an α-proton H-2 of Tyr-2 to a carboxylic acid C-1 of Tyr-2, Tyr-2 was assigned to be a C-terminal amino acid connected to Trp-2 via a peptide bond (Figure S29). Furthermore, the HMBC correlation of H-12/C-41 and the NOESY correlations of H-12/NH-36 and H-17/H-40 confirmed the presence of a C–N bond between C-41 of Trp-1 and N-12 of Trp-2 (Figures S28 and S29). The stereochemistry of each amino acid was deduced as L-configured based on the ribosomal biosynthetic origin and no epimerization enzymes involved in its biosynthesis.

Compound **3** was isolated as a yellow powder. Its molecular formula was determined to be $C_{42}H_{48}N_8O_8$ based on a protonated ion at *m/z* 793.3663 (calcd for $C_{42}H_{49}N_8O_8^+$, 793.3667, Δ –0.50 ppm) in HR-ESI-QTOF-MS data (Figure S30). Analysis of the ^1H and ^{13}C NMR spectra (Figure S31 and S32), with the aid of the HSQC spectrum (Figure S34), revealed the presence of 40 carbons including six carbonyl carbons, nine nonprotonated sp^2 carbons, 11 sp^2 methines, six sp^3 methines, six sp^3 methylenes, and two aliphatic methyls. These signals and amide protons signals (δ_H 7.92, 8.21, 8.02, 7.23) are characteristic for peptides (Table S4). The presence of a Tyr residue was determined based on the AA′XX′ spin system (δ_H 7.00, 6.59) and benzyl methylene signals (δ_H 2.53, 2.40), along with the HMBC correlations of H_2-25/C-27, H-27/C-29, and H-28/C-26 (Figures S35). A continuous spin system in the aromatic region (δ_H 7.61, 7.13, 7.20, 7.52), a singlet signal for an aromatic proton (δ_H 7.84), and unequivalent aryl methylene signals (δ_H 3.16, 3.13) suggest the presence of a Trp residue (Trp-2). The ^1H–^1H COSY spectrum (Figure S33) showed a constituted spin system including resonances for two methyl doublets (δ_H 7.92, 4.08, 1.50, 1.72, 0.91, 0.88), indicating the presence of a Leu residue. A Gln residue was assigned based on a spin system of an amide proton (δ_H 8.02), α-proton (δ_H 4.13), and two methylene signals (δ_H 2.29, 2.23, 1.96, 1.73) and the HMBC correlation from H-21 to a primary amide carbon C-22 (Figures S35). Furthermore, the 7-substituted Trp (Trp-1) was assigned based on the ^1H–^1H COSY correlations of NH-34/H-34, H-36/H-37, and H-37/H-38, along with the HMBC correlations from H_2-32 to C-34 and C-35, from H-34 to C-33, C-35 and C-40, from H-36 to C-33 and C-40, and from H-37 to C-35 and C-39 (Figure S33 and S35). The amino acid sequence of **3** was determined to be NH_2-Trp-Tyr-Gln-Trp-Leu-CO_2H based on the HMBC correlations from amide protons to carbonyl carbons (Figure S35). The HMBC correlation from H-11 of Trp-2 to C-39 of Trp-1 suggested the presence of a C–N bond between C-39 of Trp-1 and N-11 of Trp-2. This aryl C–N bond was further confirmed by the NOESY correlations of H-11/NH-34 and H-16/H-38 (Figure S35 and S36). The stereochemistry of each amino acid was deduced as L-configured based on the ribosomal biosynthetic origin and no epimerization enzymes involved in its biosynthesis.

Compound **4** was isolated as a yellow powder. Its molecular formula was determined to be $C_{29}H_{35}N_5O_6$ based on a protonated ion at *m/z* 550.2659 (calcd for $C_{29}H_{36}N_5O_6^+$, 550.2660, Δ –0.18 ppm) in HR-ESI-QTOF-MS data (Figure S37). Analysis of the ^1H and ^{13}C NMR spectra (Figures S38and S39), with the aid of the HSQC spectrum (Figure S41), revealed the presence of 29 carbons including four carbonyl carbons, seven nonprotonated sp^2 carbons, seven sp^2 methines, five sp^3 methines, three sp^3 methylenes, and three aliphatic methyls. These signals and amide proton signals (δ_H 7.37, 7.34) are characteristic for peptides

(Table S5). The ^{1}H–^{1}H COSY spectrum (Figure S40) showed a constituted spin system including resonances for two methyls doublets (δ_H 7.37, 4.22, 1.24, 1.41, 0.80, 0.75), indicating the presence of a Leu residue. The ^{1}H–^{1}H COSY of NH-17/H-17 and H-17/H_3-18, along with the HMBC correlation of NH-17/C19 and from H-17/C-16 indicate the presence of an Ala residue (Figure S40 and S42). The ^{1}H NMR and ^{1}H–^{1}H COSY spectra showed the AMX spin system for a 1,2,4-trisubstituted aromatic ring (δ_H 7.87, 7.33, 6.91), signals for an amino proton (δ_H 10.57), an aromatic proton (δ_H 6.96), and a nonequivalent aryl methylene (δ_H 3.12, 2.89). These ^{1}H signals were assigned to protons of a 6-substituted Trp residue based on the HMBC correlations from H_a-21 to C-23 and C-24, from H-23 to C-22, C-24, and C-29, from H-25 to C-27 and C-29, and from H-26 to C-24 (Figure S42). Furthermore, another AMX spin system (δ_H 6.86, 6.81, 6.75) and a benzyl methylene signal (δ_H 2.96) indicated the presence of a 3-substituted Tyr residue, which was further supported by the HMBC cross-peaks of H_2-3/C-4, H_2-3/C-5, H_2-3/C-9, H-8/C-4, H-8/C-6, H-9/C-5, and H-9/C-7 (Figure S42). The amino acid sequence of **4** was determined to be NH_2-Trp-Ala-Leu-Tyr-CO_2H based on the HMBC correlations from amide protons to carbonyl carbons, along with the core peptide sequence of XaaA (Figure S42 and Table S1). The HMBC correlation from H-26 and H-28 of Trp to C-6 of Tyr suggested that C-6 of Tyr and C-27 of Trp are linked through a C–C bond (Figure S42). The stereochemistry of each amino acid was deduced as L-configured based on the ribosomal biosynthetic origin and no epimerization enzymes involved in its biosynthesis.

Antimicrobial assay

Disk diffusion assay were used for the evaluation of the antimicrobial activity of compounds. Compounds **1**, **3** and **4** were dissolved in DMSO at a concentration of 2.56 mg/mL. The ESKAPE pathogens (*Enterococcus faecium*, *Straphylococcus aureus*, *Klebsiella pneumonia*, *Acinetobacter baumannii*, *Pseudomonas aeruginosa*, *Enterobacter cloacae*) were used as reporter strains, which were cultured in LB medium at 37 °C (130 rpm) to an OD_{600} value of ~ 0.6. The cultures were mixed with LB agar to prepare the agar plates in a ratio of 1:100. Trimethoprim and ampicillin were used as positive controls. The 6 mm paper disks that were loaded with 5 µL of each compound, were placed onto the agar plates. The plates were incubated at 37 °C for 16 h. The antimicrobial activity was determined by the size of the inhibition zone.

Anti-inflammatory assays

1) Cell culture and treatments

b.End.3 brain endothelial cells (Merck; 96091929-1VL) were cultivated in High Glucose DMEM+ Glutamax (Gibco; 61965059) medium supplemented with 10% fetal calf serum (FCS) and 1% Penicillin/ Streptomycin. Cells were seeded at 2.5x 10^4 cells/cm^2 growth area in 24 well plates. The day after seeding cells, an inflammatory stimulus was generated with 1 µg/ml Lipopolysaccharide from *E. coli* (Merck; L5293-2ml). Lyophilized atropopeptides were dissolved in DMSO at 10 mg/ml and stored at -20 °C. 8 hours after stimulation, the atropopeptides scabrirubin, compound **1**, compound **3** or vehicle were added into the cell culture media at a final concentration of 1 µg/ml for 60 h (2.5 days) before FACS analysis of inflammation markers.

2) Flow cytometry

After treatments, b.End.3 cells were detached using versene (Gibco; 15040066). After pelleting, Fc-receptors were blocked using TruStain FcX™ (anti-mouse CD16/32) Antibody (Biolegend; 101319) at the recommended dilution in FACS antibody buffer (2% FCS, 0,5mM EDTA in DPBS) for 20 minutes on ice. Subsequently primary antibodies against mouse VCAM1-PE/Cy5.5 (Biolegend; 105716, 1:100 dilution) and ICAM1-PE/Dazzle594 (Biolegend; 116130, 1:250 dilution) in FACS antibody buffer were added to the cells and incubated for 30 minutes on ice. Cells were then washed and subjected to the live/dead cell dye Zombie GreenTM (Biolegend; 423111, 1:200 dilution in DPBS) for 10 minutes on ice. Samples were then filled up

with an equal volume DPBS and immediately analyzed using a S3e (B/Y) cell sorter/ analyzer (Bio Rad). Compensation was performed using the AbC™ Total Antibody Compensation Bead Kit (Thermo Fisher Scientific; A10513) and gates were set using fluorescence- minus- one (FMO) controls. Flow cytometric data was analyzed using the software FlowJo (FlowJo LLC) and statistic analysis was performed using Prism (Graphpad).

Antiviral assays

1) Cell culture

Madin-Darby canine kidney II (MDCK II) cells were maintained in Dulbecco's modified Eagle's medium (DMEM) GlutaMAX, supplemented with 10% FCS and 1% penicillin/streptomycin (P/S). The cells were incubated at 37 °C in a 5% CO_2 atmosphere. All media and supplements were purchased from Thermo Fisher Scientific.

2) Cytotoxity screening in cell culture

Cells were seeded in 96-well plates and incubated at 37 °C in a 5% CO_2 atmosphere until they reached 90% confluence, as determined by microscopy. Subsequently, the cells were treated with compounds **1**, **3** and scabrirubin, ionomycin, DMSO, or methanol. Ionomycin was dissolved in DMSO. All compounds were prepared as 10 mM stock solutions, with a final concentration of 100 µM in the assay. The final concentration of DMSO and MeOH was 1% (vol/vol). Plates were incubated for 48 hours, after which cell viability was assessed by measuring ATP content using the CellTiter-Glo Luminescent Cell Viability Assay (Promega) according to the manufacturer's instructions. The luminescence was recorded using black 96-well plates in a Synergy H4 microplate reader (Biotek/Agilent). Luminescence readings were normalized to the untreated cells, which were set to 100%.

3) Virus preparation

The influenza viruses utilized in this study were strains A/Hamburg/05/2009 (H1N1pdm), A/Hessen/1/2003 (H3N2), B/Malaysia/2506/2004 (Malaysia/B), and B/Massachusetts/71 (Massachusetts/B). The viruses were propagated in MDCK II cells with infection medium (DMEM GlutaMAX) supplemented with 0.2% BSA (bovine serum albumin, Carl Roth), 1% P/S and 1 µg/mL bovine N-tosyl-l-phenylalanine chloromethyl ketone (TPCK)-treated trypsin (Thermo Fisher Scientific) at a multiplicity of infection (MOI) of 0.001–1. Following a 48-hour incubation period, the cell supernatants were centrifuged (2,000 x g, 10 minutes, 4°C) and the clear supernatant subsequently stored at -80°C. Virus titers were determined by plaque assay.

4) Screening of antiviral activity in MDK II cells

MDCK II cells were inoculated with the specified viruses at a multiplicity of infection (MOI) of 0.001, resulting in a quantifiable cytopathic effect after 48 hours. Confluent MDCK II cells were washed twice with phosphate-buffered saline (PBS) and inoculated with the virus in the infection medium. Following a 60-minute incubation period, the cells were washed twice with PBS, after which they were treated with the compounds (stock concentration 10 mM, 100 µM in the assay) or the control aprotinin (10 mM stock in water, 100 µM) in infection medium supplemented with 1 µg/mL bovine TPCK-treated trypsin. Following a 48-hour incubation period at 37°C in a 5% CO_2 atmosphere, cell viability was assessed using the CellTiter-Glo assay. Luminescence values were subtracted from the blank (virus-treated cells with corresponding solvent control) and subsequently normalized to the aprotinin control, which was set to 100% antiviral activity.

DNA sequences

>xamA1

ATGAAGCTAATGCTTACCATCAAAGGTCTGATTTCTTCCAAGAAGAGCCTGAAGTCCTACTCTTGGCCGCACTGG
TACTGA

>xamA2

ATGAAGCTGCTTTTTTCCATCAAAAACCTACTCTCCTCCAAGAAGAGCCTGAAGTCCTCCGCTTGGGAGGGATAC
ATCTCCTACTAG

>skaA

ATGAAGATCGTTCGCTCGCTCAAGAAGAGGATCACCGGAGAGAAGAGCCTCAAGGCGTACGCCTGGTACCACT
GGTACTAG

>xavA

ATGAAGATACTTTTTTCGATCAAGCACATGCTGGCCTCTCGCAAGAGTCTCAAGTCCTCCTCTTGGGGTTTATAT
ATGTAAA

>xatA

ATGAACGTGCTTTTTTCGATCAAGCAATTGCTGTCTTCCAAGAAGAGCCTGAAATCGTACGCCTGGTTACACTGG
TATTGA

>xvvA

ATGCTGGCCTCTCGCAAGAGTCTCAAGTCCTCCTCTTGGCCCGAATATTTTTAA

>xvpA

ATGCTGGCCTCTCGCAAGAGTCTCAAGTCCGCCTCTTGGACCGAATATTTTTAA

>capA

ATGAAGCTCCTTTTCGCCCTCCGCAACCGCGTTGCCGCCGGCAAGAGCCTCAAGGCCAACGCCTGGTACCTCT
GGTACTGA

>nouA

ATGAAGTTCGTTCGTCTCGTGAAGCGGATGCGCACCGAAAAGAGCCTCAAGGCGTACGCCTGGTACGGCTGGA
TCTGA

>jumA

ATGAAGGTTCTCTTTGCCATTCGGCACAAGGTCACGGAGCACCGGAGCCTGAAGGCGTACGCCTGGTACATCT
GGTGGTGA

>lucA

ATGAAGCTCCTCTTCGCCATCCGCAACCGGGTCGCCGCCACCAAGAGCCTCAAGGCCAACGCCTGGTACCTCT
GGTACTGA

>xaa (*xaaA* is highlighted in green)

ATGAAGCTAATGCTTACTATCAAGGAATTGCTTTCTTCCAACAAGAAGAGCTTGAAGTCCTCTGCTTGGGCTTTAT
ACATCTCGTACTGATCGCCATTGCTGCCACTTGCAGTTGCTGCATAACGTGACAATCACCCTCCCTAAAGGGCA
ATCCTTTACTTCTACGATATGCGGTCGGTGAAAGGAACTTCATGTCCTTAACCAGGCATGGGGTTCCTGTTCGAT
GATGGTATTCATTGGGTGGTTTGCGCCGCACGTTCTGCTCTGCGTCGCTTTTTGCATGTAATACGCCCTATGTCT
GTGGATGCTGAGTGCCAGGGATCGGTTTTCCCAACTAGGAGATAGTTCGGATGCCCGCCATCCTGGCTGATCG
CGAGTCGCGAACGACAATCTTGCAGCCGCGTTTGCAGGCCTTGTTGCACGAGCATCTTGGGCAGGATTTATTCC
GTTTGGAAACGAATATGCTCGGCGTCGCTGGTCCGACGCTGATTGATCGTGTGCTCGCGGCCCGGCCCGCGAC
GGAGCACGAACGCCCGACGTTCAAACCCCTGCGAGGTCGCTCGATCCCGCGCACCGAAGCATCGAAGCTGAT
GCAGGCGATCGGCTCCGATGTGCGCGAGGCGCTGAAACGCCCGGTTCCCGTAACGCTCGATCTGTCCGGTTC
GTGGCCGCATGTCGGCCATGTCTATCTGCGCGACCTGTTGCTCCGCGTGGACCCATGGCGCTTGCGCGTGCTG

ATGGACAGAGCCTTGCAATTGACTCCTATGCTGACCTGGATGGTCATTGCCGCAGGTGCGGTATTGCCTCTGAC
GCCGCGGGAGGATGCTTCGGCGCTGGCCAAGCTGATCGCTGCCAAAGGCGCCAGTTGCTACCGCGAGCGCCG
CTACGCCATGGGCCTGTACCGGCGCGCGGCTGCGTCGGTGTGCTTCGCCATCTCCACGCTTGTCGCCAACGC
GCTGTGGCTCGGCTCGCCGTTCGATGCGGCGGCGTCCAATCGCAACATTCTCTACGAAGCGATGCGCTTGCTG
CCCCCGTCCTGGAGTCTGATGCGCAACGCCTCGCCCGAATACGTCGCCTTGGACCCGCGCATCGGCGTTGCC
GACGACGTGCTGATACTGCCCTTTCTCAGTCATCGCGACCCGGCACTGTGGGAGGATCCCGAAGTGTTCCGTC
CAGAACGTTGGAATGGATTGGATCCCGATGCGCTGCCTGGCTATCTCCCGTTCGGACACGCATCCGAACGGTG
TTGGGGCCGGCACATGGTCATGCCGTTGGCCGAGCATTTGCTGGAATTGCTGCGTGCCCAGGAGCTTGTGGTC
AACCCGAAGCAGCGCCGTGCCAGGGTGCCGTTAGACGGGTTGCTGGGTGTGTTCGATGTCCAGGTCGTCCGT
CAGGGCCGTGGATGA

>kat (*katA* is highlighted in green)

ATGAAGATTCTTTTCGCCATCCGTAATGCCGTTTCCGGTCAGAAGAGCCTCAAGAAGAGCGCCTGGTACTTCTG
GTACTAGACCGGCAGAGCCGCCCCGGCCGCCCGCCGGCCGGGACGGCACCGACGGCCCATCGGAGCCTCCG
TATGCCCCTCTCCCCCCTCCCGGCCGCCCGGACCACGGTCTTCGCGCCCCGGCTCGCCGCCCTGCTGCGCGA
GTACACCGGCGAGGACGCCTTCCGCCTGGAGCCCGACACCATGGGCGTCGCCGGCCACGCCCTCGCCGACC
GGATCCTCGCCGCCCGCCGGGCCACCGAGACCGAACGCCCCACCTTCAAGCCGCTGCACGGCCGGTCCATCG
GGCGCACCGAGGCCGCCGCGGTGATGCGGGTCATCGGGCGGGACGTCAGGGAAGCCCTGGAAGGGCCCCTG
CCGAAGGAAGTCGACCTCACCGGCCCGTGGCCCCTCACCGGGCACCTTTTCCTCCGCGACCTCATTCTCGGCG
GCGATCCGCGCCGACTGCGCATTCTGATGAGCCGGAACCTGGAGCTGACCCCGAAACTCACCTGGTCGGTGAT
AGCCGCCGGCGCGGCCGTCCCCTGGCGGAAAAAACCCGGGACGACATCGAGCGGAATTGCCGGCCTGCTGG
CCGAAGCGGACGGCTACCACGACCGCCGCCATGCGATGGGGATCTACCGGAGGGCGGCCGCGCCCGTCTGTT
TCACCGTCTCCACCCTCGTCGCGAACGCCCTCTGGCTGGGCTCGCCCTTCGACGCGGACACCCCCAACCGGAA
CATCCTCCACGAGGCCGCCCGGCTGCTGCCCCCGTCCTGGAACATCCTGCGCTACGCCTCGCCCGAGTACGG
AGCCCTCGACGCGCGCATCGGCGCCGGGGACGACGTACTCGTCCTGCCCCTGCTGTCGCACCGCGACCCCGC
CCTGTGGGAGGAGCCCGACGCGTTCCGGCCCGGGCGGTGGGACCGCCTCGACCCCGAGACCGCCCCGGGCT
ACCTGCCGTTCGGGCACTCCTCCGAGCGCTGCTGGGGCCGGCACATGGTCATGCCGCTCGCGGAACTGCTGC
TGGACCTGATCCGCGGTACCGGACTGGAGGTCGACCCCGCGCAGCGGAAGGCGAAGGTACCGCTGGTCGGG
CTCCTCGGGGTGGAGGACGTACGGCTCACGAAGGCGGCACGGCGTGTCCGTTGAACAGGTCCCGGTCTCCTT
CTTCGACGACCCCCACCCCGCCTACCGCCGCTGGCGGGAGCTGGAGGGCGGGGCGCACCGGGTGCGGATCC
TCGGCGAGGCCCCGCTGGAGGGCTGGGTGGTCACCGGGCACGCCGCCTGCAAGGCCGCCCTCGCCGACCCC
CGGCTGAGCAAGAACGCCGCGACCGAGGCCTTCGACCGCCGCGACGGCTCCGAGGAGGGTCCGGGCCCGGG
CCGCGCGCTGACCGCGCACATGCTCAACTCCGACCCGCCCGCCCACACCCGGCTGCGCCGGCTGGTCCAGCA
GGCGTTCACGGCACGCCGGGTGGCGGCCCTGCGGCCGGTGGTCGAAGGCCACGTGAACCGCCTCCTGGACG
GCCTGGCCCGTACCCCCGAGCCCGACCTCGTCCGGGACTTCGCCGTCCCCCTCCCCCTCGCCGTCGTCTTCG
ACCTGCTCGGCGCCTCCGAGGGGGCCGGGCACATCCTGGAGGCCTGGGCGGCCACCCTCAACGGCGAAGAG
GGCGACGGGGAGGTCTCCGTCCCCACCGCCGAGGCCCTGGTGGGACACATCCGGGCCCTGCTCGCCCACAA
GCGCGCCCACCCCGGCGACGACCTGCTGACCGCACTGATCCACGCCCACGACGACGGGGACCGGCTCAGCG
AGCGGGAGATCACCTCCATGAGCTTCCTGCTGGTGGCGGCGGGCCACCAGACCACCGCCAACCTCATCTCCAA
CGGCGTCCACGCCCTGCTCACCCACCCCCGCGAACTGGCCGCACTCCGCGCCGACCCCTCGCGCACGGGTGC
CTTCATCGAGGAAGTCCTGCGCCACGAGAGCCCGTTCTCCATCGCCACCATGCGCTACGCCACCGAGCCGGTG
ACGATCGACGGCACGACGATCCCCGCCGGGGACTTCGTCCAGATCGCCATGCTGTCGGCCAACCGCGACCCC
GCCGTCTTCCCCGACCCGGACCGCTTCGACCCCTCCCGCCCGGCGACCGGCCACCTGGCCTTCGGCCACGGC
ATCCACCACTGCCTCGGCGCCCCCCTGGCCCGCCTCCAGGCCGAAACCGCCTTCACCACCCTCCTCACCCGCC
ACCCCACCCTCCGCCTGGCCACCCCGACGGCCCGCCCCCAGTGGTGGCGCAACCCCCGCCACCGGGGGCTG
CGCACCCTGCCGGTGCTGCTGGGGTGA

>sva (svaA is highlighted in green)

ATGGAGGAATTTATGAAGCTGGTTCACCTGGTCAACAAGTTCCGTTCGGAGAAGAGCCTCAAGGCATACTCCTG
GTACCAGTGGCTCTGATCGAGTAGTTTTTCACCGGCCCCGGGCCGGCCGGCGACCCCGGCCGGCCCGGGCAG
CCCAGCAACGGAGTAGGAGGTCACCCATGCCAATGCATCGCACCGGCGGGAGCACTCGGGCCGTAGAGTTCG
CGCCGCGCATCGCGGAACTGCTCAGCCGCTATGGCGATGCGCCGCTGTTCCGTCTGGAACCGGACACCATCG
GCATAGCCGACCCCGACCTCATGAACGCCGTACTGAGCGGCAGGCCGGCCAACGAACACGAACGCCCCACCT
TCAAGCCCGTGCAGGGCAGGTCGGTGACGCGCACCGAAGCCTCCGCCTTCATGCAGGCCCTGGGCGCGGACG

TGCGGGCGGCGCTCCAGCGCCCGGTGGACTCGGCGGCCGACGTCAGCGGCCCCTGGCCGCACGCCGGACAC
GTCTATCTCCGCGACCTCCTTTTCGGCCGCGAACACCTGCGCTTCCGCGTTCTGGTGGACCGCAGACTGGAGC
TCACGCCGAAATTGACCTGGGCGGCCGTGGCCTCCGGCGCCGCCGTGCTGGGCAGACCGAAGAGCGACGAAC
CGCTCTCCAAGCTCGCCACCCTCGTTCTCGATTCGCAGAACTACCGCGACCGCCGCCACGCCATGTTCCTCTAC
CGGCGAATGGGCGCGCCGATGTGCTTCACGGTGTCCGCGCTCGTTACCAGCGCCCTGTGGCTCGGCGCCCCG
TTCCGCGACGACATGTCGAATCGGAACATCCTCCTGGAAGCTCTGCGCCTGCTGCCGCCGTCGTGGAACATCC
TGCGCGTGGCCTCCCCGGAATTCCTTTCCGTGGACGAACGCATCGGCCCCGGGGACGACATTCTGCTGTTCCC
CTTGCTGAGCCACCGCAACCCGAAATTCTGGGACGATCCCGAGGAATTCCTCCCCGAACGCTGGGACGAACTC
GACAGCGAGAACCACCCCGGCTACCTCCCCTTCGGCCCCACCAGCGAACGCTGCTGGGCCCGTCACATGGTC
TTCCCCCTCGCCGAACGCCTCCTCGACCTCGTACGCCGCGACGGCCTCGTCGTGAGCCCCGACCAGACCAAG
GTCAAAGTCGAACTGGGCGGGCTGCTGGAATTGTCGGAGGTCCGGATGGTGCGCCCGTGA

>Iau (IauA is highlighted in green)

ATGAAGCTTCTCTTCGCCATTCGCAACAAGGTGTCCGCGCAGAAGAGCCTGAAGGCCAGTGCCTGGTACATCTG
GTACTAGAACGCCTAGTCGTCCCCGCCGCGAGGCGGGGACGACGCCGCTCGCCGATGTCTGTCCGCTCGGCC
TCCCGGAGACTTGCCATGCGCGCCACGGTGTTCCACCCCCGCCTCGCCACCCTGTTCCGGGACCATCTCGGG
CAGGACGTGTTCCGGCTCGAACCCGACACGATCGGGATCGCCGGGCACGAGGTGGCCGACCGGATCCTCGCC
GCCCGCCGGGCCACCGAGACGGAACGGCCCACGTTCAAGCCGCTGCACGGCCGTTCGATCGCGCGGAGTGA
AGCCGCCTCCGTGATGCGGACCGTCGGCAGCGACGTACGCGAGGCGCTGAAGCGGGAACAGCCCCGGCCCG
AGGACGTCGATCTGTCCGGGGAGTGGCCGCTGACGGGGCACATGTTCCTGCGGGACATGGTGCTCGGCAACG
ACCCGTACCGGCTGCGGATGTTGATGAGCCGCAACCTCGAACTCACCACCAAGCTCACCTGGTCGGTGATCGC
GGCGGGCGCCGCCTTCCCGCTGTCGAAGCGGGCCGGGCGGCAGGGCGCGCCGCTGACCTCGCTCGCGGGG
CTGGTGGCCGACGCCGGCACGTATCAGGACCGCCGCTACGCCCTGGGCATGTACCGCCGCGCGGCGGCGCC
CGTGTGCTTCACGGTCTCCACTCTCGTCGCGAACACGCTGTGGCTCGGCTCGCCGTTCGACCCCGAGACGCCG
AACGAGCACCTGCTGTACGAGTCGCTGCGGCTGCTTCCGCCGTCCTGGAACATCCTGCGCAACGCGTGCCCCG
AGTACCCCGCCATCGACGAGCGGATCGGCGCGGGCGACGACGTACTCCTGCTTCCTCTCCTCTCCCACCGGG
ATCCGAAACTGTGGGACTCCCCCGACGAGTTCCGCCCGGAACGCTGGGAGACCCTCGACCCCGACACCGCCC
CCGGCTACCTCCCGTTCGGCCACTCCTCCGAACGCTGCTGGGGCCGGCACATGGTGATGCCGCTCGCCGGGC
TGCTCCTCGACCTCATCCGCGGCTCGGGCCTGGGCATCGACCCCGCGCAGACCACGGCCAAGGTGCCGCTCG
CCGGGCTGCTCGGGGTGGCCGACATACGGGTGACGAAGGTACGGCGTGCCGCCACTGTCTGAACCGACGGCC
GACGGGGTCGAGGTGGTGGACGCGTCCGTCGCCCGCGACCCGCACGCGGCCTACCGGGCGTGGCGGGAGG
CGGGCGGGGTCCGCAAGGTCCGCTTCGCCGGGCCCGTACCGCTGGCCGGGTGGGTCGTCACCGGATACGCG
GCCTGCCGGGCGGCGCTCGCGGATCCGCGGCTGAGCAAGGACGGGGCGACGGAGGCGTACGCCCGGTACG
CGGGACTGCCCACCGGCGGCCCCGGCGGCGGGCTCACCCGCCACATGCTCAACTCCGACCCGCCGCGCCAC
ACCCGCTTACGCCGGCTGGTCCAACGGGCCTTCACCCAGCGGCGGGTGGCCGCGTTGCGGCCCAGGATCGAG
GCCCGGGTCACCGCACTGCTCGACGCCCTGGACAAGGGGACGGGCGAGGGTGCGGGCGAGGTCGATCTGAT
CGAGCGGTTCGCGCTCCCGTTACCCCTGGCGGTCCTCTTCGACCTGCTCGGCACGGAGCCGGACGAGCCCGG
TGTCCTCCAGGTCCGGGGGCACACGGAGGCCGGGGGCCAGGGCGACGGCGAGGTGTCCGTCCCCACGGCGG
AGGCGATCCTGGACCGGCTGCGCGCGCTGGTCGCGCGGAAACGGGCCCGGCCGGGCGACGACCTCCTGTCC
GCGCTCGTCGCCGCCCGCGACGACGGCGACCGGCTCACCGAGGAGGAAGTCACCTCGATGGCTTTCCTGCTG
GTGATCGCGGGCCACCAGACCACCGTCAATCTCGTCGCCAACGGTGTCCACGCCCTCCTCACCCGCCCCGCG
CAGCTCGCCGCCGTACGCGCCGACGCCTCCCTGATACCCGGTCTGGTCGAGGAAGTCCTGCGCTGCGAGAGC
CCGTTCGGCATCGCGACCCTGCGCTACACCACGGAGCCGGTGACCATCGAGGGCACGGCGATCCCGGCCGG
GGAGTTCGTGCAGATCGCGCTCCTCGCCGCGAACCGCGACCCGGAGGTCTTCCCGGACCCCGACCGCTTCGA
CGTCACCCGCGACGCCTCCGGTCACCTGGCGTTCGGCCACGGCATCCACCACTGTCTGGGCGCCCCGCTGGC
CCGCCTCCAGGCCGGCATCGCCTTCACCCACCTCCTGCGCCGCTTCCCCGCCCTCCGCCTCGCCGCCCCGGC
CACGGCCCCCGCCTGGCAGGACAACCCCCGCCACCGCGGCCTCCTCACCCTCCCGGTCCGCCTACACTGA

References:

1. M. H. Hansen, A. Keto, M. Treisman, V. M. Sasi, L. Coe, Y. W. Zhao, L. Padva, C. Hess, V. Leichthammer, D. L. Machell, R. B. Schittenhelm, C. J. Jackson, J. Tailhades, M. Crusemann, J. J. De Voss, E. H. Krenske and M. J. Cryle, Structural Insights into a Side Chain Cross-Linking Biarylitide P450 from RiPP Biosynthesis, *ACS Catal.*, 2024, **14**, 812-826.
2. D. Hanahan, Studies on Transformation of Escherichia-Coli with Plasmids, *J. Mol. Biol.*, 1983, **166**, 557-580.
3. D. J. Macneil, K. M. Gewain, C. L. Ruby, G. Dezeny, P. H. Gibbons and T. Macneil, Analysis of Streptomyces-Avermitilis Genes Required for Avermectin Biosynthesis Utilizing a Novel Integration Vector, *Gene*, 1992, **111**, 61-68.
4. K. F. Chater and L. C. Wilde, Restriction of a bacteriophage of Streptomyces albus G involving endonuclease SalI, *J. Bacteriol.*, 1976, **128**, 644-650.
5. F. Biermann, B. Tan, M. Breitenbach, Y. Kakumu, P. Nanudorn, Y. Dimitrova, A. S. Walker, R. Ueoka and E. J. N. Helfrich, Exploration, expansion and definition of the atropopeptide family of ribosomally synthesized and posttranslationally modified peptides, *Chem. Sci.*, 2024, **15**, 17506-17523.
6. A. Erb, A. Luzhetskyy, U. Hardter and A. Bechthold, Cloning and sequencing of the biosynthetic gene cluster for saquayamycin Z and galtamycin B and the elucidation of the assembly of their saccharide chains, *Chembiochem*, 2009, **10**, 1392-1401.
7. F. Madeira, N. Madhusoodanan, J. H. Lee, A. Eusebi, A. Niewielska, A. R. N. Tivey, R. Lopez and S. Butcher, The EMBL-EBI Job Dispatcher sequence analysis tools framework in 2024, *Nucleic Acids Res.*, 2024, **52**, W521-W525.
8. C. Tumescheit, A. E. Firth and K. Brown, CIAlign: A highly customisable command line tool to clean, interpret and visualise multiple sequence alignments, *Peerj.*, 2022, **10**, e12983.
9. L. T. Nguyen, H. A. Schmidt, A. von Haeseler and B. Q. Minh, IQ-TREE: A Fast and Effective Stochastic Algorithm for Estimating Maximum-Likelihood Phylogenies, *Mol. Biol. Evol.*, 2015, **32**, 268-274.
10. I. Letunic and P. Bork, Interactive Tree Of Life (iTOL) v5: an online tool for phylogenetic tree display and annotation, *Nucleic Acids Res.*, 2021, **49**, W293-W296.
11. G. E. Crooks, G. Hon, J. M. Chandonia and S. E. Brenner, WebLogo: A sequence logo generator, *Genome Res.*, 2004, **14**, 1188-1190.
12. J. Jumper, R. Evans, A. Pritzel, T. Green, M. Figurnov, O. Ronneberger, K. Tunyasuvunakool, R. Bates, A. Zidek, A. Potapenko, A. Bridgland, C. Meyer, S. A. A. Kohl, A. J. Ballard, A. Cowie, B. Romera-Paredes, S. Nikolov, R. Jain, J. Adler, T. Back, S. Petersen, D. Reiman, E. Clancy, M. Zielinski, M. Steinegger, M. Pacholska, T. Berghammer, S. Bodenstein, D. Silver, O. Vinyals, A. W. Senior, K. Kavukcuoglu, P. Kohli and D. Hassabis, Highly accurate protein structure prediction with AlphaFold, *Nature*, 2021, **596**, 583-589.
13. J. Yu, Y. Zhou, I. Tanaka and M. Yao, Roll: a new algorithm for the detection of protein pockets and cavities with a rolling probe sphere, *Bioinformatics*, 2010, **26**, 46-52.
